# The ELSA trial: single versus combinatory effects of non-prohibited beta-2 agonists on skeletal muscle metabolism, cardio-pulmonary function and endurance performance—study protocol for a randomized 4-way balanced cross-over trial

**DOI:** 10.1186/s13063-021-05862-w

**Published:** 2021-12-11

**Authors:** Martina Zügel, Daniel A. Bizjak, Dorle Nussbaumer, Kay Winkert, Kensuke Takabayashi, Johannes Kirsten, Mickel Washington, Gunnar Treff, Jens Dreyhaupt, Luise Steeb, Patrick Diel, Maria Kristina Parr, Jürgen M. Steinacker, Hasema Persch

**Affiliations:** 1 Division of Sports and Rehabilitation Medicine, Department of Internal Medicine II, University Hospital Ulm, Ulm, Germany; 2 Hirakata Kohsai Hospital, Hirakata, Osaka, Japan; 3 Institute of Epidemiology and Medical Biometry, University of Ulm, Ulm, Germany; 4 Institute for Cardiovascular Research and Sports Medicine, Department of Molecular and Cellular Sports Medicine, German Sports University Cologne, Cologne, Germany; 5 Institute of Pharmacy, Pharmaceutical and Medicinal Chemistry, Freie Universität Berlin, Berlin, Germany

**Keywords:** Beta-2 agonists, Asthma sprays, Asthma, Exercise, Anti-doping, Endurance performance, Ergogenic effects, WADA, Skeletal muscle/blood biomarkers, Cardiopulmonary function, Randomized controlled trial

## Abstract

**Background:**

Asthma and/or airway hyper-responsiveness (AHR) are common in elite endurance athletes with a high prevalence rate of beta-2 adrenoreceptor (beta-2) agonists use. Nevertheless, there are data on dose-dependent ergogenic effects of beta-2 agonists suggesting increased muscle strength, endurance and neuromuscular performance. Therefore, most beta-2 agonists belong to the World Anti Doping Agency (WADA) list of prohibited substances and it is tempting to speculate that illegitimate use of beta-2 agonists might be a common practice to boost performance in competitive sports. It is currently unknown whether or not inhaled beta-2 agonists enhance performance by stimulatory effects in skeletal and cardiac muscle.

**Methods:**

The ELSA trial is a double-blinded, placebo-controlled, randomized, balanced, four-way cross-over study. Study participants (*n*=24, 12 ♀, 12 ♂) complete four study arms (i.e. periods with treatment A, placebo; B, salbutamol; C, formoterol; D, formoterol + salbutamol) in random order after an initial preliminary testing session. Participants inhale the study medication 20 min before the 10-min time trial (TT; exercise performance test), where participants cycle 10 min at the highest possible workload. Cardiac output is measured continuously. A skeletal muscle biopsy is collected 3 h after the TT. Study endpoints include measures of skeletal muscle expression of nuclear receptors, hormones and cytokine levels, urinary and plasma concentrations of salbutamol and formoterol, circulating cardiac markers, cardiopulmonary function and exercise performance (average power and peak power during the TT). Blood and urine are collected and respiratory testing is performed 24 h post TT.

**Summary/conclusions:**

This clinical trial evaluates the potential performance-enhancing effects of non-prohibited, not medically indicated inhaled short- and long-acting beta-2 agonists on skeletal muscle gene expression, endocrine regulation, cardiac biomarkers, cardiopulmonary function and acute endurance exercise performance. These data will be used by WADA to adapt the annually published list of prohibited substances (WADA 2021) and will be published in scientific journals.

**Trial registration:**

The trial is registered at the European Clinical Trials Database (Eudra CT) with the number: 2015-005598-19 as well as at the German register for clinical studies (DRKS number 00010574).

**Supplementary Information:**

The online version contains supplementary material available at 10.1186/s13063-021-05862-w.

## Background

Asthma prevalence and the use of asthma medication is up to four-fold higher in athletic populations compared to the general public [1, 2]. There is a high prevalence rate of beta-2 adrenoreceptor (beta-2) agonist use particularly amongst athletes of endurance disciplines and moreover, there is data showing that beta-2 agonist users amongst Olympic athletes have consistently outperformed their competitors [3, 4]. Therefore, it is tempting to speculate that illegitimate use of beta-2 agonists beyond medical reason might be a common practice in competitive sports. Interestingly, the use of asthma medications—especially the combined use of beta-2 agonists and inhaled corticosteroids—has increased from 9.4% (2002) to 12.6% (2009) in Finnish Olympic athletes, whilst the prevalence of physician-diagnosed asthma remained unchanged [5].

To counteract asthmatic episodes, or to prevent exercise-induced asthma and pulmonary oedema, beta-2 agonists are routinely prescribed in sports due to their powerful bronchodilatory effects [6]. The short-acting beta-2 agonist (SABA) salbutamol and long-acting beta-2 agonists (LABA) salmeterol and formoterol are permitted by the World Anti-Doping Agency (WADA) in aerosol form without a therapeutic use exemption (TUE) when taken in maximum allowed therapeutic doses (salbutamol: 1600 μg/24 h in divided doses not to exceed 800 μg over 12 h starting from any dose; formoterol: maximum permitted dose of 54 μg/24 h; salmeterol: maximum 200 μg/24 h, respectively). Since 2021, vilanterol is also permitted in athletes with a maximum inhaled dosage of 25 μg/24 h. All other beta-2 agonists need a TUE [7].

In contrast to beta-2 agonists of older generation such as clenbuterol that require large doses to induce anabolic effects, newer generation beta-2 agonists, such as formoterol and salmeterol, have been shown to elicit an anabolic response even at very low doses in rats [8]. In addition to increasing muscle size and strength, beta-2 agonists have also been shown to affect several aspects of skeletal muscle biology that play important physiological roles in muscle regeneration and energy balance. Hence, they contribute to increased physical performance levels (e.g. modulation of oxidative metabolism, triglyceride lipolysis, glucose transport, glycogenolysis, muscle protein turnover and satellite cell activation) [9]. Therefore, WADA has defined threshold levels for urinary concentrations of salbutamol (1000 ng/ml) and formoterol (40 ng/ml) based on results from pharmacokinetic studies in healthy and asthmatic subjects [10, 11] in order to discriminate between therapeutic intake of non-prohibited beta-2 agonists and doping.

Data on dose-dependent ergogenic effects of beta-2 agonists are scarce and controversial. Some studies have found no significant effects of inhaled beta-2 agonists on aerobic capacity and exercise performance in non-asthmatic athletes [12–14] whilst others report increased muscle strength, endurance and neuromuscular performance [15–17]. One study has shown that the combined inhalation of salbutamol, formoterol and salmeterol increases swim ergometer performance and quadriceps maximal voluntary isometric contraction in elite swimmers with and without AHR [18]. Moreover, high doses of inhaled beta-2 agonists lead to elevated plasma levels of beta-2 agonists and thus to systemic effects like increased cardiac output [19]. However, there is a lack of data regarding the underlying molecular basis of potential performance-enhancing effects of SABA and LABA at doses currently allowed by WADA.

Therefore, the study objective is to investigate molecular, cardiopulmonary and performance effects of WADA permitted doses of beta-2 agonists in endurance athletes during and after an acute bout of high-intensity endurance exercise to explain the abuse potential in high-performance sports and to provide a rationale for effective antidoping control.

## Methods

### Study design

**Fig. 1:**
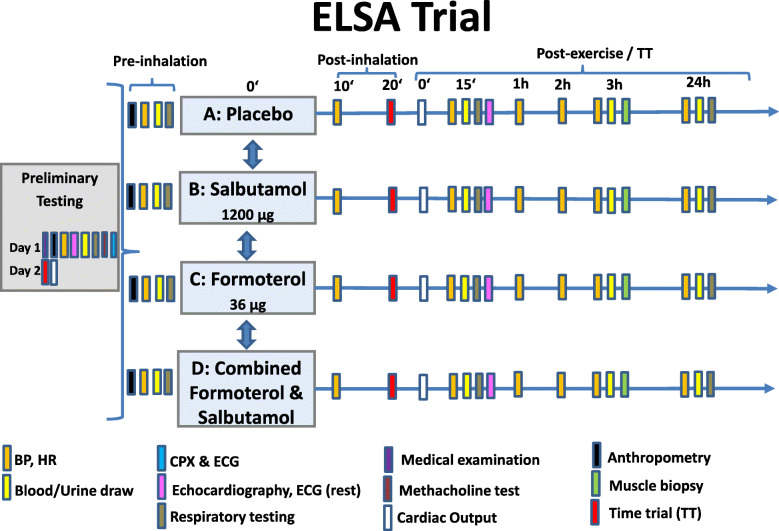
Schematic depicting of the ELSA trial. Study participants (*n* = 24, 12 ♀, 12 ♂) complete four study arms after the initial preliminary testing. The purpose of the preliminary testing (2 days) is to determine participants’ general health and physical fitness. Day 1 consists of a medical examination where the individual medical history will be recorded and also anthropometric measurements, measurements of blood pressure (BP) and heart rate (HR), echocardiography and 12-lead electrocardiogram (ECG) at rest, blood and urine collection, respiratory testing, a methacholine bronchial challenge test and a cardio-pulmonary exercise test (CPX). A time trial (TT) for familiarization purposes of the participants on a bicycle ergometer followed by cardiac output measurements will be performed on day 2 of preliminary testing. Each of the four study arms starts with anthropometric measurements, BP and HR recordings at rest, followed by blood and urine collections and respiratory testing. Afterwards, participants inhale the study medication (spray inhalers) and BP and HR are measured and respiratory testing is conducted 10 min after application of the medication. The TT procedure starts 20 min after the inhalation and begins with a 20-min warm-up followed by a 5-min low-intensity interval before the start of the 10-min TT, where participants cycle 10 min at the highest possible workload starting with a load equalling 90–95% of maximum power obtained in the preliminary CPX. During the TT, cardiac output is measured non-invasively. BP, HR, respiratory testing and echocardiography are recorded 15 min after the end of the TT. HR and BP are also measured 1 h, 2 h, 3 h and 24 h after the end of the TT. Blood and urine are collected and respiratory testing is performed at 3 h and 24 h post TT. A muscle biopsy is collected 3 h after the TT. The time between study arms will be 5–8 days. Note: Symbols are placed according to the chronological order of the tests and examinations

This is a prospective, monocentric, randomized, sex-stratified, double-blinded, placebo-controlled, balanced, four-way cross-over trial. The study is designed to investigate single and additive/synergistic effects of non-medically indicated use of permitted inhaled SABA (salbutamol) and LABA (formoterol) beta-2 agonists at WADA permitted doses on healthy, non-AHR female and male endurance athletes. The outcome measures will focus on skeletal muscle metabolism, endocrine regulation, cardiopulmonary function and endurance performance (Fig. 1). Participants will be recruited from the surrounding sports clubs. Additionally, the department for press and public relations of Ulm University will assist by press releases and calls via the intranet. Potential participants will be pre-selected and ranked according to their probable sports performance capability and eligibility.

Spirit reporting guidelines were used to create this article [20].

### Randomization

Participants (*n* = 24, 12 ♀, 12 ♂) are randomized into the four possible sequences by a stratified block randomization according to sex. Participants’ meeting all the inclusion criteria and none of the exclusion criteria (complete list in supplementary material, Additional file 1) receive the next consecutive randomization number according to the stratum (male/female). Participants take part in all experimental conditions in a four-way cross-over design (Fig. 1). The randomization list is generated by the Institute of Epidemiology and Medical Biometry Ulm University, Germany, using the validated randomization system ROM [21]. A copy of the randomization list is transferred to the central pharmacy at University Hospital Ulm where the clinical pharmacists will prepare the study medication.

### Study medication

The study medication is prepared and packaged in the central pharmacy at University Hospital Ulm. Each medication test kit consists of six blinded spray inhalers, which are medication and/or placebo. These will be provided in blinded packages for the four study arms for each participant (four medication kits per participant containing 24 sprays in total).

For safety reasons, the investigator receives a set of sealed envelopes, one for each randomization number, including the treatment sequence. These envelopes can be opened in emergency situations (such as cardiac rhythm disorder or syncope). Opening the envelope and breaking the code is a major protocol violation and will lead to individual participant exclusion of the study.

### Study procedure

Study participants first undergo thorough preliminary testing on two screening days to determine their health status and physical function. To be eligible for study participation, participants must meet all of the following inclusion criteria:

1. Age: 18 to 45 years
2. Endurance trained with a V̇O_2_max ≥ 52 ml/kg/min in male or ≥ 42 ml/kg/min in female athletes, respectively (V̇O_2_max measured on a bicycle ergometer)
3. A personally signed and dated consent form must be present before any study-related treatments or examinations can take place.

If participants pass the medical examination and fulfil none of the exclusion criteria (complete list in supplementary information, Additional file 1), they are randomly allocated to start in one of the four experimental treatments (Fig. 1—A, placebo; B, salbutamol; C, formoterol; or D, salbutamol + formoterol) after informed consent is obtained. All these actions are carried out at one trial site (Division of Sports and Rehabilitation Medicine, Department of Internal Medicine II, University Hospital Ulm, Germany). Participants will take part in all experimental conditions in a four-way cross-over design (Fig. 1). The time between day 1 and day 2 of preliminary testing will be 1 to 3 days. The time between the preliminary testing (day 2) and the first study arm will be 3 days to 8 weeks.

Study participants will be insured from the time of the written consent until 7 days after the last experimental visit (approximately 9 weeks per participant). Insurer will be HDI-Gerling Industrie Versicherung AG, Riethorst 2, 30659 Hannover.

#### Preliminary testing

The preliminary testing procedure includes medical history assessment, physical examination including HR, BP and anthropometry, 12-lead-electrocardiogram (ECG), echocardiography, laboratory testing including blood and urine draw, respiratory testing, methacholine test as a bronchial challenge test to reliably exclude AHR and a cardiopulmonary exercise test (CPX) to evaluate participants’ V̇O_2_ max (including a continuous incremental test and a subsequent verification test). In brief, the continuous incremental test starts with a workload of 25 Watt. Depending on individual fitness and body mass, mechanical power output increases between 20 and 35 W/min for female participants or between 35 and 45 W/min for male participants, leading to voluntary exhaustion and test termination when cycling cadence falls below 60 rpm after 10–12 min. Highest V̇O_2_ is averaged for 30 s and temporarily accepted as V̇O_2_max [22]. Power at V̇O_2_max (Pmax) is calculated during the same interval. After a break of 15 min, a supramaximal constant-load test with 110% of Pmax is performed lasting approximately 5 min, to verify or correct the temporary V̇O_2_max [23].

All necessary variables are calculated from respiratory gas exchange, ventilation and heart frequency, measured during CPX. At cessation of both tests, participants will be asked to rate their perceived exhaustion using the Borg Scale [24].

A test time trial (TT) is performed during the initial medical examination allowing participants to get familiarized to the exercise test before performing the test during the four experimental study arms. The test is performed on the same cycle ergometer as used during the incremental test. Participant specific settings of the ergometer (saddle and hand bar position) are the same during all trial arms.

#### Experimental study visits

The experimental study visits start between 3 days to 8 weeks after the screening and each study arm will follow within five to eight days (Fig. 1). Participants are asked to avoid any exhausting activity as well as alcohol intake 24 h prior to exercise testing. Moreover, participants should be fasting at least 8 h prior to exercise testing. The examination takes place at the same time of the day in order to minimize the influence of circadian rhythm variations. Adherence to the protocol will be checked and recorded in the Case Report Form (CRF). Protocol modifications (if any) will be communicated to the ethics committee, WADA as well as to the German Federal Institute for Drugs and Medical Devices (BfArM).

At the beginning of each study visit, body core temperature (ear thermometer) is measured to exclude fever. Afterwards, body mass, standing height, BP and HR at rest are recorded followed by respiratory testing, blood and urine collection (Table 1). In women with childbearing potential, a urine pregnancy test will be carried out.

**Table 1:**
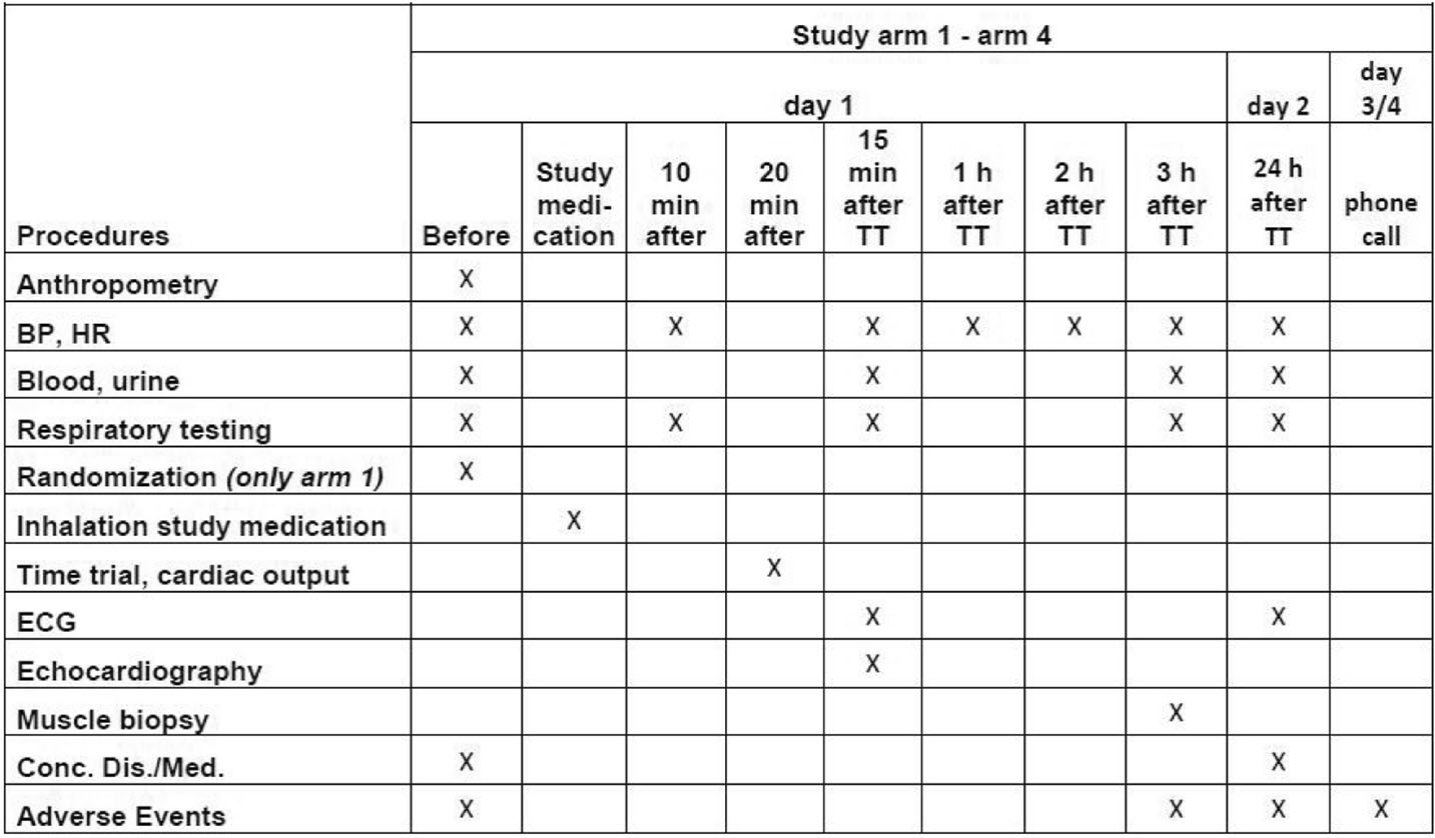
Schedule illustrating enrolment and interventions (SPIRIT figure) of the ELSA trial. Time trial (TT), blood pressure (BP), heart rate (HR), electrocardiogram (ECG), concomitant diseases and/or medications (conc. Dis./Med.)

Afterwards, to standardize the nutritional status, all participants are provided with a defined and standardized meal.

**Fig. 2:**
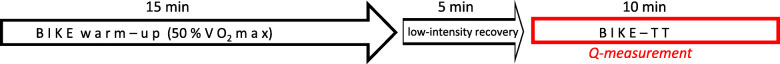
Endurance performance test involving a 20 min warm-up at 50% of V̇O_2_max and a 10 min time trial (TT) separated by a 5 min low-intensity recovery exercise period. Cardiac output (Q) will be continuously measured by the pulse contour method

Ten min after application of the study medication, BP and HR are documented. The TT starts 20 min after the inhalation of the study medication and is performed on the same electronically braked cycle ergometer as used during the preliminary testing. Participants first start a bike warm-up (50% of V̇O_2_max) for 15 min. After 5 min of low-intensity recovery exercise, the 10 min TT on an electronically braked cycle ergometer (Lode Excalibur Sport, Groningen, The Netherlands) starts (Fig. 2).

During the 10-min TT, participants aim to perform the highest average power output possible within 10 min at an even pace. Participants start with a workload equaling to 90-95% Pmax of the previously described incremental test and can increase workload themselves by increasing the cadence. In addition, Borg Scale is used to monitor the rate of perceived exhaustion [24]. Expected values at the end of the test are 18-20. Participants are strongly encouraged to perform the highest average power output possible within 10 min.

To measure BP continuously and non-invasively, a Clearsight® (Edwards LifeSciences Corporation, Irvine, CA, USA) monitor is used. In addition, a small sample of capillary blood (95 μl) is drawn from the ear lobe 0, 5 and 10 min after completion of the TT to measure plasma lactate, blood glucose, and blood gases. HR, BP, respiratory testing and echocardiography are recorded 15 min after the end of the TT. HR and BP are also measured 1 h, 2 h, 3 h and 24 h after the end of the TT. A muscle biopsy is collected 3 h after the TT. Blood and urine samples are collected and respiratory testing is performed 24 h post TT. The time of 5–8 days between each study arm was chosen in order to ensure complete wash-out of the study medication and to avoid adaptation to the exercise protocol.

Measurements that could potentially influence participants` TT performance (e.g. CPX, rebreathing manoeuvres or blood sampling) were excluded to prevent influence on the outcome variable P_TT_. All participants will be blinded to their performance outcome during the TT.

The participants are regularly asked to report any side effects related to the study medications.

### Outcome assessment

Potential ergogenic effects will be investigated by measuring skeletal muscle gene expression, endocrine regulation, urinary and plasma beta-2 agonist concentrations, cardiac biomarkers, cardiopulmonary function and TT performance. Blinded data will be collected in the CRF. Some blinded data cannot be collected in the CRF due to later evaluation (e.g. echocardiographic datasets and molecular markers) and therefore they will be additionally collected in an excel spreadsheet. All data will be entered into a database (double data entry, range checks) and statistically analysed by the Institute of Epidemiology and Medical Biometry Ulm University, Germany.

#### Biological samples

Blood (2.7 ml EDTA Plasma, 7.5 ml Serum, 4.9 ml Lithium-Heparin, 5 ml Citrate Plasma) is collected during the preliminary medical examination (examination of full blood count, GPT, GGT, bilirubin, glucose, creatinine, urea, potassium, sodium, calcium, CRP, TSH, T3, T4, PTT and INR). At the four experimental study visits, blood (2 × 2.7 ml EDTA Plasma, 2 × 7.5 ml Serum, 1 × 9 ml Lithium-Heparin) and urine samples (30 ml) are collected prior to the inhalation of the study medication and 15 min, 3 h and 24 h post-exercise. In addition, a muscle biopsy (approximately 50–150 mg muscle tissue) is obtained from the M. vastus lateralis muscle from participants 3 h after each exercise test (4 biopsies in total).

#### Molecular methods

Differential expression of metabolic and hypertrophic markers will be determined by microarray technology, quantitative real-time PCR (qPCR), Western blot analysis, and immunohistochemical staining. Briefly, RNA and protein from cell lysates will be simultaneously purified using the RNeasy Fibrous Tissue Mini Kit (Qiagen). With the incidence of unexpected analytical findings after the analyses of test parameters, global gene expression profiles (Affymetrix Gene Clariom S PicoArray) are conducted in collaboration with Thermo Fisher Scientific (Department of Genetic Sciences – Microarray) to verify the experimental results. Expression levels of selected genes will be confirmed by qPCR on a LightCycler® 480 PCR System (Roche). Protein levels will be determined from SDS-Page separated protein extracts by Western blot antibody staining for selected markers.

#### Hormonal targets

To investigate the effects of beta-2 agonist treatment on the endocrine system in male and female study participants, variables for muscular growth, cytokine activity, stress responses and hormonal activity will be determined in blood before treatment and 0.25 h, 3 h and 24 h post-exercise using ELISA assays.

#### Urinary and plasma beta-2 agonist testing

Urine (30 ml) is collected before inhalation and at 15 min, 3 h and 24 h post-exercise (4 samples per study arm). As used in routine doping control analyses, a mass spectrometry-based method is applied to analyse biological specimens. Liquid chromatography-tandem mass spectrometry has proved suitable for the detection of beta-2-agonists in urine [25, 26]. Within the project, this methodology will be optimized and adapted to the different matrices. Formoterol and salbutamol, as well as several other beta-2 agonists, are known to be excreted unconjugated and as phase-II metabolites in humans. Thus, the analytical procedure will include both, parent compounds and metabolites.

Urinary excretion profiles indicate that salbutamol reaches maximum urine concentrations within 0–4 h [10]. Formoterol is still detectable in urine up to 72 h after it has been administered [11]. Therefore, to allow sufficient wash-out times of beta-2 agonists, experiments will be spaced 5 to 8 days apart. Peak plasma levels of salbutamol and formoterol are expected within 5 min [27, 28]. Accordingly, beta-2 agonists will be administered (one-time application) by inhalation in the morning (08:00 to 11:00 am) 20 min prior to the beginning of the exercise protocol. Additionally, the urinary density will be measured using a refractometer.

### Endurance performance and cardiopulmonary function

#### Cardiopulmonary testing and TT

Table 2 shows the main variables measured during the exercise tests as well as the most important target variables calculated from the measured variables.

**Table 2:** Exercise tests and respective main variables

Test	Main variables (measured)	Target variable (calculated)
Incremental test	Gas flow	Minute ventilation
	Respiratory gas exchange: FO_2_, FCO_2_	Oxygen uptake, carbon dioxide production
	Power output	Maximal power
	Heart rate	Oxygen pulse
Verification test	See Incremental test	
Time trial	Average power output	Same
	Peak Power	Same
	Blood flow	Cardiac output
	Blood pressure	Blood pressure, peripheral resistance
	Heart rate	Stroke volume
	Blood oxygen saturation: SpO_2_	Same
	Blood lactate concentration	Same
	Blood glucose concentration	Same
	Blood gas analysis: PO_2_, PCO_2_, pH, Na^+^	Same

#### Cardiac output and cardiovascular responses

Participants’ cardiovascular responses are closely monitored by 12-lead ECG, also providing HR data for calculation of stroke volume. To measure blood pressure continuously and non-invasively, a Clearsight® (Edwards LifeSciences Corporation, Irvine, CA, USA) monitor will be used. The Clearsight® device is a totally non-invasive continuous blood pressure monitor based on finger arterial pressure pulse contour analysis. The results are reported to be valid when compared to invasive blood pressure measurements and tend to be more precise than other non-invasive blood pressure measurements [29]. Furthermore, the Clearsight® device allows for continuous estimation of cardiac output during the experiment [30].

#### Echocardiography

Echocardiography is performed during the preliminary medical examination in order to exclude participants with relevant cardiac diseases (e.g., moderate or severe valvular disease, cardiomyopathies). A routine 2-dimensional (2D) transthoracic echocardiography including Doppler- and 2D speckle tracking echocardiography is performed in those participants with an adequate acoustic transthoracic window using an EPIC 7 cardiology ultrasound system and × 5-1-transducer (Philips Healthcare, Andover, Massachusetts) at baseline and 15 min after each TT. All acquired datasets are exported and digitally stored to an EchoView 5.4 workstation (TomTec Imaging System GmbH, Unterschleissheim, Germany) for off-line image analysis with a dedicated software.

#### Cardiac markers

Moreover, determination of circulating cardiac markers will be conducted at four time points: (i) baseline, (ii) 15 min, (iii) 3 h and (iv) 24 h after TT in order to detect changes in cardiac biomarkers associated with the endurance effort.

#### Respiratory testing

Respiratory testing is conducted before inhalation of beta-2 agonists, 0.25 and 24 h post TT. Both a spirometry and a body plethysmography will be performed, in which several standardized variables will be measured (vital capacity, forced expiratory volume in 1 s (FEV1), total lung capacity, reserve volume, specific airway resistance, etc.).

### Ethics and informed consent

The study is performed in accordance with the provisions of the Declaration of Helsinki as revised in 2013, with the International Conference on Harmonization of Good Clinical Practice. The ELSA trial was approved by the ethics committee of Ulm University, Germany (reference number: 64/19).

Prior to participating in the study, the investigator (or a representative) informs the patient verbally and in written form about the scope and purpose, rights, duties, and possible risks and benefits of the study in lay language. For participating in the study, written informed consent by the participant is mandatory. Participants have the right to discontinue study participation without giving a reason at any time. After inclusion into the study, participants are identified by their identification code and randomization number.

### Statistical analysis

For a balanced four-way complete block cross-over design there is a solution involving four sequences, which minimizes the chance and effects of first-order carry-over effects. For each sex, three replications of each line are possible in this Phase I trial, resulting in 12 participants for each sex and a total of 24 participants. Three replications of each line give the statistical potential of estimation of the variance which is needed in mixed model analysis. By neglecting carry-over effects by the procedure of Gaus and Högel [31] there are 12 observations for each of the four treatment combinations expecting enough power for investigation of differences.

Categorical variables such as demographics and medical history data will be described using absolute and relative frequencies. Continuous variables will be summarized using arithmetic mean ± standard deviation or median (25th, 75th percentiles) and minimum/maximum.

A mixed-effects regression model approach according to section 7 in Brown & Prescott [32] will be applied to analyse each endpoint. This approach includes period effects, treatment and sex. Additionally, similar mixed-effects regression models (e.g. separate models for male/female) will be fitted to investigate the stability of the results from the main analysis. All statistical tests performed will be two-sided at a significance level of 5%. Because of the explorative nature of this study, no adjustment for multiple testing will be done. All results from the statistical tests will be regarded as hypothesis-generating only and not as proof of efficacy. The endpoints will be evaluated with a full intention to treat, so that merely withdrawal of informed consent (withdrawal of consent of using all collected data) during the trial will make results unable to be included in the endpoints. It is expected that the rate of withdrawal will be very small. Missing values in measurements will be incorporated in the analysis by using mixed models.

Details of the statistical analyses and possible changes will be included in the statistical analysis plan (SAP, available in supplementary information, Additional file 2).

## Discussion

This investigator-initiated randomized, double-blinded, placebo-controlled, balanced, four-way cross-over trial aims to assess whether inhaled beta-2 agonists enhance endurance performance by stimulatory effects in skeletal and cardiac muscle. Therefore, these results may reveal the abuse potential and performance-enhancing effects of currently permitted inhaled beta-2 agonists in competitive sports. This may provide the WADA with the scientific basis for improved drug regulation and will be used by to adapt the annually published list of prohibited substances [7].

## Trial status

The ELSA trial protocol number is V-31/ 29.08.2019. The trial started in 2020 and it is expected that the human study including biosampling will end at the beginning of 2022.

## Supplementary Information

**Additional file 1.** ELSA trial exclusion criteria.

**Additional file 2.** Statistical Analysis Plan.

**Additional file 3. **SPIRIT checklist for *Trials*.

## Abbreviations

AHR: Asthma and/or airway hyper-responsiveness
Beta-2: Beta-2 adrenoreceptor agonists
BP: Blood pressure
CPX: Cardiopulmonary exercise test
ECG: 12-lead electrocardiogram
FEV1: Forced expiratory volume in 1 second
HR: Heart rate
LABA: Long-acting beta-2 agonists
qPCR: Quantitative real-time PCR
SABA: Short-acting beta-2 agonist
TUE: Therapeutic use exemption
TT: Time trial
V̇O_2_max: Maximal oxygen consumption
WADA: World Anti-Doping Agency
