# Supplementary material for: The ELSA trial: single versus combinatory effects of non-prohibited beta-2 agonists on skeletal muscle metabolism, cardio-pulmonary function and endurance performance—study protocol for a randomized 4-way balanced cross-over trial
Source: Trials. 2021 Dec 11;22:903. doi: 10.1186/s13063-021-05862-w (PMC8665595; doi:10.1186/s13063-021-05862-w)
Supplement: Supplementary file 1 — Additional file 1. ELSA trial exclusion criteria. [file 13063_2021_5862_MOESM1_ESM.docx]

***ELSA trial exclusion criteria***

Subjects must not be included in the trial for any of the following reasons:

1. **Sex and reproductive status:**

- Women of childbearing potential (WOCBP) who are unwilling or unable to use highly effective birth control methods preferably with low user dependency (see Chapter 9. Methods to ensure safety). Men do not have to use birth control methods during participation in the study.
- Women who are pregnant or breastfeeding or are planning pregnancy during course of trial
- Women with a positive pregnancy test on enrolment or prior to investigational product administration

1. **Allergy**

- History of hypersensitivity, contraindication or (serious) adverse reaction of any component of the trial medication; i.e.:
  - Formoterolfumarat-dihydrate
  - Salbutamol-sulfate
  - Lactose
  - Lactose-monohydrate
  - Methacholine-chlorid, or any other cholinergic medication/parasympatomimetic drug
  - Natriummonohydrogenphosphate-dihydrate
  - Natriumhydrogenphosphate-monohydrate
  - Milk protein
- Symptomatic hay fever
- Galactose intolerance

1. **Medical history and concurrent disease:**

- History of bleeding diathesis or active bleeding within the last 30 days
- Patients with an indication for anticoagulant therapy or chronic intake of any anticoagulant therapy
- Any coagulopathy (coagulation testing: INR > 1,5, PTT has to be within the normal range limit according to laboratory testing)
- Thrombocytopenia (platelet count < 30.000/mm³) at screening and before muscle biopsy
- Current liver dysfunction (transaminase level > 2-fold the upper normal range limit)
- Mild kidney dysfunction (estimated glomerular filtration rate < 80 ml/min)
- Medically treated diabetes mellitus (on antidiabetic medication, e. g. metformin, insulin, etc.)
- Recent eye surgery (within 3 months prior to screening)
- Active/florid ulcus ventriculi and/or ulcus duodeni
- Significant neurological disease (e. g. history of stroke, seizure disorder, etc.)
- Significant psychiatric disorder (e. g. acute major depression, manifest bipolar disease, manifest schizophrenia, etc.)
- Known elevated intracranial pressure
- Smokers (smoking ≥ 5 cigarettes/day)
- Known bronchial asthma or any other pulmonary disease which could influence capability to perform exercise testing
- Pathological pulmonary function test*
- Known hyper-responsive bronchial system
- Hypoxaemia (PaO_2_ ≤ 70 mmHg) or hypercapnia (PaCO_2_ ≥ 45 mmHg)
- Dry cough
- Acute severe infection of the respiratory tract
- Use of bronchodilatatory (anti-obstructive) and/or anti-inflammatory medication
- Use of beta-blockers (e. g. propranolol, metoprolol, etc.)
- Pheochromocytoma
- Arterial aneurysm
- Relevant cardiac disease (e. g. angina pectoris, (previous) myocardial infarction, valvular defect, cardiomyopathy; atrioventricular block degree II )
- Sustained hypertension (systolic BP > 140 mmHg or diastolic BP > 90 mmHg) and Sinus-tachycardia (> 100 bpm) at screening
- Medically treated arterial hypertension (e. g. use of ß-blockers, ACE-inhibitors, calcium antagonists, diuretics, etc.)
- Severe hypotension (systolic BP < 90 mmHg or diastolic < 50 mmHg) at screening
- Intake of any medication** (e. g. tricyclic antidepressants, MAO inhibitors, steroids, diuretics, digitalis glycosides, anti-rheumatic drug, anti-cholinergic drug, methylxanthine, etc.) which could influence the results or increase the risk of side effects of the trial medication
- Any planned surgery using halogenated anesthetics during course of trial
- Lactase deficiency
- Glucose-galactose malabsorption
- QT-interval out of normal range*** (for short QT interval in athletes < 320 ms, for prolonged QTc interval in female athletes ≥ 480 ms and in male athletes ≥ 470 ms, respectively)
- Medical history of paroxysmal tachycardia, collapse or syncope
- Symptomatic vagotonia
- Significant rhythm disorders in 12-lead resting electrocardiogram (e. g. atrial fibrillation, atrial flutter, signs of WPW-syndrome, AV-node-reentry tachycardia, ventricular tachycardia, 2nd degree AV block)
- Hyper- or hypothyroidism
- Potassium < 3,4 mmol/l at screening
- Severe systemic disease, such as known malignancies or other comorbid conditions with life expectancy less than one year
- Acute infectious disease or non-infectious inflammation process (e. g. chronic autoimmune disorders)
- Suspected poor capability to follow instructions and cooperate
- Participation in any other clinical interventional trial (drug/device) within less than 30 days prior to screening
- Any other contraindication to perform exercise testing

1. **Subjects who are incapable of giving informed consent**

- Unable to provide informed consent (e. g. severe dementia, or psychosis)
- Prisoners or subjects who are involuntarily incarcerated
- Subjects who are compulsory detained for treatment of either a psychiatric or physical illness (e. g. infectious disease, etc.)

1. **Positive methacholine challenge test:**

- Drop of FEV1 (forced expiratory volume in 1 second) ≥ 20% during challenge testing with a maximum cumulative dose of 471 µg of methacholine-chloride

1. **Positive test at drug screening**

- positive test on amphetamine, methamphetamine, MDMA (Ecstasy), cannabis, cocaine or opiate

* FEV1 < 85% of the target, FEV1/VC < 75%, VC < 80% of the target, TLC < 85% of the target, specific airway resistance (SR tot) > 130% of the target

** see: prohibited medication (section 8.1)

*** QT interval corrected (QTc) for heart rate is measured using Bazett`s formula with heart rates between 60 and 90 bpm; out of normal range parameters in accordance with current, international recommendations for electrocardiographic interpretation in athletes [Sharma, Drezner et al. (2017](#_ENREF_33)).

**Section 8.1: Prohibited medication due to drug interactions

Intake of any of the following medication will lead to exclusion of the study:

- - Bronchodilatatory medication (applied by inhalation or oral; e. g. salbutamol, salmeterol, formoterol, anticholinergics e. g. ipratroprium bromide)
  - Methylxanthine (e. g. theophylline), sympathomimetic drugs (e. g. methlyphenidate, etilefrine, oxilofrine)
  - Leukotriene antagonists, mast cell stabilizers (e. g. nedocromil, cromoglicic acid), antihistamines (e. g. terfenadine, astemizole, mizolastin)
  - Glucocorticoid medication (applied by inhalation or oral) (e. g. budesonide, fluticasone, beclomethasone)
  - Digitoxin or digoxin
  - Diuretics
  - Beta-blockers (e. g. bisoprolol, metoprolol, propranolol)
  - Calcium antagonists (e. g. nifedipine)
  - Macrolides (e. g. erythromycin)
  - Anti-arrhythmic drugs (e. g. quinidine, disopyramide, procainamide,)
  - Antidiabetic medication
  - Monoaminooxidase (MAO)-inhibitors or tricyclic antidepressants, neuroleptic drugs (e. g. promethazine, phenothiazine), L-Dopa, levothyroxine
  - Chronic anticoagulant medication (e. g. acetylsalicylic acid, clopidogrel, phenprocoumon)
  - Cholinergic drugs, cholinesterase inhibitor
  - Application of histamine
  - Non-steroidal anti-rheumatics/anti-inflammatory drugs (e. g. Indometacin)
  - Hexamethonium, tubocurarine
  - Halogenated anesthetics (e. g. halothane, methoxyfluran, enflurane)

To avoid possible nutritional interactions on performance or molecular targets, the participants will be asked to fast at least 8 h before each experimental study visit and not to consume caffeine or any caffeine-related products before participation in any time trial test.
