# Supplementary material for: The ELSA trial: single versus combinatory effects of non-prohibited beta-2 agonists on skeletal muscle metabolism, cardio-pulmonary function and endurance performance—study protocol for a randomized 4-way balanced cross-over trial
Source: Trials. 2021 Dec 11;22:903. doi: 10.1186/s13063-021-05862-w (PMC8665595; doi:10.1186/s13063-021-05862-w)
Supplement: Supplementary file 2 — Additional file 2. Statistical Analysis Plan. [file 13063_2021_5862_MOESM2_ESM.docx]

**Institute of Epidemiology and Medical Biometry**

**Statistical Analysis Plan**

**Single vs. combinatory effects of nonprohibited Beta-2 agonists on skeletal muscle metabolism, cardio-pulmonary function and endurance performance

ELSA-Trial**

EUDRA-CT number 2015-005598-19
DRKS number 00010574

**Version:** 01

**Final date:** 04.03.2021

date

**Content**

[Abbreviations/Definitions 4](#_Toc64446131)

[1 Version History 4](#_Toc64446132)

[2 Trial 5](#_Toc64446133)

[2.1 Trial Summary and Trial Design 5](#_Toc64446134)

[2.2 Biometrical Report 8](#_Toc64446135)

[3 Deviations from Study Protocol 8](#_Toc64446136)

[4 Analysis Sets 8](#_Toc64446137)

[5 Trial Sites 8](#_Toc64446138)

[6 Definitions 8](#_Toc64446139)

[7 Treatment of Data 10](#_Toc64446140)

[8 Listing of Variables 10](#_Toc64446141)

[8.1 Background and Demographic Characteristics at Screening (Pre-Screening, Screening Day 1 and Screening Day 2) 10](#_Toc64446142)

[8.2 Compliance to Study Drug Regimen 12](#_Toc64446143)

[8.3 Further Variables measured at Screening and Treatment Phase 12](#_Toc64446144)

[8.4 Study Termination / Study Conclusion 15](#_Toc64446145)

[8.5 Variables for Analysis of Study Endpoints 15](#_Toc64446146)

[8.6 Variables for Safety Analysis 16](#_Toc64446147)

[9 Statistical Methods 16](#_Toc64446148)

[9.1 Methods for Descriptive Statistics 16](#_Toc64446149)

[9.2 Details of Statistical Analysis 16](#_Toc64446150)

[9.2.1 Analysis of Endpoints 16](#_Toc64446151)

[9.2.2 Sample Size Calculation 17](#_Toc64446152)

[9.2.3 Analysis of Further Variables 17](#_Toc64446153)

[9.2.4 Further Analyses 17](#_Toc64446154)

[9.2.5 Randomization 17](#_Toc64446155)

[9.2.6 Safety Analysis 17](#_Toc64446156)

[10 Listings, Tables, Figures 18](#_Toc64446157)

[11 Data Problems 19](#_Toc64446158)

[12 Software 19](#_Toc64446159)

[13 Use of Results of Statistical Analysis 19](#_Toc64446160)

[14 References 19](#_Toc64446161)

[15 Appendices 20](#_Toc64446162)

[15.1 Individual Participants Data Listings 20](#_Toc64446163)

[15.2 Tables 20](#_Toc64446164)

[15.3 Figures 40](#_Toc64446165)

**Signature Page**

I have read this statistical analysis plan and I agree with performing the statistical analysis according to the procedures described below.

**Statistical Evaluation:**

Dr. J. Dreyhaupt Ulm

Name Place Date Signature

(block letters)

**Coordinating/Principal Investigator:**

Prof. J.M. Steinacker Ulm

Name Place Date Signature

(block letters)

**Study Manager:**

Dr. D.A. Bizjak Ulm

Name Place Date Signature

(block letters)

**Data Manager:**

L. Steeb Ulm

Name Place Date Signature

(block letters)

**Monitoring:**

Prof. M. Flechtner-Mors Ulm

Name Place Date Signature

(block letters)

# Abbreviations/Definitions

| **Abbreviation** | **Definition** |
| --- | --- |
| ADR | Adverse Drug Reaction |
| AE | Adverse Event |
| AHR | Airway Hyper-Responsiveness |
| BfArM | Bundesinstitut für Arzneimittel und Medizinprodukte |
| CRF | Case Report Form |
| CTP | Clinical Trial Protocol |
| SAE | Serious Adverse Event |
| SAP | Statistical Analysis Plan |
| SUSAR | Suspected Unexpected Serious Adverse Reaction |
| TT | Time Trial |
| WADA | World Anti-Doping Agency |

# Version History

Version 01, there are no other legacy versions.

# Trial

## Trial Summary and Trial Design

This is a prospective, monocentric, randomized, sex-stratified, double-blinded balanced four-way cross-over study to investigate dose-dependent additive/synergestic effects of short-acting (salbutamol) and long-acting (formoterol) beta-2 agonists at WADA threshold doses on skeletal muscle metabolism/hypertrophy, endocrine regulation, cardiopulmonary function and endurance performance. Both beta-2 agonists are for inhaling and three puffs each are required. Thus, six inhalers are needed for each participant and each study period in practice. As consequence, the four treatments A, B, C, D are defined as follows:

A (placebo + placebo)

B (salbutamol + placebo)

C (formoterol + placebo)

D (formoterol + salbutamol)

Study participants are healthy, non-AHR male and female endurance athletes. A well-balanced cross-over study design to reduce first-order carry-over adverse effects according to Gaus and Högel is used (Gaus and Hogel 1992). Gaus and Högel showed that the following four sequences have to be used to balance the design for minimization of first order carry over effects:

4 periods (minimal number of cases n = 4)

**period 1 2 3 4**

**Line 1** A D B C

**Line 2** D C A B

**Line 3** C B D A

**Line 4** B A C D

These sequences were replicated 3 times for each sex for control of variation. Study participants were then randomly allocated to start the study in one of the four sequences as shown above by a stratified block randomization. In total, n=24 study participants (12 male/12 female) are planned. In case of drop outs, study participants will be replaced in some situations, see section 13.1 in the study protocol (CTP) for more details. The term ‘period’ is defined as ‘study arm’ in this document.

The following six endpoints are mentioned in the CTP:

1. Changes in muscular expression of nuclear receptors (NR4A 1/2/3)
2. Hormone and cytokine levels and damage markers
3. Urine and plasma concentrations of salbutamol and formoterol
4. Circulating cardiac markers
5. Cardiopulmonary function
6. Average power and peak power during time trial

Safety will be assessed by previous / concomitant disorders and therapies (past 3 months) / concomitant disorders and medications, AEs, SAEs, SUSARs, parameters of vital signs (blood pressure systolic, blood pressure diastolic, and heart rate), parameters of respiratory testing (FVC, FEV1, MEF25, TLC, FRC, RV, sRaw).

The trial required a *screening phase* and a *treatment phase*. The *screening phase* comprises a pre-screening (delivering of study information) and a screening (thorough check of the potential participant at two different days). Table 1 shows the schedule of study procedures during the screening phase.

**Table 1: Schedule of study procedures in the screening phase of the ELSA trial**

| **Procedures** | **Pre- screening** | **Screening  day 1** | **Screening  day 2** |
| --- | --- | --- | --- |
| **Participant Information handed out** | X |  |  |
| **Signed consent form** |  | X |  |
| **Demographic information** |  | X |  |
| **Previous/concomitant disorders/medication** |  | X |  |
| **Inclusion/Exclusion criteria** |  | X | X |
| **Anthropometry** |  | X |  |
| **Vital Signs** |  | X |  |
| **Physical Examination** |  | X |  |
| **Echocardiography, ECG** |  | X |  |
| **Blood, Urine** |  | X |  |
| **Respiratory testing** |  | X |  |
| **Methacholine test** |  | X |  |
| **CPX, VO_2max_ verification test** |  | X |  |
| **Test time trial, cardiac output** |  |  | X |

The study phase follows the screening phase and comprises the four different treatments A, B, C, D in the randomized order (arm 1, arm 2, arm 3, arm 4). Each study arm comprises two days with treatment procedures (‘day 1’ and ‘day 2’; Table 2) and one day with a phone call asking for AEs (‘day 3/4’, Table 3). Each day covers a number of study procedures (Table 3). The time between the study arms is 5 to 8 days.

Table 2: Schedule of study procedures in the treatment phase of the ELSA trial

| **Procedures** | **4 study arms** | | | | | | | |
| --- | --- | --- | --- | --- | --- | --- | --- | --- |
|  | **Study arm 1 day 1** | **Study arm 1 day 2** | **Study arm 2 day 1** | **Study arm 2 day 2** | **Study arm 3 day 1** | **Study arm 3 day 2** | **Study arm 4 day 1** | **Study arm 4 day 2** |
| **Anthropometry** | X |  | X |  | X |  | X |  |
| **BP, HR** | X | X | X | X | X | X | X | X |
| **Blood, urine** | X | X | X | X | X | X | X | X |
| **Respiratory testing** | X | X | X | X | X | X | X | X |
| **Randomization** | X |  |  |  |  |  |  |  |
| **Inhalation study medication** | X |  | X |  | X |  | X |  |
| **ECG** | X | X | X | X | X | X | X | X |
| **Echocardiography** | X |  | X |  | X |  | X |  |
| **Muscle biopsy** | X |  | X |  | X |  | X |  |
| **Time trial, cardiac output** | X |  | X |  | X |  | X |  |

Table 3: Detailed overview of the study arms 1 to 4 in the ELSA trial

| **Procedures** | **Study arm 1 - arm 4** | | | | | | | | | |
| --- | --- | --- | --- | --- | --- | --- | --- | --- | --- | --- |
|  | **day 1** | | | | | | | | **day 2** | **day 3/4** |
|  | **Before** | **Study medi-cation** | **10 min after** | **20 min after** | **15 min after  TT** | **1 h  after  TT** | **2 h  after  TT** | **3 h after TT** | **24 h after TT** | **phone call** |
| **Anthropometry** | X |  |  |  |  |  |  |  |  |  |
| **BP, HR** | X |  | X |  | X | X | X | X | X |  |
| **Blood, urine** | X |  |  |  | X |  |  | X | X |  |
| **Respiratory testing** | X |  |  |  | X |  |  |  | X |  |
| **Randomization *(only arm 1)*** | X |  |  |  |  |  |  |  |  |  |
| **Inhalation study medication** |  | X |  |  |  |  |  |  |  |  |
| **Time trial, cardiac output** |  |  |  | X |  |  |  |  |  |  |
| **ECG** |  |  |  |  | X |  |  |  | X |  |
| **Echocardiography** |  |  |  |  | X |  |  |  |  |  |
| **Muscle biopsy** |  |  |  |  |  |  |  | X |  |  |
| **Conc. Dis./Med.** | X |  |  |  |  |  |  |  | X |  |
| **Adverse Events** | X |  |  |  |  |  |  | X | X | X |

Further information about the trial is in the CTP (Study Protocol Version 31.1 from 29-Jan-2020). The protocol was approved by the ethics committee of Ulm University (No. 64/19, date: 20-May-2019) and the Bundesinstitut für Arzneimittel und Medizinprodukte (BfArM) (No. 4042211, date: 07-Jan-2020). The first participant was enrolled at the 19-Feb-2020; the last participant was recruited at the 03-Aug-2020 (date of enrolment), last participant out was on the 8-Sep-2020 (EOS date). In total, 33 participants were screened and 25 participants were randomized in this study. Eight participants were screening failures.

The CRF was designed using the TeleForm software and can be scanned using this software.

## Biometrical Report

Parts of the statistical analysis (e.g. tables, figures, and summary statistics) will be provided to the Division of Sports and Rehabilitation Medicine, University Hospital Ulm, for preparation of the integrated report. All output will be provided in English language (e.g. title of tables or figures, names of variables in tables).

# Deviations from Study Protocol

The following deviations from the study protocol are known at time of finalizing this plan.

- Cardiac Output: Rebreathing method for peak-cardiac output, stroke volume, O_2_ and total peripheral resistance were planned and included in the CRF, however not performed during the cardiac output measurements.
- Respiratory Testing: sRaw % predicted could not be measured because of technical reasons.
- Cardiac Output: O_2_, total peripheral resistance and SpO_2_ was not measured in this trial.

# Analysis Sets

All endpoints, incl. safety endpoints, will be evaluated with a full intention to treat, so that merely withdrawal of informed consent (withdrawal of consent of using all collected data) during the trial will make results unable to be included in the endpoints. Missing values in measurements will be incorporated in the analysis by using mixed models.

# Trial Sites

The recruitment and treatment of participants were performed in Germany in the University Hospital Ulm, Division of Sports and Rehabilitation Medicine.

# Definitions

For the points in time in the study (see Table 1, Table 2, Table 3) the following abbreviations are used in this SAP (Table 4).

Table 4: Overview of the abbreviations for points in time for arm 1 in the ELSA trial and in the SAS code

| **Abbreviation** |  | **Point in time** |
| --- | --- | --- |
| SCR |  | Screening |
| SCR D1 |  | Screening day 1 |
| SCR D2 |  | Screening day 2 |
| A1D1 |  | Arm 1, day 1 |
| A1D1 before |  | Arm 1, day 1, before inhalation of study medication |
| A1D1 10after |  | Arm 1, day 1, 10 min. after inhalation of study medication |
| A1D1 20after |  | Arm 1, day 1, 20 min. after inhalation of study medication |
| A1D1 15TT |  | Arm 1, day 1, 15 min. after end of time trial |
| A1D1 1hTT |  | Arm 1, day 1, 1 hour after end of time trial |
| A1D1 2hTT |  | Arm 1, day 1, 2 hours after end of time trial |
| A1D1 3hTT |  | Arm 1, day 1, 3 hours after end of time trial |
| A1D2 |  | Arm 1, day 2, 24 h after time trial |
| A1D34 |  | Arm 1, telephone call day 3 or 4 after muscle biopsy |

The arms 2, 3, 4 are similarly labelled: The term ‘A2’, ‘A3’, ‘A4’ is used instead ‘A1’ in the SAS code, e.g. ‘A3D1 15TT’ is used for ‘arm 3, day 1, 15 min. after end of time trial’. The labels ‘SCR’, ‘SCR D1’, SCR D2’, ‘D1’, ‘D1 before’, ‘D1 10after’, ‘D1 20after’, ‘D1 15TT’, ‘D1 1hTT', ‘D1 2hTT’, ‘D1 3hTT’, ‘D2’, and ‘A1D34’ are used in this document as specification of the point in time e.g. for tables containing results for all four study arms. The abbreviations ‘treatment A’, ‘treatment B’, ‘treatment C’, ‘treatment D’ will be used in this document for labelling the four treatments in this document (see part 2.1).

The following notation is used in this SAP:

- the ***bold and italic marked word*** specify the data table in the SAS data base
- the *italic marked word* signs a variable or a value of a variable in the SAS data base

**Endpoints:**

The following six endpoints are specified in the CTP:

1. Changes in muscular expression of nuclear receptors (NR4A 1/2/3)
2. Hormone and cytokine levels and damage markers
3. Urine and plasma concentrations of salbutamol and formoterol
4. Circulating cardiac markers
5. Cardiopulmonary function
6. Average power and peak power during time trial

All endpoints will be investigated as changes, i.e. as differences between different points in time. For more details and calculation, see part 8.5.

**Safety variables:**

Variables for assessment of safety are the following: previous / concomitant disorders and therapies (past 3 months) / concomitant disorders and medications, AEs, SAEs, SUSARs, parameters of vital signs (blood pressure systolic, blood pressure diastolic, and heart rate), parameters of respiratory testing (FVC, FEV1, MEF25, TLC, FRC, RV, sRaw).

**Further variables:**

More details on further variables and parameters are described in parts 8.1 and 8.3.

**Definition of Protocol Violations:**

Opening the sealed envelope in an emergency situation and breaking the code is a major protocol violation and will be listed for statistical analysis. No other further major or minor protocol deviations will be defined in this Phase-I-trial.

**Compliance to Study Drug Regimen:**

Compliance to study drug regimen cannot be measured in this trial. Instead, compliance will be analysed regarding the number of participants who completed all days in all 4 study arms.

**Strata:**

Data will be analysed according to the stratum sex=male vs. sex=female. The variable *SEX* (SAS table ***scr***) will be used for this stratification.

# Treatment of Data

**General remarks:** All raw data will be scanned, verified and stored in Microsoft ACCESS tables using Teleform and finally transferred to SAS datasets. Checks of plausibility and consistency will be carried out by data management of the Institute of Epidemiology and Medical Biometry and if necessary, clarified by sending queries to the Division of Sports and Rehabilitation Medicine, University Hospital Ulm. Data monitoring was performed by the Division of Sports and Rehabilitation Medicine, University Hospital Ulm (Prof. M. Flechtner-Mors).

**Format of date variables:** All date variables are stored in ACCESS date format and converted to SAS date format “DATE9.”.

**Format of time variables:** All time variables are stored in ACCESS as character variables and converted to SAS time format “TIME5.”.

**Missing values:** All values stored in the data base with ‘.’ or ‘ ’ are treated as missing values in the statistical analysis.

# Listing of Variables

Descriptive methods will be applied both for the whole study population and for each treatment A, B, C, D. Additionally, subgroup analyses will be performed for the strata sex=male and sex=female.

## Background and Demographic Characteristics at Screening (Pre-Screening, Screening Day 1 and Screening Day 2)

Background and demographic characteristics will only be provided for the whole study population and for the strata sex=male and sex=female.

**Pre-Screening (pre-screening, CRF page 1):** The frequency of ‘Subject information handed out’ will be calculated.

**Informed consent (screening day 1, CRF page 1):** The frequency (yes/no) will be calculated for the question: ‘Signed informed consent obtained from subject?’ (yes/no).

**Demographic characteristics (screening day 1, CRF page 1):** Demographic characteristics are age, sex, and ethnicity. Age will be calculated as difference from date of randomization (i.e. date of A1D1, variable *A1D1_DATE* (SAS table ***arm1_4***) and date of birth (variable *BIRTHDATE in SAS* table ***scr***). The SAS function yrdif() will be used in the following manner: age=yrdif(birthdate, enroll_date, 'ACT/ACT'). ('ACT/ACT' uses the actual number of days between dates in calculating the number of years. SAS calculates this value as the number of days that fall in 365-day years divided by 365 plus the number of days that fall in 366-day years divided by 366).

**Type of Sport Regularly Performed (screening day 1, CRF page 1):** Number of training sessions/week, training hours/week and previous training years will be analysed separately for endurance, strength and other.

**Medical History (Previous / Concomitant Disorders and Therapies (past 3 months)) (screening day 1, CRF page 2):** Specification, date of onset, treatment and persistency will be listed.

**Inclusion / Exclusion criteria (screening day 1 and 2, CRF pages 3, 4, 12, 14):** Frequencies of fulfilment will be calculated for inclusion and exclusion criteria (part 1 and 2, CRF pages 3, 4, 12). Frequencies of Re-check inclusion and exclusion criteria will be calculated (CRF page 14), see below. The phrase of the exclusion criteria number 50 at CRF page 12 is reverse (*“QTc within normal range (see page 7)”*). Thus, the participant is eligible in case of the answer yes in this criterion.

**Anthropometry / Vital Signs: (screening day 1, CRF page 5):** These parameters are also measured in the treatment phase and are described in more detail in part 8.3 in this document.

**Physical Examination (screening day 1, CRF page 5):** Parameters of physical examination are checks of body systems. For each body system (head and neck, cardiovascular system, respiratory tract, abdomen, extremities and back, skin, neurological and psychiatric disorders, others) the frequencies of ‘Normal’, ‘Pathological’ and ‘Not Done’ and frequency of ‘pathological’ for ‘other body system’ will be calculated. Additionally, the specifications of the other body system will be listed. The parameters of physical examination are measured at screening day 1 (CRF page 5). Further variables for physical examination measured at screening are parameters for left heart, left ventricular ejection fraction, EFSimpson, general regional kinetics and wall motion abnormalities. These parameters are also measured in the treatment phase and are described in more detail in part 8.3 in this document.

**Echocardiography (screening day 1, CRF page 6):** These parameters are also measured in the treatment phase and are described in more detail in part 8.3 in this document.

**ECG (screening day 1, CRF page 7):** See part 8.3 below for detailed information.

**Blood Samples (screening day 1, CRF page 8):** The frequency of citrate plasma will be calculated and for the case that no sample was taken the reason will be listed. Additionally, the frequency of the three others (EDTA plasma, serum, Lithium-Heparin) will be calculated and for the case that no sample was taken the reason will be listed. These parameters are also measured in the treatment phase and are described in more detail in part 8.3 in this document.

**Urine Sample (screening day 1, CRF page 8):** The frequency ‘drug screening’ will be calculated. Furthermore, the frequency of ‘urine given’ and ‘result of pregnancy test’ (only women) will be calculated. These parameters are also measured in the treatment phase and are described in more detail in part 8.3 in this document.

**Respiratory Testing (screening day 1, CRF page 8):** These parameters are also measured in the treatment phase and are described in more detail in part 8.3 in this document.

**Methacholine Challenge Test (screening day 1, CRF page 9):** For each test (Rest, Test 1, Test 2, Test 3, Test 4, Test 5) the continuous variables (FEV1, change (%)) will be analysed and the frequencies for ‘Provocative dose 20% drop in FEV1 (PD20) reached?’ will be calculated. The frequency for the diagnostic result (severe, moderate - severe, moderate, mild - moderate, borderline, normal) will be calculated additionally.

**Cardio Pulmonary Exercise Test (CPX) (screening day 1, CRF page 10, 11):** The frequency for the protocol and the ergometer adjustments (saddle position, saddle height, handlebar position, handlebar height) will be analysed**.** The pedal system & shoes will be listed additionally. The variables ‘total time until exhaustion’, ‘peak power at VO_2max_ (incremental test)’, ‘Preliminary VO_2max_ (incremental test)’, ‘110% of Peak Power (for verification test)’, ‘Time until exhaustion (verification test)’, ‘VO_2peak_’ for the first incremental test will be analysed (variables *CPX1*, *CPX2*, *CPX3*, *CPX3_CALC*, *CPX4*, *CPX5*, *CPX6*, *CPX6_CALC* in SAS table ***scr***; CRF page 10). The frequency for ‘VO_2peak_ equals to Preliminary VO_2max_ ± 8.55%?’ will be calculated (variable *CPX_result1* in SAS table ***scr***; CRF page 10). In case of ‘no’, the parameters from the repeated measurement from the next day will be used for statistical analysis (variables *CPX1b*, *CPX2b*, *CPX3b*, *CPX3b_CALC*, *CPX4b*, *CPX5b*, *CPX6b*, *CPX6b_CALC* in SAS table ***scr***; CRF page 11). Additionally, the frequency of ‘VO_2peak_ equals to Preliminary VO_2max_ ± 8.55%?’ for the next day will be calculated (variable *CPX_result2* in SAS table ***scr***; CRF page 11). The continuous variables for finalization of the CPX will be analysed: ‘Verified VO2max’, ‘VT at VO2max’, ‘Heart Rate at VO2max’ and ‘Self-selected cadence during CPX test (Cad)’ (variables *CPX7*, *CPX7_CALC*, *CPX8*, *CPX9*, *CPX10* in SAS table ***scr***; CRF page 11).

**Test Time Trial (screening day 2, CRF page 13):** These parameters are also measured in the treatment phase and are described in more detail in part 8.3 in this document.

**Cardiac Output (screening day 2, CRF page 13):** These parameters are also measured in the treatment phase and are described in more detail in part 8.3 in this document.

**Post Exercise Blood Gas (screening day 2, CRF page 14):** These parameters are also measured in the treatment phase and are described in more detail in part 8.3 in this document.

**Re-Check Inclusion- / Exclusion Criteria (screening day 2, CRF page 14):** The frequency of ‘All inclusion criteria met’ (yes/no) and ‘No exclusion criteria given’ (yes/no) will be calculated.

**Screening Conclusion (screening day 2, CRF page 14):** The frequency for ‘Participant qualified to continue with treatment phase?’ and the frequencies for the specification in case of ‘no’ qualification (variable *qualified_spec* in SAS table ***scr***) will be calculated and other reasons will be listed (variable *other_spec* in SAS table ***scr***).

## Compliance to Study Drug Regimen

The measurement of compliance to study drug regimen is not possible in this trial.

## Further Variables measured at Screening and Treatment Phase

An overview of the data measured at screening and in the treatment phase shows Table 1, Table 2, and Table 3 in this document. Details of the investigated characteristics are described in the following.

**Anthropometry / Vital Signs:** Parameters of anthropometry are height, weight, BMI and body core temperature (variables *EAR_TEMP*, *HEIGHT*, *WEIGHT*, *BMI* in SAS table ***anthro***). Height and weight were measured at screening day 1 and at day 1 in each study arm 1, 2, 3, 4 (CRF pages 5, 15, 27, 38, 49). The BMI [in kg/m^2^] is calculated from height [in cm] and weight [in kg] as weight/(height*height)*10000. The body core temperature were measured at day 1 in each study arm 1, 2, 3, 4 (CRF pages 15, 27, 38, 49).Parameters of vital signs at screening are blood pressure systolic, blood pressure diastolic, and heart rate (variables *SYST1*, *DIAST1*, *HR1* in SAS table ***vitale***). In case of increased or decreased blood pressure, the parameters from the repeated measurement after 10 minutes will be used for statistical analysis (variables *SYST2*, *DIAST2*, *HR2* in SAS table ***vitale***). The frequency of the investigators decision (subject is qualified to continue) will be calculated. The parameters of vital signs are measured

- at screening day 1 (CRF page 5)
- at day 1 (six points in time, see Table 3 above) in each study arm 1, 2, 3, 4 (CRF pages 15, 17, 19, 22, 23, 27, 29, 31, 34, 35, 38, 40, 42, 45, 46, 49, 51, 53, 56, 57)
- at day 2 in each study arm 1, 2, 3, 4 (CRF pages 25, 36, 47, 58)

**Echocardiography:** The frequency of ‘echocardiography done’ will be calculated (yes, yes partial, no). In case of no, the reason will be listed. The sonographer initials will be listed. In case of only partial echocardiography, the frequency for the reasons will be calculated and the specification for problems of equipment and other reasons will be listed. The assessments of picture quality will be calculated separate for parasternal short, parasternal long and apical. The frequency for urgent findings and additional findings will be calculated and the specification for others will be listed. The parameters for echocardiography are measured at screening day 1 (CRF page 6) and at day 1 in each study arm 1, 2, 3, 4 (CRF pages 20, 21, 32, 43, 54).

Left heart: The frequency of axis will be calculated and all continuous variables (AoB, LA, LA 4CHV, IVSd, LVIDd, LPWd, IVSs, LVIDs, LPWs, IVSd (a4K), LVEDD (a4K), Ao. asc.) will be analysed. Comments will be listed. Left ventricular ejection fraction: The frequency of LV visual assessment, diastolic dysfunction, 3D-dataset acquired and general regional kinetics will be calculated. Continuous variables for EFSimpson (4 CHV, 2 CHV, biplane, LV EDV biplane, LV ESV biplane, GLS-2D) and Diastolic dysfunction (E, E’lat, A, E’med) will be analysed. Description of wall motion abnormalities will be listed. The parameters for left heart, left ventricular ejection fraction, EFSimpson, general regional kinetics and wall motion abnormalities are measured at screening day 1 (CRF page 7) and at day 1 in each study arm 1, 2, 3, 4 (CRF pages 21, 22, 33, 44, 55).

**ECG:** The frequency of rhythm disorders will be calculated and specifications will be listed. QTc time (continuous) will be analysed. The frequencies of ‘Study exclusion necessary?’ and ‘Subject qualified to continue?’ will be calculated. These parameters are measured at screening day 1 (CRF page 7) and at day 1 and 2 in each study arm 1, 2, 3, 4 (CRF pages 22, 26, 33, 37, 44, 48, 55, 59). In study arm 4, day 2 there are no questions ‘Study exclusion necessary?’ and ‘Subject qualified to continue?’.

**Blood Samples:** Frequency of the three different kinds (EDTA plasma, serum, Lithium-Heparin, citrate plasma) will be calculated and for the case that no sample was taken the reason will be listed. These parameters are measured at screening day 1 (CRF page 7) and at day 1 and 2 in each study arm 1, 2, 3, 4 at different points in time (CRF pages 16, 19, 24, 25, 28, 31, 35, 36, 39, 42, 46, 47, 50, 53, 57, 58).

**Urine Sample:** Frequency of ‘urine given’ will be calculated. The reason will be listed for the case that no sample was taken. These parameters are measured at screening day 1 (CRF page 8) and at day 1 and 2 in each study arm 1, 2, 3, 4 at different points in time (CRF pages 16, 19, 24, 25, 28, 31, 35, 36, 39, 42, 46, 47, 50, 53, 57, 58). Additionally, the frequency of ‘result of pregnancy test’ (only women) will be calculated which is measured at screening day 1 (CRF page 8) at day 1 in arm 1, 2, 3, 4 (CRF pages 16, 28, 39, 50).

**Respiratory Testing:** The continuous variables (FVC, FEV1, MEF25, TLC, FRC, RV, sRaw) will be analysed both as measurements and -with exception of sRaw- as % predicted. These parameters are measured at screening day 1 (CRF page 8) and at day 1 and 2 in each study arm 1, 2, 3, 4 at different points in time (CRF pages 16, 20, 26, 28, 31, 37, 39, 42, 48, 50, 53, 59).

**Time Trial:** The frequency for the question ‘Ergometer adjustment identical to CPX test = yes’ will be calculated. Furthermore the variables ‘Power at 90% VO_2max_ (P90)’, ‘Self-selected cadence during incremental test (Cad)’, and ‘Linear factor = P90 / Cad² x 10’ will be analysed as continuous variables. The frequency for the question ‘Test completed’ (yes/no) will be calculated. In case of ‘no’ the reasons will be listed, the duration of the test will be listed or analysed as continuous variable (as appropriate) and the frequency for the question ‘Adjustment of linear factor’ (yes/no) will be calculated and in case of ‘no’ the new linear factor will be listed or analysed as continuous variable (as appropriate). The ‘Average Power Output’ and the ‘Relative average Power Output’ will be analysed as continuous variables. The parameters for Time Trial are measured at screening day 2 (labelled as Test Time Trial, CRF page 13) and at day 1 in each study arm 1, 2, 3, 4 (CRF pages 18, 29, 40, 51).

**Cardiac Output:** The variables ‘Peak-cardiac output’, ‘Total mean power’, ‘Heart rate’, ‘Stroke volume’, ‘O_2_’, ‘Mean arterial pressure’, ‘Total peripheral resistance’, ‘SpO_2_ ‘for the Pulse-contour method will be analysed as continuous variables. The parameters for Cardiac Output are measured at screening day 2 (CRF page 13) and at day 1 in each study arm 1, 2, 3, 4 (CRF pages 18, 30, 41, 52).

**Post Exercise Blood Gas:** The variables ‘Lactate’, ‘Glucose’, ‘PO_2_‘, ‘PCO_2_‘, ‘pH‘ will be analysed as continuous variables for the minutes 0, 5, and 10. These parameters are measured at screening day 2 (CRF page 14) and at day 1 in each study arm 1, 2, 3, 4 (CRF pages 19, 30, 41, 52).

**AEs / SAEs in the screening phase / treatment phase:** AEs and SAEs in the screening phase and treatment phase will be listed in tabular form. AEs / SAEs are documented on separate pages on the CRF. There are different AE-forms for screening and treatment phase. SAEs are additionally documented on a separate form provided by the ZKS Ulm and stored in a separate data base. For statistical analysis, a separate data base will be provided after SAE reconciliation by the Division of Sports and Rehabilitation Medicine, University Hospital Ulm.

**Pre-Study Check Before Inhalation of Study Medication:** The frequency (yes/no) will be calculated for the following questions: ‘Signed informed consent obtained from subject and still valid?’, ‘Did any changes in concomitant disease / treatment occur since last visit?’, ‘Did any Adverse Events occur since last visit?, ‘Prior to each exercise testing: 24h: exhausting activity and alcohol consumed? 8h: fasting conditions? 2h: caffeine or caffeine-related products consumed?’ and ‘Standardized meal given?’ will be calculated. These questions are asked at start of day 1 in each study arm 1, 2, 3, 4 (CRF pages 15, 27, 38, 49).

**Muscle Biopsy:** The frequency (right leg/left leg) for the question ‘Muscle biopsy of m. vastus lateralis taken from right/left leg’ and the frequency (yes/no) for the question ‘Did the muscle biopsy occur according to protocol?’ will be calculated. The questions for muscle biopsy are asked at day 1 in each study arm 1, 2, 3, 4 (CRF pages 24, 35, 46, 57).

**Variables measured 24 hours after end of time trial:** The frequency (yes/no) will be calculated for the following: ‘Did any changes in concomitant disorders / medications occur since last visit?’, ‘Did subject use any self-medication for post-exercise conditions?’ and ‘Did any side effects occur since last visit?’ will be calculated. Furthermore, the frequency of prescribed medication (yes/no) in case of self-medication for post exercised conditions will be calculated and the name of the doctor will be listed. The variables measured 24 hours after end of time trial are documented at start of day 2 in each study arm 1, 2, 3, 4 (CRF pages 25, 36, 47, 58).

**Side effects:** Asking for side effects is at the end of day 1 (question: ‘Did any side effects occur during day 1?’), at start of day 2 (question: ‘Did any side effects occur since last visit?’), and at the telephone call 2 or 3 days after muscle biopsy (question: ‘Did any side effects occur during day 1?’). The frequency (yes/no) will be calculated for each of these questions (CRF pages 24, 25, 26, 35, 36, 37, 46, 47, 48, 57, 58, 59).

**Concomitant Disorders / Medications:** Specification, date of onset, treatment and persistency will be listed. These data are documented on separate CRF pages at the end of the CRF.

**Further echocardiography data:** Further echocardiography data are measured at screening and at day 1 in each study arm 1, 2, 3, 4. These data are not documented on the CRF and cover the following parameters: left ventricle: end-diastolic volume (ml), left ventricle: end-systolic volume (ml), left ventricle: ejection fraction (%), left ventricle: endocardial global longitudinal strain (%), left ventricle: ShapeFn (%), left ventricle: myocardial global longitudinal strain (%), left ventricle: global radial strain (%), right ventricle: end-diastolic cross-sectional area (cm^2^), right ventricle: end-systolic cross-sectional area (cm^2^), right ventricle: fractional area change (%), right ventricle: endocardial global longitudinal strain (%), left atrium: end-diastolic volume (ml), left atrium: end-systolic volume (ml), left atrium: ejection fraction (%), left atrium: endocardial global longitudinal strain (%), left atrium: ShapeFn, left atrium: fractional area change (%), right atrium: end-diastolic volume (ml), right atrium: end-systolic volume (ml), right atrium: ejection fraction (%), right atrium: endocardial global longitudinal strain (%), right atrium: ShapeFn, right atrium: fractional area change (%),Comments echo data. These data were provided by the Division of Sports and Rehabilitation Medicine, University Hospital Ulm, as additional Excel file. This Excel file was converted to a SAS table echo_sportmedizin.sas7bdat. All mentioned variables with exception of the comments are continuous. The comments on echo data will be listed.

**Data from microarray and pathway analysis:** These data are not documented on the CRF and will be provided as additional Excel file. The structure and content of these data is not known at the finalization of this SAP. The statistical analysis of these data will be performed out of the Institute of Epidemiology and Medical Biometry.

## Study Termination / Study Conclusion

For evaluation of study conclusion, the frequencies (yes/no) for the question “Did subject complete the study as planned?” will be calculated (variable *compl_planned* in SAS table ***eos***).

Furthermore, the frequencies for the reasons for no planned study conclusion (Withdrawal of Informed Consent, Non-compliance by the participant, Pregnancy, Adverse Event, Opening sealed envelope due to an SAE / SUSAR, Lost to follow-up, Other reason) will be calculated (variable *prem_end* in SAS table ***eos***). In case of other reason, the specification will be listed (variables *prem_other_spec1* and *prem_other_spec2* in the SAS table ***eos***). Additionally, remarks at the study conclusion page (CRF page 60) will be listed (variables *remark1*, *remark2*, *remark3*, *remark4* in the SAS table ***eos***).

## Variables for Analysis of Study Endpoints

The following six endpoints are specified in the CTP:

1. Changes in muscular expression of nuclear receptors (NR4A 1/2/3)
2. Hormone and cytokine levels and damage markers
3. Urine and plasma concentrations of salbutamol and formoterol
4. Circulating cardiac markers
5. Cardiopulmonary function
6. Average power and peak power during time trial

Details for 1: Muscular expression of nuclear receptors (NR4A 1/2/3) is a result of the muscle biopsy (e.g. gene expression data, protein concentrations; all parameters are continuous) and is measured once in each study arm. These data are not documented on the CRF and will be provided by the Division of Sports and Rehabilitation Medicine, University Hospital Ulm, as separate data file. Further information is not known at the time of finalization of this SAP (see part 11).

Details for 2: Hormone and cytokine levels and damage markers are continuous parameters and are measured at four points in time in each study arm. These data are not documented on the CRF and will be provided as separate data file. For statistical analysis, all possible differences to baseline per study arm will be calculated for each parameter: ‘AxD1 15TT’ minus ‘AxD1 before’, ‘AxD1 3hTT’ minus ‘AxD1 before’, ‘AxD2’ minus ‘AxD1 before’), x labels the study arm, x=1, 2, 3, 4. Further information is not known at the time of finalization of this SAP (see part 11).

Details for 3: Urine and plasma concentrations of salbutamol and formoterol are continuous parameters and are measured at four points in time in each study arm. These data are not documented on the CRF and will be provided as separate data file by the laboratory at the Institute of Pharmacy (Freie Universität Berlin). For statistical analysis, all possible differences to baseline per study arm will be calculated for each parameter: ‘AxD1 15TT’ minus ‘AxD1 before’, ‘AxD1 3hTT’ minus ‘AxD1 before’, ‘AxD2’ minus ‘AxD1 before’), x labels the study arm, x=1, 2, 3, 4. Further information is not known at the time of finalization of this SAP (see part 11).

Details for 4: Circulating cardiac markers are continuous parameters and are measured at four points in time in each study arm. These data are not documented on the CRF and will be provided as separate data file by the Division of Sports and Rehabilitation Medicine, University Hospital Ulm. For statistical analysis, all possible differences to baseline per study arm will be calculated for each parameter: ‘AxD1 15TT’ minus ‘AxD1 before’, ‘AxD1 3hTT’ minus ‘AxD1 before’, ‘AxD2’ minus ‘AxD1 before’), x labels the study arm, x=1, 2, 3, 4. Further information is not known at the time of finalization of this SAP (see part 11).

Details for 5: Cardiopulmonary function comprises the parameters for Respiratory Testing and Echocardiography and is measured at three points in time in each study arm. More details are described in parts 8.1 and 8.3. For statistical analysis, all possible differences to baseline per study arm will be calculated for each parameter: ‘AxD1 15TT’ minus ‘AxD1 before’ and ‘AxD2’ minus ‘AxD1 before’), x labels the study arm, x=1, 2, 3, 4.

Details for 6: The average power during time trial is documented as average power output (in W) and relative average power output (in W/kg body mass) (variables *TT_APO* and *TT_RPO* in table ***tt***, see CRF pages 13, 18, 29, 40, 51) and will be analysed as continuous variable. The peak power during time trial is not documented on the CRF and was provided by the Division of Sports and Rehabilitation Medicine, University Hospital Ulm) as separate data file (Excel). Average power and peak power are measured once in each study arm.

## Variables for Safety Analysis

Variables for safety analysis are

- Blood pressure (for more information see section ‘Anthropometry / Vital Signs’ in part 8.3 above)
- Results from Respiratory Testing (for more information see section ‘Respiratory Testing’ in part 8.3 above)
- Information about adverse events, serious adverse events (SAE), SUSARs (see part 8.3 above)

**Serious adverse events (SAE):** All serious adverse events are coded by Division of Sports and Rehabilitation Medicine, University Hospital Ulm, using MedDRA.

# Statistical Methods

## Methods for Descriptive Statistics

**Continuous variables:**

Continuous variables will be summarised using the following standard descriptive summary statistics (if appropriate): number of observations, arithmetic mean, standard deviation, minimum, lower quartile, median, upper quartile, maximum. Furthermore, graphical methods will be used, e.g. Boxplots, plots of time course of mean +/- standard deviation resp. median +/- ½⋅inter quartile range for variables of the endpoint. In case of too few measurements, the single measured values will be shown.

**Categorical variables:**

Categorical data will be described using absolute and relative frequencies. Missing data will be treated as separate category in descriptive statistical analysis.

## Details of Statistical Analysis

### Analysis of Endpoints

Each continuous endpoint will be analysed using a mixed effects model approach including period effects, treatment, and sex, according to section 7 in Brown and Prescott (Brown and Prescott 2015). The CTP suggested using the participant as fixed effect. The following mock SAS code was suggested in the CTP for the main analysis of each endpoint (*outcome* denotes an arbitrary endpoint described in part; treat, sex, participant and period are given in the data set):

PROC MIXED;

CLASS *treatment sex period participant*;

MODEL *outcome*=*treatment sex participant period*;

LSMEANS *treatment* /DIFF PDIFF CL;

RUN;

Because of model fitting problems (sex and participant as fixed effects in a joint model are not possible), the participant will be included as random effect instead. The SAS code is as follows:

PROC MIXED;

CLASS *treatment sex period participant*;

MODEL *outcome*=*treatment sex period*;

LSMEANS *treatment* /DIFF PDIFF CL;

RANDOM *participant;*

RUN;

Separate models for male/female will be provided for each endpoint to investigate sex differences. Sex as fixed effect will be excluded in these models. All statistical tests performed will be two-sided at a significance level of 5%. Because of the explorative nature of this study, no adjustment for multiple testing will be done. All results from the statistical tests will be regarded as hypothesis generating only, and not as proof of efficacy. All endpoints will be evaluated with full intention to treat.

### Sample Size Calculation

Full details of the sample size calculation are in the CTP at page 10, 21, 48.

### Analysis of Further Variables

Details of further variables are provided in part 8.3. For analysis of categorical data, absolute and relative frequencies will be calculated. Analysis methods for continuous variables are described in part 9.1.

### Further Analyses

Subgroup analyses (similar mixed effects regression models which will be used in the analysis of the endpoints) will be performed for the strata sex=male and sex=female.

### Randomization

Participants were randomly assigned to one of the four possible treatment sequences (lines 1, 2, 3, 4: ‘A D B C’, ‘D C A B’, ‘C B D A’, ‘B A C D’, see part 2.1 above) according to their stratum (male vs. female). Further information about randomization and replacement of participants who not complete the study is in the CTP (parts 12.1, parts 13.1).

### Safety Analysis

**Analysis of Previous / Concomitant Disorders and Therapies (past 3 months) / Concomitant Disorders and Medications:** Listings of the following parameters will be provided: Disorder number and specification, date of onset, treatment (yes/no) and in case of yes: specification of the treatment, persistency.

**Analysis of AEs / SAEs / SUSARs:** There are only very few AEs / SAEs / SUSARs in this trial. AEs and SAEs will be listed in tabular form. For AEs, it will be distinguished between screening phase and treatment phase:

- AE in screening phase: symptoms of AE, AE leads to exclusion from screening phase (yes/no), start date and time, end date and time, continuing / unknown, is AE a serious event/reaction (SAE/SAR)? (yes/no), causality, severity according to CTCAE, relationship to study medication (methacholine) and in case of yes: is ADR an expected event? (yes/no), action taken (according to ICH E2B), outcome, comments
- AE in treatment phase: study arm, random number, symptoms of AE, AE leads to study exclusion (yes/no), start date and time, end date and time, continuing / unknown, is AE a serious event/reaction (SAE/SAR)? (yes/no), Opening of sealed envelope? (yes/no) and in case of yes: opening date, causality, severity according to CTCAE, relationship to study medication (salbutamol / formoterol) and in case of yes: is ADR an expected event (yes/no)?, action taken (according to ICH E2B), outcome, comments

For SAEs, the following parameters will be listed: Personalized data (study screening number, study subject number (for SAE in treatment phase only), random number (for SAE in treatment phase only), dose number (equivalent to the study arm; for SAE in treatment phase only), date of birth, sex, initial report / follow-up report number), description of serious adverse event (SAE) (specification of description of SAE, Diagnosis, Therapy/Treatment, Course, MedDRA Code, start date SAE, date of discovery, CTC AE grade, is SAE expected? (yes/no), severeness, study medication, reason for SAE, outcome of SAE, stop date of SAE in case of outcome=resolved), SAE relevant concomitant medication and diseases (specification of medication and daily dose, start date, stop date, application, indication, causality assessment SAE/trial medication for at most four medications), medical history (specification of relevant concomitant disease, anamnestic characteristics), report (Does SAE lead to study exclusion? (yes/no).

Analysis of SUSARs: separate Listing of AEs in case of unexpected ADR.

Furthermore, the proportion of participants with at least one AE / SAE / SUSAR (incl. 95% confidence interval) will be calculated for each study arm.

**Analysis of Vital Signs:** For each of the parameter blood pressure systolic, blood pressure diastolic, and heart rate the time course will be analysed descriptive in separate tables (one for each parameter) that contains descriptive statistics for whole study population and for each study arm (A, B, C, D). The seven points in time for each study arm (‘D1 before’, ‘D1 10after’, ‘D1 15TT’, ‘D1 1hTT', ‘D1 2hTT', ‘D1 3hTT’, ‘D2’) are the columns of these tables. For a detailed explanation of these points in time, see Table 4 and the additional information for this table.

**Analysis of Respiratory Testing:** For each of the parameter FVC, FEV1, MEF25, TLC, FRC, RV sRaw (measurements and -with exception of sRaw- % predicted values) will be analysed. The time course will be analysed descriptive in separate tables (one for each parameter) that contains descriptive statistics for whole study population and for each treatment (A, B, C, D). The three points in time for each study arm (‘D1 before’, ‘D1 15TT’, ‘D2’) are the columns of these tables. For a detailed explanation of these points in time, see Table 4 and the additional information for this table.

The appendix (part 15.2) shows a list of tables which will be provided for safety analysis.

# Listings, Tables, Figures

An overview of all planned listings tables and main figures shows the appendix (parts 15.1, 15.2, and 15.3).

# Data Problems

The results for four out of the six endpoints (endpoints 1., 2., 3. and 4., see above) are not documented on the CRF. These data will be provided by different institutions (e. g.: central facility for clinical chemistry (Ulm University), laboratory and muscle laboratory (Division of Sports and Rehabilitation Medicine), laboratory of the German Sport University Cologne, laboratory at the Institute of Pharmacy (Freie Universität Berlin). These different institutions will provide these data via additional data file (e.g. Excel sheet, Access database). The data structure and data quality is not known yet. The quality of these data will be checked as good as possible.

Further data problems are not known at the time of finalisation of this SAP.

# Software

All statistical analyses will be performed using SAS, version 9.4 (SAS Inc. Cary/NC, USA) under Windows 10. SAS-macros developed at the Institute of Epidemiology and Medical Biometry will be used for analyses, if applicable. For quality assurance, an overview of all analysis programs will be created (name of SAS-program, name of author(s), and name of person who reviewed the program).

# Use of Results of Statistical Analysis

Parts of the statistical analysis (e.g. tables, figures, and summary statistics) will be provided to the Division of Sports and Rehabilitation Medicine, University Hospital Ulm. Scientific contributions (e.g. article, oral presentation, and poster) will be prepared by the Division of Sports and Rehabilitation Medicine, University Hospital Ulm or other institutions. (At least) one employee from the Institute of Epidemiology and Medical Biometry will be mentioned as co-author in each scientific contribution. The employee will be informed about the content of each scientific contribution.

# References

Brown, H. and R. Prescott (2015). Applied Mixed Models in Medicine. Wiley.

Gaus, W. and J. Hogel (1992). "Balanced designs for multiple crossover studies." Drug Research 42(2): 163-172.

# Appendices

## Individual Participants Data Listings

Will not be provided. Instead, the final database (SPSS files or SAS files) will be provided.

## Tables

**Study information, screening, participant demographics, participant status at screening, and screening conclusion**

Table A 1: Schedule of study procedures in the screening phase (this table is already included in the
CRF)

Table A 2: Schedule of study procedures in the study phase (this table is already included in the CRF)

Table A 3: Detailed overview of the study arms 1 to 4 (this table is already included in the CRF)

Table A 4: Pre-Screening and ‘SCR D1’: frequency for ‘Subject information handed out’ and ‘Signed
 informed consent obtained from subject?’

Table A 5: Distribution of age and sex of all screened and randomized participants

Table A 6: Screening failures: Listing of screening number, sex, ethnicity, reason for no qualification to continuation with treatment phase

Table A 7: Demographic characteristics (‘SCR D1’): age and sex

Table A 8: Demographic characteristics (‘SCR D1’): ethnicity, listing of specification for other ethnicity

Table A 9: Type of Sport Regularly Performed (‘SCR D1’): number of training sessions/week, training
 time/week and previous training years for endurance

Table A 10: Type of Sport Regularly Performed (‘SCR D1’): number of training sessions/week, training
 time/week and previous training years for strength

Table A 11: Type of Sport Regularly Performed (‘SCR D1’): number of training sessions/week, training
 time/week and previous training years for other (if appropriate)

Table A 12: Type of Sport Regularly Performed (‘SCR D1’): frequency of ‘none’

Table A 13: Previous / concomitant disorders and therapies (past 3 months) (‘SCR D1’): listing

Table A 14: Inclusion criteria (part 1 + 2) (‘SCR D1’, ‘SCR D2’): frequencies of yes/no

Table A 15: Exclusion criteria (part 1 + 2) (‘SCR D1’, ‘SCR D2’): frequencies of yes/no

Table A 16: Anthropometry (‘SCR D1’): height

Table A 17: Anthropometry (‘SCR D1’): weight

Table A 18: Anthropometry (‘SCR D1’): BMI (calculated)

Table A 19: Vital signs (‘SCR D1’): blood pressure systolic

Table A 20: Vital signs (‘SCR D1’): blood pressure systolic (repeated if necessary (only listing))

Table A 21: Vital signs (‘SCR D1’): blood pressure diastolic

Table A 22: Vital signs (‘SCR D1’): blood pressure diastolic (repeated if necessary (only listing))

Table A 23: Vital signs (‘SCR D1’): heart rate

Table A 24: Vital signs (‘SCR D1’): heart rate (repeated if necessary (only listing))

Table A 25: Vital signs (‘SCR D1’): frequency for ‘Investigator’s decision: subject is qualified to

continue?’

Table A 26: Physical examination (‘SCR D1’): frequencies of normal and pathologic participants (head and neck, cardiovascular system, respiratory tract, abdomen, extremities and back, skin, neurological and psychiatric disorders, others), listing of specifications

Table A 27: Echocardiography (‘SCR D1’): frequency for ‘echocardiography done’ and reasons for ‘no’ (listing), sonographer initials (listing)

Table A 28: Echocardiography, only partial echocardiography (‘SCR D1’): frequency of the reasons, listing of problems with equipment, listing of other reasons

Table A 29: Echocardiography, picture quality (‘SCR D1’): frequency (parasternal short, parasternal long, apical)

Table A 30: Echocardiography, urgent findings (‘SCR D1’): frequency and listing of specification for others

Table A 31: Echocardiography, additional findings (‘SCR D1’): frequency and listing of specification for others

Table A 32: Echocardiography, left heart (‘SCR D1’): frequency for used axis (parasternal short axis, parasternal long axis)

Table A 33: Echocardiography, left heart parameters (‘SCR D1’): (AoB, LA, LA 4CHV, IVSd, LVIDd, LPWd, IVSs, LVIDs, LPWs, IVSd (a4K), LVEDD (a4K), Ao. asc.)

Table A 34: Echocardiography, left heart (‘SCR D1’): comments (listing)

Table A 35: Echocardiography, left ventricular ejection fraction (‘SCR D1’): frequency for visual assessment of LV-function

Table A 36: Echocardiography, EFSimpson (‘SCR D1’) (4 CHV, 2 CHV, biplan, LV EDV biplan, LV ESV biplan, GLS-2D)

Table A 37: Echocardiography, EFSimpson (‘SCR D1’): frequency for 3D-dataset acquired (yes/no)

Table A 38: Echocardiography, diastolic dysfunction (‘SCR D1’): frequency for grade

Table A 39: Echocardiography, diastolic dysfunction (‘SCR D1’): E, E’lat, A, E’med

Table A 40: Echocardiography, general regional kinetics: (‘SCR D1’) frequency (normal/abnormal)

Table A 41: Echocardiography, description of wall motion abnormalities (‘SCR D1’): listing

Table A 42: Echocardiography, further echocardiography data (‘SCR’): left ventricle: end-diastolic volume (ml)

Table A 43: Echocardiography, further echocardiography data (‘SCR’): left ventricle: end-systolic volume (ml)

Table A 44: Echocardiography, further echocardiography data (‘SCR’): left ventricle: ejection fraction (%)

Table A 45: Echocardiography, further echocardiography data (‘SCR’): left ventricle: endocardial global longitudinal strain (%)

Table A 46: Echocardiography, further echocardiography data (‘SCR’): left ventricle: ShapeFn (%)

Table A 47: Echocardiography, further echocardiography data (‘SCR’): left ventricle: myocardial global longitudinal strain (%)

Table A 48: Echocardiography, further echocardiography data (‘SCR’): left ventricle: global radial strain (%)

Table A 49: Echocardiography, further echocardiography data (‘SCR’): right ventricle: end-diastolic cross-sectional area (cm^2^)

Table A 50: Echocardiography, further echocardiography data (‘SCR’): right ventricle: end-systolic cross-sectional area (cm^2^)

Table A 51: Echocardiography, further echocardiography data (‘SCR’): right ventricle: fractional area change (%)

Table A 52: Echocardiography, further echocardiography data (‘SCR’): right ventricle: endocardial global longitudinal strain (%)

Table A 53: Echocardiography, further echocardiography data (‘SCR’): left atrium: end-diastolic volume (ml)

Table A 54: Echocardiography, further echocardiography data (‘SCR’): left atrium: end-systolic volume (ml)

Table A 55: Echocardiography, further echocardiography data (‘SCR’): left atrium: ejection fraction (%)

Table A 56: Echocardiography, further echocardiography data (‘SCR’): left atrium: endocardial global longitudinal strain (%)

Table A 57: Echocardiography, further echocardiography data (‘SCR’): left atrium: ShapeFn

Table A 58: Echocardiography, further echocardiography data (‘SCR’): left atrium: fractional area change (%)

Table A 59: Echocardiography, further echocardiography data (‘SCR’): right atrium: end-diastolic volume (ml)

Table A 60: Echocardiography, further echocardiography data (‘SCR’): right atrium: end-systolic volume (ml)

Table A 61: Echocardiography, further echocardiography data (‘SCR’): right atrium: ejection fraction (%)

Table A 62: Echocardiography, further echocardiography data (‘SCR’): right atrium: endocardial global longitudinal strain (%)

Table A 63: Echocardiography, further echocardiography data (‘SCR’): right atrium: ShapeFn

Table A 64: Echocardiography, further echocardiography data (‘SCR’): right atrium: fractional area change (%)

Table A 65: Echocardiography, further echocardiography data (‘SCR’): Listing of comments of echo data

Table A 66: ECG (‘SCR D1’): frequency of ‘Rhythm disorders at Screening Day 1 present?’, specification in case of yes (listing)

Table A 67: ECG (‘SCR D1’): frequency for ‘Study exclusion necessary?’ and ‘Subject qualified to continue?’ (yes/no)

Table A 68: ECG (‘SCR D1’): QTc

Table A 69: Blood samples (‘SCR D1’): frequency for EDTA plasma, reasons for no (listing)

Table A 70: Blood samples (‘SCR D1’): frequency for EDTA serum, reasons for no (listing)

Table A 71: Blood samples (‘SCR D1’): frequency for EDTA Lithium-Heparin, reasons for no (listing)

Table A 72: Blood samples (‘SCR D1’): frequency for EDTA citrate plasma, reasons for no (listing)

Table A 73: Urine sample (‘SCR D1’): frequency of ‘Urine (1 x 30 ml) given?’, reasons for no (listing)

Table A 74: Urine sample (‘SCR D1’): frequency of ‘Result of pregnancy test’ (for female participants)

Table A 75: Urine sample (‘SCR D1’): frequency of ‘Result of drug screening’

Table A 76: Respiratory Testing (‘SCR D1’), measurements and % predicted (FVC, FEV1, MEF25, TLC, FRC, RV, sRaw (sRaw: only the measurement, % predicted is not available)

Table A 77: Metacholine challenge test (‘SCR D1’): FEV1, change and frequencies for ‘Provocative dose 20% drop in FEV1 (PD20) reached?’ for Rest

Table A 78: Metacholine challenge test (‘SCR D1’): FEV1, change and frequencies for ‘Provocative dose 20% drop in FEV1 (PD20) reached?’ for Test 1

Table A 79: Metacholine challenge test (‘SCR D1’): FEV1, change and frequencies for ‘Provocative dose 20% drop in FEV1 (PD20) reached?’ for Test 2

Table A 80: Metacholine challenge test (‘SCR D1’): FEV1, change and frequencies for ‘Provocative dose 20% drop in FEV1 (PD20) reached?’ for Test 3

Table A 81: Metacholine challenge test (‘SCR D1’): FEV1, change and frequencies for ‘Provocative dose 20% drop in FEV1 (PD20) reached?’ for Test 4

Table A 82: Metacholine challenge test (‘SCR D1’): FEV1, change and frequencies for ‘Provocative dose 20% drop in FEV1 (PD20) reached?’ for Test 5

Table A 83: Metacholine challenge test (‘SCR D1’): frequency for diagnostic result

Table A 84: Cardio Pulmonary Exercise (CPX) (‘SCR D1’): frequency for protocols

Table A 85: Cardio Pulmonary Exercise (CPX), ergometer adjustment (‘SCR D1’) (Saddle position, saddle height, handlebar position, handlebar height)

Table A 86: Cardio Pulmonary Exercise (CPX), ergometer adjustment (‘SCR D1’): Pedal system & shoes (listing)

Table A 87: Cardio Pulmonary Exercise (CPX) (‘SCR D1’): Total time until exhaustion

Table A 88: Cardio Pulmonary Exercise (CPX) (‘SCR D1’): Peak power at VO_2max_ (incremental test)

Table A 89: Cardio Pulmonary Exercise (CPX) (‘SCR D1’): Preliminary VO_2max_ (incremental test)

Table A 90: Cardio Pulmonary Exercise (CPX) (‘SCR D1’): 110% of Peak Power (for verification test)

Table A 91: Cardio Pulmonary Exercise (CPX) (‘SCR D1’): Time until exhaustion (verification test)

Table A 92: Cardio Pulmonary Exercise (CPX) (‘SCR D1’): VO_2peak_ (l/min) and VO_2peak_ (ml/min/kg)

Table A 93: Cardio Pulmonary Exercise (CPX) (‘SCR D1’): ‘VO_2peak_ equals to Preliminary VO_2max_ ± 8.55%?’ (yes/no)

Table A 94: Repeated Cardio Pulmonary Exercise (CPX) (‘SCR D1’): Total time until exhaustion (only listing)

Table A 95: Repeated Cardio Pulmonary Exercise (CPX) (‘SCR D1’): Peak power at VO_2max_ (incremental test) (only listing)

Table A 96: Repeated Cardio Pulmonary Exercise (CPX) (‘SCR D1’): Preliminary VO_2max_ (incremental test) (only listing)

Table A 97: Repeated Cardio Pulmonary Exercise (CPX) (‘SCR D1’): 110% of Peak Power (for verification test) (only listing)

Table A 98: Repeated Cardio Pulmonary Exercise (CPX) (‘SCR D1’): Time until exhaustion (verification test) (only listing)

Table A 99: Repeated Cardio Pulmonary Exercise (CPX) (‘SCR D1’): VO_2peak_ (l/min) and VO_2peak_ (ml/min/kg) (only listing)

Table A 100: Repeated Cardio Pulmonary Exercise (CPX) (‘SCR D1’): ‘VO_2peak_ equals to Preliminary VO_2max_ ± 8.55%?’ (yes/no) (only listing)

Table A 101: Cardio Pulmonary Exercise (CPX) (‘SCR D1’): Verified VO_2max_ (l/min) and Verified VO_2max_ (ml/min/kg)

Table A 102: Cardio Pulmonary Exercise (CPX) (‘SCR D1’): VT at VO_2max_

Table A 103: Cardio Pulmonary Exercise (CPX) (‘SCR D1’): Heart Rate at VO_2max_

Table A 104: Cardio Pulmonary Exercise (CPX) (‘SCR D1’): Self-selected cadence during CPX test (Cad)

Table A 105: Test Time Trial (‘SCR D2’): frequency for ‘Ergometer adjustment identical to CPX test = yes’

Table A 106: Test Time Trial (‘SCR D2’): Power at 90% VO_2max_ (P90)

Table A 107: Test Time Trial (‘SCR D2’): Self-selected cadence during incremental test (Cad)

Table A 108: Test Time Trial (‘SCR D2’): Linear factor = P90 / Cad² x 10

Table A 109: Test Time Trial (‘SCR D2’): frequency for ‘Test completed’ (yes/no)

Table A 110: Test Time Trial (‘SCR D2’): duration of test in case of ‘Test completed=no’

Table A 111: Test Time Trial (‘SCR D2’): reasons for ‘Test completed=no’ (listing)

Table A 112: Test Time Trial (‘SCR D2’): frequency for ‘Adjustment of linear factor’ (yes/no) in case of ‘Test completed=no’

Table A 113: Test Time Trial (‘SCR D2’): new linear factor in case of ‘Adjustment of linear factor=no’ in case of ‘Test completed=no’

Table A 114: Test Time Trial (‘SCR D2’): Average Power Output

Table A 115: Test Time Trial (‘SCR D2’): Relative average Power Output

Table A 116: Cardiac Output (‘SCR D2’): Peak Power during time trial

Table A 117: Cardiac Output (‘SCR D2’): Peak-cardiac output

Table A 118: Cardiac Output (‘SCR D2’): Total mean power

Table A 119: Cardiac Output (‘SCR D2’): Heart rate

Table A 120: Cardiac Output (‘SCR D2’): Stroke volume

Table A 121: Cardiac Output (‘SCR D2’): O_2_

Table A 122: Cardiac Output (‘SCR D2’): Mean arterial pressure

Table A 123: Cardiac Output (‘SCR D2’): Total peripheral resistance

Table A 124: Cardiac Output (‘SCR D2’): SpO_2_

Table A 125: Post Exercise Blood Gas (‘SCR D2’): Lactate at 0, 5, 10 minutes

Table A 126: Post Exercise Blood Gas (‘SCR D2’): Glucose at 0, 5, 10 minutes

Table A 127: Post Exercise Blood Gas (‘SCR D2’): PO_2_ at 0, 5, 10 minutes

Table A 128: Post Exercise Blood Gas (‘SCR D2’): PCO_2_ at 0, 5, 10 minutes

Table A 129: Post Exercise Blood Gas (‘SCR D2’): pH at 0, 5, 10 minutes

Table A 130: Re-Check Inclusion- / Exclusion Criteria (‘SCR D2’): frequency of ‘All inclusion criteria met?’ (yes/no) and ‘No exclusion criteria given?’ (yes/no)

Table A 131: Screening Conclusion (‘SCR D2’): frequency ‘Participant qualified to continue with treatment phase?’ (yes/no)

Table A 132: Screening Conclusion (‘SCR D2’): frequencies for the specifications in case of qualification=no, specification for other reasons (listing)

Table A 133: Protocol violations (Listing of violations of inclusion-/ exclusion criteria, listing of

participants in whom the sealed envelope was opened in an emergency situation)

Table A 7 (only age), …, Table A 133: these tables will be provided for the whole study population only and in case of continuous variables by stratum male/female.

**Randomization, variables measured during the treatment phase (with exception of the study endpoints and safety variables), Study Conclusion**

Table B 1: Randomization to study arms for whole study population and by stratum male/female

Table B 2: Randomization to line 1, 2, 3, 4 for whole study population and by stratum male/female

Table B 3: Number of participants admitted to the study (distribution of participants across study arms and study treatments A, B, C, D)

Table B 4: Pre-study check before inhalation of study medication (‘A1D1’, ‘A2D1’, ‘A3D1’, ‘A4D1’): frequency for ‘Signed informed consent obtained from subject and still valid?’ (yes/no)

Table B 5: Pre-study check before inhalation of study medication (‘A1D1’, ‘A2D1’, ‘A3D1’, ‘A4D1’): frequency for ‘Did any changes in concomitant disease / treatment occur since last visit?’ (yes/no)

Table B 6: Pre-study check before inhalation of study medication (‘A1D1’, ‘A2D1’, ‘A3D1’, ‘A4D1’): frequency for ‘Did any Adverse Events occur since last visit?’ (yes/no)

Table B 7: Pre-study check before inhalation of study medication (‘A1D1’, ‘A2D1’, ‘A3D1’, ‘A4D1’): frequency for ‘Prior to each exercise testing: 24h: exhausting activity and alcohol consumed? 8h: fasting conditions? 2h: caffeine or caffeine-related products consumed?’ (yes/no)

Table B 8: Pre-study check before inhalation of study medication (‘A1D1’, ‘A2D1’, ‘A3D1’, ‘A4D1’): frequency for ‘Standardized meal given?’ (yes/no) for whole study population

Table B 9: Anthropometry (‘A1D1 before’, ‘A2D1 before’, ‘A3D1 before’, ‘A4D1 before’): body core temperature for whole study population and by stratum male/female

Table B 10: Anthropometry (‘A1D1 before’, ‘A2D1 before’, ‘A3D1 before’, ‘A4D1 before’): height for whole study population and by stratum male/female

Table B 11: Anthropometry (‘A1D1 before’, ‘A2D1 before’, ‘A3D1 before’, ‘A4D1 before’): weight for whole study population and by stratum male/female

Table B 12: Anthropometry (‘A1D1 before’, ‘A2D1 before’, ‘A3D1 before’, ‘A4D1 before’): BMI (calculated) for whole study population and by stratum male/female

Results for Vital Signs (blood pressure systolic, blood pressure diastolic, heart rate, ‘Investigator’s decision: subject is qualified to continue?’): see Table D1, …, D65 in section Safety below

Table B 13: Blood samples (‘D1 before’, ‘D1 15TT’, ‘D1 3hTT’, ‘D2’): frequency for EDTA plasma, Serum, Lithium-Heparin, reasons for no (listing)

Table B 14: Urine sample (‘D1 before’, ‘D1 15TT’, ‘D1 3hTT’, ‘D2’): frequency of ‘Urine (1 x 30 ml) given?’, reasons for no (listing)

Table B 15: Urine sample (‘A1D1 before’, ‘A2D1 before’, ‘A3D1 before’, ‘A4D1 before’): frequency of ‘Result of pregnancy test’ (N.A., Pos., Neg.)

Results for Respiratory Testing (FVC, FEV1, MEF25, TLC, FRC, RV sRaw): see Table C 79, …, C 156 in section Study endpoints below

Table B 16: Time Trial (‘A1D1 20after’, ‘A2D1 20after’, ‘A3D1 20after’, ‘A4D1 20after’): frequency for
‘Ergometer adjustment identical to CPX test = yes’

Table B 17: Time Trial (‘A1D1 20after’, ‘A2D1 20after’, ‘A3D1 20after’, ‘A4D1 20after’): Power at 90% VO_2max_ (P90)

Table B 18: Time Trial (‘A1D1 20after’, ‘A2D1 20after’, ‘A3D1 20after’, ‘A4D1 20after’): Self-selected cadence during incremental test (Cad)

Table B 19: Time Trial (‘A1D1 20after’, ‘A2D1 20after’, ‘A3D1 20after’, ‘A4D1 20after’): Linear factor = P90 / Cad² x 10

Table B 20: Time Trial (‘A1D1 20after’, ‘A2D1 20after’, ‘A3D1 20after’, ‘A4D1 20after’): frequency for ‘Test completed’ (yes/no)

Table B 21: Time Trial (‘A1D1 20after’, ‘A2D1 20after’, ‘A3D1 20after’, ‘A4D1 20after’): reasons for ‘Test completed=no’ (listing)

Table B 22: Time Trial (‘A1D1 20after’, ‘A2D1 20after’, ‘A3D1 20after’, ‘A4D1 20after’): duration of test in case of ‘Test completed=no’

Table B 23: Time Trial (‘A1D1 20after’, ‘A2D1 20after’, ‘A3D1 20after’, ‘A4D1 20after’): frequency for ‘Adjustment of linear factor’ (yes/no) in case of ‘Test completed=no’

Table B 24: Time Trial (‘A1D1 20after’, ‘A2D1 20after’, ‘A3D1 20after’, ‘A4D1 20after’): new linear factor in case of ‘Adjustment of linear factor=no’ in case of ‘Test completed=no’

Table B 25: Time Trial (‘A1D1 20after’, ‘A2D1 20after’, ‘A3D1 20after’, ‘A4D1 20after’): Average Power Output

Table B 26: Time Trial (‘A1D1 20after’, ‘A2D1 20after’, ‘A3D1 20after’, ‘A4D1 20after’): Relative average Power Output

Table B 27: Time Trial (‘A1D1 20after’, ‘A2D1 20after’, ‘A3D1 20after’, ‘A4D1 20after’): Peak Power during time trial

Table B 28: Cardiac Output (‘A1D1 20after’, ‘A2D1 20after’, ‘A3D1 20after’, ‘A4D1 20after’): Peak-cardiac output

Table B 29: Cardiac Output (‘A1D1 20after’, ‘A2D1 20after’, ‘A3D1 20after’, ‘A4D1 20after’): Total mean power

Table B 30: Cardiac Output (‘A1D1 20after’, ‘A2D1 20after’, ‘A3D1 20after’, ‘A4D1 20after’): Heart rate

Table B 31: Cardiac Output (‘A1D1 20after’, ‘A2D1 20after’, ‘A3D1 20after’, ‘A4D1 20after’): Stroke volume

Table B 32: Cardiac Output (‘A1D1 20after’, ‘A2D1 20after’, ‘A3D1 20after’, ‘A4D1 20after’): O_2_

Table B 33: Cardiac Output (‘A1D1 20after’, ‘A2D1 20after’, ‘A3D1 20after’, ‘A4D1 20after’): Mean arterial pressure

Table B 34: Cardiac Output (‘A1D1 20after’, ‘A2D1 20after’, ‘A3D1 20after’, ‘A4D1 20after’): Total peripheral resistance

Table B 35: Cardiac Output (‘A1D1 20after’, ‘A2D1 20after’, ‘A3D1 20after’, ‘A4D1 20after’): SpO_2_

Table B 36: Post Exercise Blood Gas (‘A1D1 20after’, ‘A2D1 20after’, ‘A3D1 20after’, ‘A4D1 20after’): Lactate at 0 minutes

Table B 37: Post Exercise Blood Gas (‘A1D1 20after’, ‘A2D1 20after’, ‘A3D1 20after’, ‘A4D1 20after’): Lactate at 5 minutes

Table B 38: Post Exercise Blood Gas (‘A1D1 20after’, ‘A2D1 20after’, ‘A3D1 20after’, ‘A4D1 20after’): Lactate at 10 minutes

Table B 39: Post Exercise Blood Gas (‘A1D1 20after’, ‘A2D1 20after’, ‘A3D1 20after’, ‘A4D1 20after’): Glucose at 0 minutes

Table B 40: Post Exercise Blood Gas (‘A1D1 20after’, ‘A2D1 20after’, ‘A3D1 20after’, ‘A4D1 20after’): Glucose at 5 minutes

Table B 41: Post Exercise Blood Gas (‘A1D1 20after’, ‘A2D1 20after’, ‘A3D1 20after’, ‘A4D1 20after’): Glucose at 10 minutes

Table B 42: Post Exercise Blood Gas (‘A1D1 20after’, ‘A2D1 20after’, ‘A3D1 20after’, ‘A4D1 20after’): PO2 at 0 minutes

Table B 43: Post Exercise Blood Gas (‘A1D1 20after’, ‘A2D1 20after’, ‘A3D1 20after’, ‘A4D1 20after’): PO_2_ at 5 minutes

Table B 44: Post Exercise Blood Gas (‘A1D1 20after’, ‘A2D1 20after’, ‘A3D1 20after’, ‘A4D1 20after’): PO_2_ at 10 minutes

Table B 45: Post Exercise Blood Gas (‘A1D1 20after’, ‘A2D1 20after’, ‘A3D1 20after’, ‘A4D1 20after’): PCO_2_ at 0 minutes

Table B 46: Post Exercise Blood Gas (‘A1D1 20after’, ‘A2D1 20after’, ‘A3D1 20after’, ‘A4D1 20after’): PCO_2_ at 5 minutes

Table B 47: Post Exercise Blood Gas (‘A1D1 20after’, ‘A2D1 20after’, ‘A3D1 20after’, ‘A4D1 20after’): PCO_2_ at 10 minutes

Table B 48: Post Exercise Blood Gas (‘A1D1 20after’, ‘A2D1 20after’, ‘A3D1 20after’, ‘A4D1 20after’): pH at 0 minutes

Table B 49: Post Exercise Blood Gas (‘A1D1 20after’, ‘A2D1 20after’, ‘A3D1 20after’, ‘A4D1 20after’): pH at 5 minutes

Table B 50: Post Exercise Blood Gas (‘A1D1 20after’, ‘A2D1 20after’, ‘A3D1 20after’, ‘A4D1 20after’): pH at 10 minutes

Table B 51: Echocardiography (‘A1D1 15TT’, ‘A2D1 15TT’, ‘A3D1 15TT’, ‘A4D1 15TT’): frequency for ‘echocardiography done’ (yes, yes partial, no) and reasons for ‘no’ (listing), sonographer initials (listing)

Table B 52: Echocardiography, only partial echocardiography (‘A1D1 15TT’, ‘A2D1 15TT’, ‘A3D1 15TT’, ‘A4D1 15TT’): frequency of the reasons, listing of problems, listing of other reasons

Table B 53: Echocardiography (‘A1D1 15TT’, ‘A2D1 15TT’, ‘A3D1 15TT’, ‘A4D1 15TT’): frequency of picture quality (parasternal short)

Table B 54: Echocardiography (‘A1D1 15TT’, ‘A2D1 15TT’, ‘A3D1 15TT’, ‘A4D1 15TT’): frequency of picture quality (parasternal long)

Table B 55: Echocardiography (‘A1D1 15TT’, ‘A2D1 15TT’, ‘A3D1 15TT’, ‘A4D1 15TT’): frequency of picture quality (apical)

Table B 56: Echocardiography, urgent findings (‘A1D1 15TT’, ‘A2D1 15TT’, ‘A3D1 15TT’, ‘A4D1 15TT’): frequency and listing of specification for others

Table B 57: Echocardiography, additional findings (‘A1D1 15TT’, ‘A2D1 15TT’, ‘A3D1 15TT’, ‘A4D1 15TT’): frequency and listing of specification for others

Table B 58: Echocardiography, left heart (‘A1D1 15TT’, ‘A2D1 15TT’, ‘A3D1 15TT’, ‘A4D1 15TT’):
 frequency for used axis (parasternal short axis, parasternal long axis)

Table B 59: Echocardiography, left heart (‘A1D1 15TT’, ‘A2D1 15TT’, ‘A3D1 15TT’, ‘A4D1 15TT’): AoB

Table B 60: Echocardiography, left heart (‘A1D1 15TT’, ‘A2D1 15TT’, ‘A3D1 15TT’, ‘A4D1 15TT’): LA

Table B 61: Echocardiography, left heart (‘A1D1 15TT’, ‘A2D1 15TT’, ‘A3D1 15TT’, ‘A4D1 15TT’):
LA 4CHV

Table B 62: Echocardiography, left heart (‘A1D1 15TT’, ‘A2D1 15TT’, ‘A3D1 15TT’, ‘A4D1 15TT’):
 IVSd

Table B 63: Echocardiography, left heart (‘A1D1 15TT’, ‘A2D1 15TT’, ‘A3D1 15TT’, ‘A4D1 15TT’):
 LVIDd

Table B 64: Echocardiography, left heart (‘A1D1 15TT’, ‘A2D1 15TT’, ‘A3D1 15TT’, ‘A4D1 15TT’):
 LPWd

Table B 65: Echocardiography, left heart (‘A1D1 15TT’, ‘A2D1 15TT’, ‘A3D1 15TT’, ‘A4D1 15TT’):
 IVSs

Table B 66: Echocardiography, left heart (‘A1D1 15TT’, ‘A2D1 15TT’, ‘A3D1 15TT’, ‘A4D1 15TT’): LVIDs

Table B 67: Echocardiography, left heart (‘A1D1 15TT’, ‘A2D1 15TT’, ‘A3D1 15TT’, ‘A4D1 15TT’): LPWs

Table B 68: Echocardiography, left heart (‘A1D1 15TT’, ‘A2D1 15TT’, ‘A3D1 15TT’, ‘A4D1 15TT’): IVSd (a4K)

Table B 69: Echocardiography, left heart (‘A1D1 15TT’, ‘A2D1 15TT’, ‘A3D1 15TT’, ‘A4D1 15TT’): LVEDD (a4K)

Table B 70: Echocardiography, left heart (‘A1D1 15TT’, ‘A2D1 15TT’, ‘A3D1 15TT’, ‘A4D1 15TT’):
Ao. asc.

Table B 71: Echocardiography, left heart (‘A1D1 15TT’, ‘A2D1 15TT’, ‘A3D1 15TT’, ‘A4D1 15TT’): comments (listing)

Table B 72: Echocardiography, left ventricular ejection fraction (‘A1D1 15TT’, ‘A2D1 15TT’, ‘A3D1 15TT’, ‘A4D1 15TT’): frequency for visual assessment of LV-function

Table B 73: Echocardiography, EFSimpson (‘A1D1 15TT’, ‘A2D1 15TT’, ‘A3D1 15TT’, ‘A4D1 15TT’):
4 CHV

Table B 74: Echocardiography, EFSimpson (‘A1D1 15TT’, ‘A2D1 15TT’, ‘A3D1 15TT’, ‘A4D1 15TT’):
2 CHV

Table B 75: Echocardiography, EFSimpson (‘A1D1 15TT’, ‘A2D1 15TT’, ‘A3D1 15TT’, ‘A4D1 15TT’): biplan

Table B 76: Echocardiography, EFSimpson (‘A1D1 15TT’, ‘A2D1 15TT’, ‘A3D1 15TT’, ‘A4D1 15TT’): LV EDV biplan

Table B 77: Echocardiography, EFSimpson (‘A1D1 15TT’, ‘A2D1 15TT’, ‘A3D1 15TT’, ‘A4D1 15TT’): LV ESV biplan

Table B 78: Echocardiography, EFSimpson (‘A1D1 15TT’, ‘A2D1 15TT’, ‘A3D1 15TT’, ‘A4D1 15TT’): GLS-2D

Table B 79: Echocardiography, EFSimpson (‘A1D1 15TT’, ‘A2D1 15TT’, ‘A3D1 15TT’, ‘A4D1 15TT’): frequency for 3D-dataset acquired (yes/no)

Table B 80: Echocardiography, diastolic dysfunction (‘A1D1 15TT’, ‘A2D1 15TT’, ‘A3D1 15TT’, ‘A4D1 15TT’): frequency for grade

Table B 81: Echocardiography, diastolic dysfunction (‘A1D1 15TT’, ‘A2D1 15TT’, ‘A3D1 15TT’, ‘A4D1 15TT’): E

Table B 82: Echocardiography, diastolic dysfunction (‘A1D1 15TT’, ‘A2D1 15TT’, ‘A3D1 15TT’, ‘A4D1 15TT’): E’lat

Table B 83: Echocardiography, diastolic dysfunction (‘A1D1 15TT’, ‘A2D1 15TT’, ‘A3D1 15TT’, ‘A4D1 15TT’): A

Table B 84: Echocardiography, diastolic dysfunction (‘A1D1 15TT’, ‘A2D1 15TT’, ‘A3D1 15TT’, ‘A4D1 15TT’): E’med

Table B 85: Echocardiography, general regional kinetics: (‘A1D1 15TT’, ‘A2D1 15TT’, ‘A3D1 15TT’, ‘A4D1 15TT’) frequency (normal/abnormal)

Table B 86: Echocardiography, wall motion abnormalities (‘A1D1 15TT’, ‘A2D1 15TT’, ‘A3D1 15TT’, ‘A4D1 15TT’): listing

Table B 87: Echocardiography, further echocardiography data (‘A1D1’, ‘A2D1’, ‘A3D1’, ‘A4D1’):
left ventricle: end-diastolic volume (ml)

Table B 88: Echocardiography, further echocardiography data (‘A1D1’, ‘A2D1’, ‘A3D1’, ‘A4D1’):
left ventricle: end-systolic volume (ml)

Table B 89: Echocardiography, further echocardiography data (‘A1D1’, ‘A2D1’, ‘A3D1’, ‘A4D1’):
left ventricle: ejection fraction (%)

Table B 90: Echocardiography, further echocardiography data (‘A1D1’, ‘A2D1’, ‘A3D1’, ‘A4D1’):
left ventricle: endocardial global longitudinal strain (%)

Table B 91: Echocardiography, further echocardiography data (‘A1D1’, ‘A2D1’, ‘A3D1’, ‘A4D1’):
left ventricle: ShapeFn (%)

Table B 92: Echocardiography, further echocardiography data (‘A1D1’, ‘A2D1’, ‘A3D1’, ‘A4D1’):
left ventricle: myocardial global longitudinal strain (%)

Table B 93: Echocardiography, further echocardiography data (‘A1D1’, ‘A2D1’, ‘A3D1’, ‘A4D1’):
left ventricle: global radial strain (%)

Table B 94: Echocardiography, further echocardiography data (‘A1D1’, ‘A2D1’, ‘A3D1’, ‘A4D1’):
right ventricle: end-diastolic cross-sectional area (cm^2^)

Table B 95: Echocardiography, further echocardiography data (‘A1D1’, ‘A2D1’, ‘A3D1’, ‘A4D1’):
right ventricle: end-systolic cross-sectional area (cm^2^)

Table B 96: Echocardiography, further echocardiography data (‘A1D1’, ‘A2D1’, ‘A3D1’, ‘A4D1’):
right ventricle: fractional area change (%)

Table B 97: Echocardiography, further echocardiography data (‘A1D1’, ‘A2D1’, ‘A3D1’, ‘A4D1’):
right ventricle: endocardial global longitudinal strain (%)

Table B 98: Echocardiography, further echocardiography data (‘A1D1’, ‘A2D1’, ‘A3D1’, ‘A4D1’):
left atrium: end-diastolic volume (ml)

Table B 99: Echocardiography, further echocardiography data (‘A1D1’, ‘A2D1’, ‘A3D1’, ‘A4D1’):
left atrium: end-systolic volume (ml)

Table B 100: Echocardiography, further echocardiography data (‘A1D1’, ‘A2D1’, ‘A3D1’, ‘A4D1’):
left atrium: ejection fraction (%)

Table B 101: Echocardiography, further echocardiography data (‘A1D1’, ‘A2D1’, ‘A3D1’, ‘A4D1’):
left atrium: endocardial global longitudinal strain (%)

Table B 102: Echocardiography, further echocardiography data (‘A1D1’, ‘A2D1’, ‘A3D1’, ‘A4D1’):
left atrium: ShapeFn

Table B 103: Echocardiography, further echocardiography data (‘A1D1’, ‘A2D1’, ‘A3D1’, ‘A4D1’):
left atrium: fractional area change (%)

Table B 104: Echocardiography, further echocardiography data (‘A1D1’, ‘A2D1’, ‘A3D1’, ‘A4D1’):
right atrium: end-diastolic volume (ml)

Table B 105: Echocardiography, further echocardiography data (‘A1D1’, ‘A2D1’, ‘A3D1’, ‘A4D1’):
right atrium: end-systolic volume (ml)

Table B 106: Echocardiography, further echocardiography data (‘A1D1’, ‘A2D1’, ‘A3D1’, ‘A4D1’):
right atrium: ejection fraction (%)

Table B 107: Echocardiography, further echocardiography data (‘A1D1’, ‘A2D1’, ‘A3D1’, ‘A4D1’):
right atrium: endocardial global longitudinal strain (%)

Table B 108: Echocardiography, further echocardiography data (‘A1D1’, ‘A2D1’, ‘A3D1’, ‘A4D1’):
right atrium: ShapeFn

Table B 109: Echocardiography, further echocardiography data (‘A1D1’, ‘A2D1’, ‘A3D1’, ‘A4D1’):
right atrium: fractional area change (%)

Table B 110: Echocardiography, further echocardiography data (‘A1D1’, ‘A2D1’, ‘A3D1’, ‘A4D1’):
Listing of comments of echo data

Table B 111: Muscle biopsy (‘A1D1 3hTT’, ‘A2D1 3hTT’, ‘A3D1 3hTT’, ‘A4D1 3hTT’): frequency for ‘Muscle biopsy of m. vastus lateralis taken from’ (right leg/left leg)

Table B 112: Muscle biopsy (‘A1D1 3hTT’, ‘A2D1 3hTT’, ‘A3D1 3hTT’, ‘A4D1 3hTT’): frequency for ‘Did the muscle biopsy occur according to protocol?’ (yes/no)

Table B 113: Side effects (‘A1D1’, ‘A2D1’, ‘A3D1’, ‘A4D1’): frequency for ‘Did any side effects occur during day 1?’ (yes/no)

Table B 114: Change in concomitant disorder / medications (‘A1D2’, ‘A2D2’, ‘A3D2’, ‘A4D2’): frequency for ‘Did any changes in concomitant disorders / medications occur since last visit?’ (yes/no)

Table B 115: Use of self-medication (‘A1D2’, ‘A2D2’, ‘A3D2’, ‘A4D2’): frequency for ‘Did subject use any self-medication for post-exercise conditions?’ (yes/no) and ‘prescribed?’ (yes/no), name of prescribing doctor (listing)

Table B 116: Did any side effects occur since last visit?

Table B 117: Telephone call (‘A1D34’, ‘A2D34’, ‘A3D34’, ‘A4D34’): frequency for ‘Did any side effects occur since last visit?’ (yes/no)

Table B 118: Study conclusion: did subject complete the study as planned?

Table B 119: Study conclusion: reasons if subject not completed the study as planned
 (listing of specification for other reason).

Table B 120: Listing of remarks

Table B 16, …, Table B 120 are for whole study population and by treatment A, B, C, D

Results for ECG: see Table C 157, …, C 174 in section Study endpoints below

**Study endpoints**

**Muscular expression of nuclear receptors (NR4A 1/2/3):**

Table C 1: Muscular expression of nuclear receptors (‘A1D1 3hTT’, ‘A2D1 3hTT’, ‘A3D1 3hTT’, ‘A4D1 3hTT’): p.11 for whole study population

Table C 2: Muscular expression of nuclear receptors (‘A1D1 3hTT’, ‘A2D1 3hTT’, ‘A3D1 3hTT’, ‘A4D1 3hTT’): p.11 for stratum male

Table C 3: Muscular expression of nuclear receptors (‘A1D1 3hTT’, ‘A2D1 3hTT’, ‘A3D1 3hTT’, ‘A4D1 3hTT’): p.11 for stratum female

Table C 4: Muscular expression of nuclear receptors (‘A1D1 3hTT’, ‘A2D1 3hTT’, ‘A3D1 3hTT’, ‘A4D1 3hTT’): p.11 for whole study population by treatment A, B, C, D

Table C 5: Muscular expression of nuclear receptors (‘A1D1 3hTT’, ‘A2D1 3hTT’, ‘A3D1 3hTT’, ‘A4D1 3hTT’): p.11 for stratum male by treatment A, B, C, D

Table C 6: Muscular expression of nuclear receptors (‘A1D1 3hTT’, ‘A2D1 3hTT’, ‘A3D1 3hTT’, ‘A4D1 3hTT’): p.11 for stratum female by treatment A, B, C, D

Analogue tables will be created for all other parameter p.1x of muscular expression of nuclear receptors.

**Hormone and cytokine levels and damage markers:**

Table C 7: Hormone and cytokine levels and damage markers (‘A1D1 before’, ‘A1D1 15TT’, ‘A1D1 3hTT’, ‘A1D2’): p.21 for whole study population

Table C 8: Hormone and cytokine levels and damage markers (‘A2D1 before’, ‘A2D1 15TT’, ‘A2D1 3hTT’, ‘A2D2’): p.21 for whole study population

Table C 9: Hormone and cytokine levels and damage markers (‘A3D1 before’, ‘A3D1 15TT’, ‘A3D1 3hTT’, ‘A3D2’): p.21 for whole study population

Table C 10: Hormone and cytokine levels and damage markers (‘A4D1 before’, ‘A4D1 15TT’, ‘A4D1 3hTT’, ‘A4D2’): p.21 for whole study population

Table C 11: Hormone and cytokine levels and damage markers (‘A1D1 before’, ‘A1D1 15TT’, ‘A1D1 3hTT’, ‘A1D2’): p.21 for stratum male

Table C 12: Hormone and cytokine levels and damage markers (‘A2D1 before’, ‘A2D1 15TT’, ‘A2D1 3hTT’, ‘A2D2’): p.21 for stratum male

Table C 13: Hormone and cytokine levels and damage markers (‘A3D1 before’, ‘A3D1 15TT’, ‘A3D1 3hTT’, ‘A3D2’): p.21 for stratum male

Table C 14: Hormone and cytokine levels and damage markers (‘A4D1 before’, ‘A4D1 15TT’, ‘A4D1 3hTT’, ‘A4D2’): p.21 for stratum male

Table C 15: Hormone and cytokine levels and damage markers (‘A1D1 before’, ‘A1D1 15TT’, ‘A1D1 3hTT’, ‘A1D2’): p.21 for stratum female

Table C 16: Hormone and cytokine levels and damage markers (‘A2D1 before’, ‘A2D1 15TT’, ‘A2D1 3hTT’, ‘A2D2’): p.21 for stratum female

Table C 17: Hormone and cytokine levels and damage markers (‘A3D1 before’, ‘A3D1 15TT’, ‘A3D1 3hTT’, ‘A3D2’): p.21 for stratum female

Table C 18: Hormone and cytokine levels and damage markers (‘A4D1 before’, ‘A4D1 15TT’, ‘A4D1 3hTT’, ‘A4D2’): p.21 for stratum female

Table C 19: Hormone and cytokine levels and damage markers (‘A1D1 before’, ‘A1D1 15TT’, ‘A1D1 3hTT’, ‘A1D2’): p.21 for whole study population by treatment A, B, C, D

Table C 20: Hormone and cytokine levels and damage markers (‘A2D1 before’, ‘A2D1 15TT’, ‘A2D1 3hTT’, ‘A2D2’): p.21 for whole study population by treatment A, B, C, D

Table C 21: Hormone and cytokine levels and damage markers (‘A3D1 before’, ‘A3D1 15TT’, ‘A3D1 3hTT’, ‘A3D2’): p.21 for whole study population by treatment A, B, C, D

Table C 22: Hormone and cytokine levels and damage markers (‘A4D1 before’, ‘A4D1 15TT’, ‘A4D1 3hTT’, ‘A4D2’): p.21 for whole study population by treatment A, B, C, D

Table C 23: Hormone and cytokine levels and damage markers (‘A1D1 before’, ‘A1D1 15TT’, ‘A1D1 3hTT’, ‘A1D2’): p.21 for stratum male by treatment A, B, C, D

Table C 24: Hormone and cytokine levels and damage markers (‘A2D1 before’, ‘A2D1 15TT’, ‘A2D1 3hTT’, ‘A2D2’): p.21 for stratum male by treatment A, B, C, D

Table C 25: Hormone and cytokine levels and damage markers (‘A3D1 before’, ‘A3D1 15TT’, ‘A3D1 3hTT’, ‘A3D2’): p.21 for stratum male by treatment A, B, C, D

Table C 26: Hormone and cytokine levels and damage markers (‘A4D1 before’, ‘A4D1 15TT’, ‘A4D1 3hTT’, ‘A4D2’): p.21 for stratum male by treatment A, B, C, D

Table C 27: Hormone and cytokine levels and damage markers (‘A1D1 before’, ‘A1D1 15TT’, ‘A1D1 3hTT’, ‘A1D2’): p.21 for stratum female by treatment A, B, C, D

Table C 28: Hormone and cytokine levels and damage markers (‘A2D1 before’, ‘A2D1 15TT’, ‘A2D1 3hTT’, ‘A2D2’): p.21 for stratum female by treatment A, B, C, D

Table C 29: Hormone and cytokine levels and damage markers (‘A3D1 before’, ‘A3D1 15TT’, ‘A3D1 3hTT’, ‘A3D2’): p.21 for stratum female by treatment A, B, C, D

Table C 30: Hormone and cytokine levels and damage markers (‘A4D1 before’, ‘A4D1 15TT’, ‘A4D1 3hTT’, ‘A4D2’): p.21 for stratum female by treatment A, B, C, D

Analogue tables will be created for the following differences in the parameter p.21: ‘AxD1 15TT’ minus ‘AxD1 before’, ‘AxD1 3hTT’ minus ‘AxD1 before’, ‘AxD2’ minus ‘AxD1 before’, ‘AxD1 3hTT’ minus ‘AxD1 15TT’, ‘AxD2’ minus ‘AxD1 15TT’, ‘AxD2’ minus ‘AxD1 3hTT’, x labels the study arm, x=1, 2, 3, 4.

Analogue tables will be created for all other parameter p.2x of hormone and cytokine levels and damage markers and all possible differences in p.2x.

**Urine and plasma concentrations of salbutamol and formoterol:**

Table C 31: Urine and plasma concentrations of salbutamol and formoterol (‘A1D1 before’, ‘A1D1 15TT’, ‘A1D1 3hTT’, ‘A1D2’): p.31 for whole study population

Table C 32: Urine and plasma concentrations of salbutamol and formoterol (‘A2D1 before’, ‘A2D1 15TT’, ‘A2D1 3hTT’, ‘A2D2’): p.31 for whole study population

Table C 33: Urine and plasma concentrations of salbutamol and formoterol (‘A2D1 before’, ‘A2D1 15TT’, ‘A2D1 3hTT’, ‘A2D2’): p.31 for whole study population

Table C 34: Urine and plasma concentrations of salbutamol and formoterol (‘A3D1 before’, ‘A3D1 15TT’, ‘A3D1 3hTT’, ‘A3D2’): p.31 for whole study population

Table C 35: Urine and plasma concentrations of salbutamol and formoterol (‘A1D1 before’, ‘A1D1 15TT’, ‘A1D1 3hTT’, ‘A1D2’): p.31 for stratum male

Table C 36: Urine and plasma concentrations of salbutamol and formoterol (‘A2D1 before’, ‘A2D1 15TT’, ‘A2D1 3hTT’, ‘A2D2’): p.31 for stratum male

Table C 37: Urine and plasma concentrations of salbutamol and formoterol (‘A2D1 before’, ‘A2D1 15TT’, ‘A2D1 3hTT’, ‘A2D2’): p.31 for stratum male

Table C 38: Urine and plasma concentrations of salbutamol and formoterol (‘A3D1 before’, ‘A3D1 15TT’, ‘A3D1 3hTT’, ‘A3D2’): p.31 for stratum male

Table C 39: Urine and plasma concentrations of salbutamol and formoterol (‘A1D1 before’, ‘A1D1 15TT’, ‘A1D1 3hTT’, ‘A1D2’): p.31 for stratum female

Table C 40: Urine and plasma concentrations of salbutamol and formoterol (‘A2D1 before’, ‘A2D1 15TT’, ‘A2D1 3hTT’, ‘A2D2’): p.31 for stratum female

Table C 41: Urine and plasma concentrations of salbutamol and formoterol (‘A2D1 before’, ‘A2D1 15TT’, ‘A2D1 3hTT’, ‘A2D2’): p.31 for stratum female

Table C 42: Urine and plasma concentrations of salbutamol and formoterol (‘A3D1 before’, ‘A3D1 15TT’, ‘A3D1 3hTT’, ‘A3D2’): p.31 for stratum female

Table C 43: Urine and plasma concentrations of salbutamol and formoterol (‘A1D1 before’, ‘A1D1 15TT’, ‘A1D1 3hTT’, ‘A1D2’): p.31 for whole study population by treatment A, B, C, D

Table C 44: Urine and plasma concentrations of salbutamol and formoterol (‘A2D1 before’, ‘A2D1 15TT’, ‘A2D1 3hTT’, ‘A2D2’): p.31 for whole study population by treatment A, B, C, D

Table C 45: Urine and plasma concentrations of salbutamol and formoterol (‘A2D1 before’, ‘A2D1 15TT’, ‘A2D1 3hTT’, ‘A2D2’): p.31 for whole study population by treatment A, B, C, D

Table C 46: Urine and plasma concentrations of salbutamol and formoterol (‘A3D1 before’, ‘A3D1 15TT’, ‘A3D1 3hTT’, ‘A3D2’): p.31 for whole study population by treatment A, B, C, D

Table C 47: Urine and plasma concentrations of salbutamol and formoterol (‘A1D1 before’, ‘A1D1 15TT’, ‘A1D1 3hTT’, ‘A1D2’): p.31 for stratum male by treatment A, B, C, D

Table C 48: Urine and plasma concentrations of salbutamol and formoterol (‘A2D1 before’, ‘A2D1 15TT’, ‘A2D1 3hTT’, ‘A2D2’): p.31 for stratum male by treatment A, B, C, D

Table C 49: Urine and plasma concentrations of salbutamol and formoterol (‘A2D1 before’, ‘A2D1 15TT’, ‘A2D1 3hTT’, ‘A2D2’): p.31 for stratum male by treatment A, B, C, D

Table C 50: Urine and plasma concentrations of salbutamol and formoterol (‘A3D1 before’, ‘A3D1 15TT’, ‘A3D1 3hTT’, ‘A3D2’): p.31 for stratum male by treatment A, B, C, D

Table C 51: Urine and plasma concentrations of salbutamol and formoterol (‘A1D1 before’, ‘A1D1 15TT’, ‘A1D1 3hTT’, ‘A1D2’): p.31 for stratum female by treatment A, B, C, D

Table C 52: Urine and plasma concentrations of salbutamol and formoterol (‘A2D1 before’, ‘A2D1 15TT’, ‘A2D1 3hTT’, ‘A2D2’): p.31 for stratum female by treatment A, B, C, D

Table C 53: Urine and plasma concentrations of salbutamol and formoterol (‘A2D1 before’, ‘A2D1 15TT’, ‘A2D1 3hTT’, ‘A2D2’): p.31 for stratum female by treatment A, B, C, D

Table C 54: Urine and plasma concentrations of salbutamol and formoterol (‘A3D1 before’, ‘A3D1 15TT’, ‘A3D1 3hTT’, ‘A3D2’): p.31 for stratum female by treatment A, B, C, D

Analogue tables will be created for the following differences in the parameter p.31: ‘AxD1 15TT’ minus ‘AxD1 before’, ‘AxD1 3hTT’ minus ‘AxD1 before’, ‘AxD2’ minus ‘AxD1 before’, ‘AxD1 3hTT’ minus ‘AxD1 15TT’, ‘AxD2’ minus ‘AxD1 15TT’, ‘AxD2’ minus ‘AxD1 3hTT’, x labels the study arm, x=1, 2, 3, 4.

Analogue tables will be created for all other parameter p.3x of urine and plasma concentrations of salbutamol and formoterol and all possible differences in p.3x.

**Circulating cardiac markers:**

Table C 55: Circulating cardiac markers (‘A1D1 before’, ‘A1D1 15TT’, ‘A1D1 3hTT’, ‘A1D2’): p.41 for whole study population

Table C 56: Circulating cardiac markers (‘A2D1 before’, ‘A2D1 15TT’, ‘A2D1 3hTT’, ‘A2D2’): p.41 for whole study population

Table C 57: Circulating cardiac markers (‘A3D1 before’, ‘A3D1 15TT’, ‘A3D1 3hTT’, ‘A3D2’): p.41 for whole study population

Table C 58: Circulating cardiac markers (‘A4D1 before’, ‘A4D1 15TT’, ‘A4D1 3hTT’, ‘A4D2’): p.41 for whole study population

Table C 59: Circulating cardiac markers (‘A1D1 before’, ‘A1D1 15TT’, ‘A1D1 3hTT’, ‘A1D2’): p.41 for stratum male

Table C 60: Circulating cardiac markers (‘A2D1 before’, ‘A2D1 15TT’, ‘A2D1 3hTT’, ‘A2D2’): p.41 for stratum male

Table C 61: Circulating cardiac markers (‘A3D1 before’, ‘A3D1 15TT’, ‘A3D1 3hTT’, ‘A3D2’): p.41 for stratum male

Table C 62: Circulating cardiac markers (‘A4D1 before’, ‘A4D1 15TT’, ‘A4D1 3hTT’, ‘A4D2’): p.41 for stratum male

Table C 63: Circulating cardiac markers (‘A1D1 before’, ‘A1D1 15TT’, ‘A1D1 3hTT’, ‘A1D2’): p.41 for stratum female

Table C 64: Circulating cardiac markers (‘A2D1 before’, ‘A2D1 15TT’, ‘A2D1 3hTT’, ‘A2D2’): p.41 for stratum female

Table C 65: Circulating cardiac markers (‘A3D1 before’, ‘A3D1 15TT’, ‘A3D1 3hTT’, ‘A3D2’): p.41 for stratum female

Table C 66: Circulating cardiac markers (‘A4D1 before’, ‘A4D1 15TT’, ‘A4D1 3hTT’, ‘A4D2’): p.41 for stratum female

Table C 67: Circulating cardiac markers (‘A1D1 before’, ‘A1D1 15TT’, ‘A1D1 3hTT’, ‘A1D2’): p.41 for whole study population by treatment A, B, C, D

Table C 68: Circulating cardiac markers (‘A2D1 before’, ‘A2D1 15TT’, ‘A2D1 3hTT’, ‘A2D2’): p.41 for whole study population by treatment A, B, C, D

Table C 69: Circulating cardiac markers (‘A3D1 before’, ‘A3D1 15TT’, ‘A3D1 3hTT’, ‘A3D2’): p.41 for whole study population by treatment A, B, C, D

Table C 70: Circulating cardiac markers (‘A4D1 before’, ‘A4D1 15TT’, ‘A4D1 3hTT’, ‘A4D2’): p.41 for whole study population by treatment A, B, C, D

Table C 71: Circulating cardiac markers (‘A1D1 before’, ‘A1D1 15TT’, ‘A1D1 3hTT’, ‘A1D2’): p.41 for stratum male by treatment A, B, C, D

Table C 72: Circulating cardiac markers (‘A2D1 before’, ‘A2D1 15TT’, ‘A2D1 3hTT’, ‘A2D2’): p.41 for stratum male by treatment A, B, C, D

Table C 73: Circulating cardiac markers (‘A3D1 before’, ‘A3D1 15TT’, ‘A3D1 3hTT’, ‘A3D2’): p.41 for stratum male by treatment A, B, C, D

Table C 74: Circulating cardiac markers (‘A4D1 before’, ‘A4D1 15TT’, ‘A4D1 3hTT’, ‘A4D2’): p.41 for stratum male by treatment A, B, C, D

Table C 75: Circulating cardiac markers (‘A1D1 before’, ‘A1D1 15TT’, ‘A1D1 3hTT’, ‘A1D2’): p.41 for stratum female by treatment A, B, C, D

Table C 76: Circulating cardiac markers (‘A2D1 before’, ‘A2D1 15TT’, ‘A2D1 3hTT’, ‘A2D2’): p.41 for stratum female by treatment A, B, C, D

Table C 77: Circulating cardiac markers (‘A3D1 before’, ‘A3D1 15TT’, ‘A3D1 3hTT’, ‘A3D2’): p.41 for stratum female by treatment A, B, C, D

Table C 78: Circulating cardiac markers (‘A4D1 before’, ‘A4D1 15TT’, ‘A4D1 3hTT’, ‘A4D2’): p.41 for stratum female by treatment A, B, C, D

Analogue tables will be created for the following differences in the parameter p.41: ‘AxD1 15TT’ minus ‘AxD1 before’, ‘AxD1 3hTT’ minus ‘AxD1 before’, ‘AxD2’ minus ‘AxD1 before’, ‘AxD1 3hTT’ minus ‘AxD1 15TT’, ‘AxD2’ minus ‘AxD1 15TT’, ‘AxD2’ minus ‘AxD1 3hTT’, x labels the study arm, x=1, 2, 3, 4.

Analogue tables will be created for all other parameter p.4x of circulating cardiac markers and all possible differences in p.4x.

**Cardiopulmonary function (Respiratory testing):**

Table C 79: Respiratory Testing (‘A1D1 before’, ‘A1D1 15TT’, ‘A1D2’, ‘A2D1 before’, ‘A2D1 15TT’, ‘A2D2’, ‘A3D1 before’, ‘A3D1 15TT’, ‘A3D2’, ‘A4D1 before’, ‘A4D1 15TT’, ‘A4D2’): FVC (measured value) for whole study population

Table C 80: Respiratory Testing (‘A1D1 before’, ‘A1D1 15TT’, ‘A1D2’, ‘A2D1 before’, ‘A2D1 15TT’, ‘A2D2’, ‘A3D1 before’, ‘A3D1 15TT’, ‘A3D2’, ‘A4D1 before’, ‘A4D1 15TT’, ‘A4D2’): FVC (measured value) for stratum male

Table C 81: Respiratory Testing (‘A1D1 before’, ‘A1D1 15TT’, ‘A1D2’, ‘A2D1 before’, ‘A2D1 15TT’, ‘A2D2’, ‘A3D1 before’, ‘A3D1 15TT’, ‘A3D2’, ‘A4D1 before’, ‘A4D1 15TT’, ‘A4D2’): FVC (measured value) for stratum female

Table C 82: Respiratory Testing (‘A1D1 before’, ‘A1D1 15TT’, ‘A1D2’, ‘A2D1 before’, ‘A2D1 15TT’, ‘A2D2’, ‘A3D1 before’, ‘A3D1 15TT’, ‘A3D2’, ‘A4D1 before’, ‘A4D1 15TT’, ‘A4D2’): FVC (measured value) for whole study population by treatment A, B, C, D

Table C 83: Respiratory Testing (‘A1D1 before’, ‘A1D1 15TT’, ‘A1D2’, ‘A2D1 before’, ‘A2D1 15TT’, ‘A2D2’, ‘A3D1 before’, ‘A3D1 15TT’, ‘A3D2’, ‘A4D1 before’, ‘A4D1 15TT’, ‘A4D2’): FVC (measured value) for stratum male by treatment A, B, C, D

Table C 84: Respiratory Testing (‘A1D1 before’, ‘A1D1 15TT’, ‘A1D2’, ‘A2D1 before’, ‘A2D1 15TT’, ‘A2D2’, ‘A3D1 before’, ‘A3D1 15TT’, ‘A3D2’, ‘A4D1 before’, ‘A4D1 15TT’, ‘A4D2’): FVC (measured value) for stratum female by treatment A, B, C, D

Table C 85: Respiratory Testing (‘A1D1 before’, ‘A1D1 15TT’, ‘A1D2’, ‘A2D1 before’, ‘A2D1 15TT’, ‘A2D2’, ‘A3D1 before’, ‘A3D1 15TT’, ‘A3D2’, ‘A4D1 before’, ‘A4D1 15TT’, ‘A4D2’): FVC

(% predicted) for whole study population

Table C 86: Respiratory Testing (‘A1D1 before’, ‘A1D1 15TT’, ‘A1D2’, ‘A2D1 before’, ‘A2D1 15TT’, ‘A2D2’, ‘A3D1 before’, ‘A3D1 15TT’, ‘A3D2’, ‘A4D1 before’, ‘A4D1 15TT’, ‘A4D2’): FVC

(% predicted) for stratum male

Table C 87: Respiratory Testing (‘A1D1 before’, ‘A1D1 15TT’, ‘A1D2’, ‘A2D1 before’, ‘A2D1 15TT’, ‘A2D2’, ‘A3D1 before’, ‘A3D1 15TT’, ‘A3D2’, ‘A4D1 before’, ‘A4D1 15TT’, ‘A4D2’): FVC

(% predicted) for stratum female

Table C 88: Respiratory Testing (‘A1D1 before’, ‘A1D1 15TT’, ‘A1D2’, ‘A2D1 before’, ‘A2D1 15TT’, ‘A2D2’, ‘A3D1 before’, ‘A3D1 15TT’, ‘A3D2’, ‘A4D1 before’, ‘A4D1 15TT’, ‘A4D2’): FVC

(% predicted) for whole study population by treatment A, B, C, D

Table C 89: Respiratory Testing (‘A1D1 before’, ‘A1D1 15TT’, ‘A1D2’, ‘A2D1 before’, ‘A2D1 15TT’, ‘A2D2’, ‘A3D1 before’, ‘A3D1 15TT’, ‘A3D2’, ‘A4D1 before’, ‘A4D1 15TT’, ‘A4D2’): FVC

(% predicted) for stratum male by treatment A, B, C, D

Table C 90: Respiratory Testing (‘A1D1 before’, ‘A1D1 15TT’, ‘A1D2’, ‘A2D1 before’, ‘A2D1 15TT’, ‘A2D2’, ‘A3D1 before’, ‘A3D1 15TT’, ‘A3D2’, ‘A4D1 before’, ‘A4D1 15TT’, ‘A4D2’): FVC

(% predicted) for stratum female by treatment A, B, C, D

Table C 91: Respiratory Testing (‘A1D1 before’, ‘A1D1 15TT’, ‘A1D2’, ‘A2D1 before’, ‘A2D1 15TT’, ‘A2D2’, ‘A3D1 before’, ‘A3D1 15TT’, ‘A3D2’, ‘A4D1 before’, ‘A4D1 15TT’, ‘A4D2’): FEV1 (measured value) for whole study population

Table C 92: Respiratory Testing (‘A1D1 before’, ‘A1D1 15TT’, ‘A1D2’, ‘A2D1 before’, ‘A2D1 15TT’, ‘A2D2’, ‘A3D1 before’, ‘A3D1 15TT’, ‘A3D2’, ‘A4D1 before’, ‘A4D1 15TT’, ‘A4D2’): FEV1 (measured value) for stratum male

Table C 93: Respiratory Testing (‘A1D1 before’, ‘A1D1 15TT’, ‘A1D2’, ‘A2D1 before’, ‘A2D1 15TT’, ‘A2D2’, ‘A3D1 before’, ‘A3D1 15TT’, ‘A3D2’, ‘A4D1 before’, ‘A4D1 15TT’, ‘A4D2’): FEV1 (measured value) for stratum female

Table C 94: Respiratory Testing (‘A1D1 before’, ‘A1D1 15TT’, ‘A1D2’, ‘A2D1 before’, ‘A2D1 15TT’, ‘A2D2’, ‘A3D1 before’, ‘A3D1 15TT’, ‘A3D2’, ‘A4D1 before’, ‘A4D1 15TT’, ‘A4D2’): FEV1 (measured value) for whole study population by treatment A, B, C, D

Table C 95: Respiratory Testing (‘A1D1 before’, ‘A1D1 15TT’, ‘A1D2’, ‘A2D1 before’, ‘A2D1 15TT’, ‘A2D2’, ‘A3D1 before’, ‘A3D1 15TT’, ‘A3D2’, ‘A4D1 before’, ‘A4D1 15TT’, ‘A4D2’): FEV1 (measured value) for stratum male by treatment A, B, C, D

Table C 96: Respiratory Testing (‘A1D1 before’, ‘A1D1 15TT’, ‘A1D2’, ‘A2D1 before’, ‘A2D1 15TT’, ‘A2D2’, ‘A3D1 before’, ‘A3D1 15TT’, ‘A3D2’, ‘A4D1 before’, ‘A4D1 15TT’, ‘A4D2’): FEV1 (measured value) for stratum female by treatment A, B, C, D

Table C 97: Respiratory Testing (‘A1D1 before’, ‘A1D1 15TT’, ‘A1D2’, ‘A2D1 before’, ‘A2D1 15TT’, ‘A2D2’, ‘A3D1 before’, ‘A3D1 15TT’, ‘A3D2’, ‘A4D1 before’, ‘A4D1 15TT’, ‘A4D2’): FEV1

(% predicted) for whole study population

Table C 98: Respiratory Testing (‘A1D1 before’, ‘A1D1 15TT’, ‘A1D2’, ‘A2D1 before’, ‘A2D1 15TT’, ‘A2D2’, ‘A3D1 before’, ‘A3D1 15TT’, ‘A3D2’, ‘A4D1 before’, ‘A4D1 15TT’, ‘A4D2’): FEV1

(% predicted) for stratum male

Table C 99: Respiratory Testing (‘A1D1 before’, ‘A1D1 15TT’, ‘A1D2’, ‘A2D1 before’, ‘A2D1 15TT’, ‘A2D2’, ‘A3D1 before’, ‘A3D1 15TT’, ‘A3D2’, ‘A4D1 before’, ‘A4D1 15TT’, ‘A4D2’): FEV1

(% predicted) for stratum female

Table C 100: Respiratory Testing (‘A1D1 before’, ‘A1D1 15TT’, ‘A1D2’, ‘A2D1 before’, ‘A2D1 15TT’, ‘A2D2’, ‘A3D1 before’, ‘A3D1 15TT’, ‘A3D2’, ‘A4D1 before’, ‘A4D1 15TT’, ‘A4D2’): FEV1

(% predicted) for whole study population by treatment A, B, C, D

Table C 101: Respiratory Testing (‘A1D1 before’, ‘A1D1 15TT’, ‘A1D2’, ‘A2D1 before’, ‘A2D1 15TT’, ‘A2D2’, ‘A3D1 before’, ‘A3D1 15TT’, ‘A3D2’, ‘A4D1 before’, ‘A4D1 15TT’, ‘A4D2’): FEV1

(% predicted) for stratum male by treatment A, B, C, D

Table C 102: Respiratory Testing (‘A1D1 before’, ‘A1D1 15TT’, ‘A1D2’, ‘A2D1 before’, ‘A2D1 15TT’, ‘A2D2’, ‘A3D1 before’, ‘A3D1 15TT’, ‘A3D2’, ‘A4D1 before’, ‘A4D1 15TT’, ‘A4D2’): FEV1

(% predicted) for stratum female by treatment A, B, C, D

Table C 103: Respiratory Testing (‘A1D1 before’, ‘A1D1 15TT’, ‘A1D2’, ‘A2D1 before’, ‘A2D1 15TT’, ‘A2D2’, ‘A3D1 before’, ‘A3D1 15TT’, ‘A3D2’, ‘A4D1 before’, ‘A4D1 15TT’, ‘A4D2’): MEF25 (measured value) for whole study population

Table C 104: Respiratory Testing (‘A1D1 before’, ‘A1D1 15TT’, ‘A1D2’, ‘A2D1 before’, ‘A2D1 15TT’, ‘A2D2’, ‘A3D1 before’, ‘A3D1 15TT’, ‘A3D2’, ‘A4D1 before’, ‘A4D1 15TT’, ‘A4D2’): MEF25 (measured value) for stratum male

Table C 105: Respiratory Testing (‘A1D1 before’, ‘A1D1 15TT’, ‘A1D2’, ‘A2D1 before’, ‘A2D1 15TT’, ‘A2D2’, ‘A3D1 before’, ‘A3D1 15TT’, ‘A3D2’, ‘A4D1 before’, ‘A4D1 15TT’, ‘A4D2’): MEF25 (measured value) for stratum female

Table C 106: Respiratory Testing (‘A1D1 before’, ‘A1D1 15TT’, ‘A1D2’, ‘A2D1 before’, ‘A2D1 15TT’, ‘A2D2’, ‘A3D1 before’, ‘A3D1 15TT’, ‘A3D2’, ‘A4D1 before’, ‘A4D1 15TT’, ‘A4D2’): MEF25 (measured value) for whole study population by treatment A, B, C, D

Table C 107: Respiratory Testing (‘A1D1 before’, ‘A1D1 15TT’, ‘A1D2’, ‘A2D1 before’, ‘A2D1 15TT’, ‘A2D2’, ‘A3D1 before’, ‘A3D1 15TT’, ‘A3D2’, ‘A4D1 before’, ‘A4D1 15TT’, ‘A4D2’): MEF25 (measured value) for stratum male by treatment A, B, C, D

Table C 108: Respiratory Testing (‘A1D1 before’, ‘A1D1 15TT’, ‘A1D2’, ‘A2D1 before’, ‘A2D1 15TT’, ‘A2D2’, ‘A3D1 before’, ‘A3D1 15TT’, ‘A3D2’, ‘A4D1 before’, ‘A4D1 15TT’, ‘A4D2’): MEF25 (measured value) for stratum female by treatment A, B, C, D

Table C 109: Respiratory Testing (‘A1D1 before’, ‘A1D1 15TT’, ‘A1D2’, ‘A2D1 before’, ‘A2D1 15TT’, ‘A2D2’, ‘A3D1 before’, ‘A3D1 15TT’, ‘A3D2’, ‘A4D1 before’, ‘A4D1 15TT’, ‘A4D2’): MEF25 (% predicted) for whole study population

Table C 110: Respiratory Testing (‘A1D1 before’, ‘A1D1 15TT’, ‘A1D2’, ‘A2D1 before’, ‘A2D1 15TT’, ‘A2D2’, ‘A3D1 before’, ‘A3D1 15TT’, ‘A3D2’, ‘A4D1 before’, ‘A4D1 15TT’, ‘A4D2’): MEF25 (% predicted) for stratum male

Table C 111: Respiratory Testing (‘A1D1 before’, ‘A1D1 15TT’, ‘A1D2’, ‘A2D1 before’, ‘A2D1 15TT’, ‘A2D2’, ‘A3D1 before’, ‘A3D1 15TT’, ‘A3D2’, ‘A4D1 before’, ‘A4D1 15TT’, ‘A4D2’): MEF25 (% predicted) for stratum female

Table C 112: Respiratory Testing (‘A1D1 before’, ‘A1D1 15TT’, ‘A1D2’, ‘A2D1 before’, ‘A2D1 15TT’, ‘A2D2’, ‘A3D1 before’, ‘A3D1 15TT’, ‘A3D2’, ‘A4D1 before’, ‘A4D1 15TT’, ‘A4D2’): MEF25 (% predicted) for whole study population by treatment A, B, C, D

Table C 113: Respiratory Testing (‘A1D1 before’, ‘A1D1 15TT’, ‘A1D2’, ‘A2D1 before’, ‘A2D1 15TT’, ‘A2D2’, ‘A3D1 before’, ‘A3D1 15TT’, ‘A3D2’, ‘A4D1 before’, ‘A4D1 15TT’, ‘A4D2’): MEF25 (% predicted) for stratum male by treatment A, B, C, D

Table C 114: Respiratory Testing (‘A1D1 before’, ‘A1D1 15TT’, ‘A1D2’, ‘A2D1 before’, ‘A2D1 15TT’, ‘A2D2’, ‘A3D1 before’, ‘A3D1 15TT’, ‘A3D2’, ‘A4D1 before’, ‘A4D1 15TT’, ‘A4D2’): MEF25 (% predicted) for stratum female by treatment A, B, C, D

Table C 115: Respiratory Testing (‘A1D1 before’, ‘A1D1 15TT’, ‘A1D2’, ‘A2D1 before’, ‘A2D1 15TT’, ‘A2D2’, ‘A3D1 before’, ‘A3D1 15TT’, ‘A3D2’, ‘A4D1 before’, ‘A4D1 15TT’, ‘A4D2’): TLC (measured value) for whole study population

Table C 116: Respiratory Testing (‘A1D1 before’, ‘A1D1 15TT’, ‘A1D2’, ‘A2D1 before’, ‘A2D1 15TT’, ‘A2D2’, ‘A3D1 before’, ‘A3D1 15TT’, ‘A3D2’, ‘A4D1 before’, ‘A4D1 15TT’, ‘A4D2’): TLC (measured value) for stratum male

Table C 117: Respiratory Testing (‘A1D1 before’, ‘A1D1 15TT’, ‘A1D2’, ‘A2D1 before’, ‘A2D1 15TT’, ‘A2D2’, ‘A3D1 before’, ‘A3D1 15TT’, ‘A3D2’, ‘A4D1 before’, ‘A4D1 15TT’, ‘A4D2’): TLC (measured value) for stratum female

Table C 118: Respiratory Testing (‘A1D1 before’, ‘A1D1 15TT’, ‘A1D2’, ‘A2D1 before’, ‘A2D1 15TT’, ‘A2D2’, ‘A3D1 before’, ‘A3D1 15TT’, ‘A3D2’, ‘A4D1 before’, ‘A4D1 15TT’, ‘A4D2’): TLC (measured value) for whole study population by treatment A, B, C, D

Table C 119: Respiratory Testing (‘A1D1 before’, ‘A1D1 15TT’, ‘A1D2’, ‘A2D1 before’, ‘A2D1 15TT’, ‘A2D2’, ‘A3D1 before’, ‘A3D1 15TT’, ‘A3D2’, ‘A4D1 before’, ‘A4D1 15TT’, ‘A4D2’): TLC (measured value) for stratum male by treatment A, B, C, D

Table C 120: Respiratory Testing (‘A1D1 before’, ‘A1D1 15TT’, ‘A1D2’, ‘A2D1 before’, ‘A2D1 15TT’, ‘A2D2’, ‘A3D1 before’, ‘A3D1 15TT’, ‘A3D2’, ‘A4D1 before’, ‘A4D1 15TT’, ‘A4D2’): TLC (measured value) for stratum female by treatment A, B, C, D

Table C 121: Respiratory Testing (‘A1D1 before’, ‘A1D1 15TT’, ‘A1D2’, ‘A2D1 before’, ‘A2D1 15TT’, ‘A2D2’, ‘A3D1 before’, ‘A3D1 15TT’, ‘A3D2’, ‘A4D1 before’, ‘A4D1 15TT’, ‘A4D2’): TLC

(% predicted) for whole study population

Table C 122: Respiratory Testing (‘A1D1 before’, ‘A1D1 15TT’, ‘A1D2’, ‘A2D1 before’, ‘A2D1 15TT’, ‘A2D2’, ‘A3D1 before’, ‘A3D1 15TT’, ‘A3D2’, ‘A4D1 before’, ‘A4D1 15TT’, ‘A4D2’): TLC

(% predicted) for stratum male

Table C 123: Respiratory Testing (‘A1D1 before’, ‘A1D1 15TT’, ‘A1D2’, ‘A2D1 before’, ‘A2D1 15TT’, ‘A2D2’, ‘A3D1 before’, ‘A3D1 15TT’, ‘A3D2’, ‘A4D1 before’, ‘A4D1 15TT’, ‘A4D2’): TLC

(% predicted) for stratum female

Table C 124: Respiratory Testing (‘A1D1 before’, ‘A1D1 15TT’, ‘A1D2’, ‘A2D1 before’, ‘A2D1 15TT’, ‘A2D2’, ‘A3D1 before’, ‘A3D1 15TT’, ‘A3D2’, ‘A4D1 before’, ‘A4D1 15TT’, ‘A4D2’): TLC

(% predicted) for whole study population by treatment A, B, C, D

Table C 125: Respiratory Testing (‘A1D1 before’, ‘A1D1 15TT’, ‘A1D2’, ‘A2D1 before’, ‘A2D1 15TT’, ‘A2D2’, ‘A3D1 before’, ‘A3D1 15TT’, ‘A3D2’, ‘A4D1 before’, ‘A4D1 15TT’, ‘A4D2’): TLC

(% predicted) for stratum male by treatment A, B, C, D

Table C 126: Respiratory Testing (‘A1D1 before’, ‘A1D1 15TT’, ‘A1D2’, ‘A2D1 before’, ‘A2D1 15TT’, ‘A2D2’, ‘A3D1 before’, ‘A3D1 15TT’, ‘A3D2’, ‘A4D1 before’, ‘A4D1 15TT’, ‘A4D2’): TLC

(% predicted) for stratum female by treatment A, B, C, D

Table C 127: Respiratory Testing (‘A1D1 before’, ‘A1D1 15TT’, ‘A1D2’, ‘A2D1 before’, ‘A2D1 15TT’, ‘A2D2’, ‘A3D1 before’, ‘A3D1 15TT’, ‘A3D2’, ‘A4D1 before’, ‘A4D1 15TT’, ‘A4D2’): FRC (measured value) for whole study population

Table C 128: Respiratory Testing (‘A1D1 before’, ‘A1D1 15TT’, ‘A1D2’, ‘A2D1 before’, ‘A2D1 15TT’, ‘A2D2’, ‘A3D1 before’, ‘A3D1 15TT’, ‘A3D2’, ‘A4D1 before’, ‘A4D1 15TT’, ‘A4D2’): FRC (measured value) for stratum male

Table C 129: Respiratory Testing (‘A1D1 before’, ‘A1D1 15TT’, ‘A1D2’, ‘A2D1 before’, ‘A2D1 15TT’, ‘A2D2’, ‘A3D1 before’, ‘A3D1 15TT’, ‘A3D2’, ‘A4D1 before’, ‘A4D1 15TT’, ‘A4D2’): FRC (measured value) for stratum female

Table C 130: Respiratory Testing (‘A1D1 before’, ‘A1D1 15TT’, ‘A1D2’, ‘A2D1 before’, ‘A2D1 15TT’, ‘A2D2’, ‘A3D1 before’, ‘A3D1 15TT’, ‘A3D2’, ‘A4D1 before’, ‘A4D1 15TT’, ‘A4D2’): FRC (measured value) for whole study population by treatment A, B, C, D

Table C 131: Respiratory Testing (‘A1D1 before’, ‘A1D1 15TT’, ‘A1D2’, ‘A2D1 before’, ‘A2D1 15TT’, ‘A2D2’, ‘A3D1 before’, ‘A3D1 15TT’, ‘A3D2’, ‘A4D1 before’, ‘A4D1 15TT’, ‘A4D2’): FRC (measured value) for stratum male by treatment A, B, C, D

Table C 132: Respiratory Testing (‘A1D1 before’, ‘A1D1 15TT’, ‘A1D2’, ‘A2D1 before’, ‘A2D1 15TT’, ‘A2D2’, ‘A3D1 before’, ‘A3D1 15TT’, ‘A3D2’, ‘A4D1 before’, ‘A4D1 15TT’, ‘A4D2’): FRC (measured value) for stratum female by treatment A, B, C, D

Table C 133: Respiratory Testing (‘A1D1 before’, ‘A1D1 15TT’, ‘A1D2’, ‘A2D1 before’, ‘A2D1 15TT’, ‘A2D2’, ‘A3D1 before’, ‘A3D1 15TT’, ‘A3D2’, ‘A4D1 before’, ‘A4D1 15TT’, ‘A4D2’): FRC

(% predicted) for whole study population

Table C 134: Respiratory Testing (‘A1D1 before’, ‘A1D1 15TT’, ‘A1D2’, ‘A2D1 before’, ‘A2D1 15TT’, ‘A2D2’, ‘A3D1 before’, ‘A3D1 15TT’, ‘A3D2’, ‘A4D1 before’, ‘A4D1 15TT’, ‘A4D2’): FRC

(% predicted) for stratum male

Table C 135: Respiratory Testing (‘A1D1 before’, ‘A1D1 15TT’, ‘A1D2’, ‘A2D1 before’, ‘A2D1 15TT’, ‘A2D2’, ‘A3D1 before’, ‘A3D1 15TT’, ‘A3D2’, ‘A4D1 before’, ‘A4D1 15TT’, ‘A4D2’): FRC

(% predicted) for stratum female

Table C 136: Respiratory Testing (‘A1D1 before’, ‘A1D1 15TT’, ‘A1D2’, ‘A2D1 before’, ‘A2D1 15TT’, ‘A2D2’, ‘A3D1 before’, ‘A3D1 15TT’, ‘A3D2’, ‘A4D1 before’, ‘A4D1 15TT’, ‘A4D2’): FRC

(% predicted) for whole study population by treatment A, B, C, D

Table C 137: Respiratory Testing (‘A1D1 before’, ‘A1D1 15TT’, ‘A1D2’, ‘A2D1 before’, ‘A2D1 15TT’, ‘A2D2’, ‘A3D1 before’, ‘A3D1 15TT’, ‘A3D2’, ‘A4D1 before’, ‘A4D1 15TT’, ‘A4D2’): FRC

(% predicted) for stratum male by treatment A, B, C, D

Table C 138: Respiratory Testing (‘A1D1 before’, ‘A1D1 15TT’, ‘A1D2’, ‘A2D1 before’, ‘A2D1 15TT’, ‘A2D2’, ‘A3D1 before’, ‘A3D1 15TT’, ‘A3D2’, ‘A4D1 before’, ‘A4D1 15TT’, ‘A4D2’): FRC

(% predicted) for stratum female by treatment A, B, C, D

Table C 139: Respiratory Testing (‘A1D1 before’, ‘A1D1 15TT’, ‘A1D2’, ‘A2D1 before’, ‘A2D1 15TT’, ‘A2D2’, ‘A3D1 before’, ‘A3D1 15TT’, ‘A3D2’, ‘A4D1 before’, ‘A4D1 15TT’, ‘A4D2’): RV (measured value) for whole study population

Table C 140: Respiratory Testing (‘A1D1 before’, ‘A1D1 15TT’, ‘A1D2’, ‘A2D1 before’, ‘A2D1 15TT’, ‘A2D2’, ‘A3D1 before’, ‘A3D1 15TT’, ‘A3D2’, ‘A4D1 before’, ‘A4D1 15TT’, ‘A4D2’): RV (measured value) for stratum male

Table C 141: Respiratory Testing (‘A1D1 before’, ‘A1D1 15TT’, ‘A1D2’, ‘A2D1 before’, ‘A2D1 15TT’, ‘A2D2’, ‘A3D1 before’, ‘A3D1 15TT’, ‘A3D2’, ‘A4D1 before’, ‘A4D1 15TT’, ‘A4D2’): RV (measured value) for stratum female

Table C 142: Respiratory Testing (‘A1D1 before’, ‘A1D1 15TT’, ‘A1D2’, ‘A2D1 before’, ‘A2D1 15TT’, ‘A2D2’, ‘A3D1 before’, ‘A3D1 15TT’, ‘A3D2’, ‘A4D1 before’, ‘A4D1 15TT’, ‘A4D2’): RV (measured value) for whole study population by treatment A, B, C, D

Table C 143: Respiratory Testing (‘A1D1 before’, ‘A1D1 15TT’, ‘A1D2’, ‘A2D1 before’, ‘A2D1 15TT’, ‘A2D2’, ‘A3D1 before’, ‘A3D1 15TT’, ‘A3D2’, ‘A4D1 before’, ‘A4D1 15TT’, ‘A4D2’): RV (measured value) for stratum male by treatment A, B, C, D

Table C 144: Respiratory Testing (‘A1D1 before’, ‘A1D1 15TT’, ‘A1D2’, ‘A2D1 before’, ‘A2D1 15TT’, ‘A2D2’, ‘A3D1 before’, ‘A3D1 15TT’, ‘A3D2’, ‘A4D1 before’, ‘A4D1 15TT’, ‘A4D2’): RV (measured value) for stratum female by treatment A, B, C, D

Table C 145: Respiratory Testing (‘A1D1 before’, ‘A1D1 15TT’, ‘A1D2’, ‘A2D1 before’, ‘A2D1 15TT’, ‘A2D2’, ‘A3D1 before’, ‘A3D1 15TT’, ‘A3D2’, ‘A4D1 before’, ‘A4D1 15TT’, ‘A4D2’): RV
(% predicted) for whole study population

Table C 146: Respiratory Testing (‘A1D1 before’, ‘A1D1 15TT’, ‘A1D2’, ‘A2D1 before’, ‘A2D1 15TT’, ‘A2D2’, ‘A3D1 before’, ‘A3D1 15TT’, ‘A3D2’, ‘A4D1 before’, ‘A4D1 15TT’, ‘A4D2’): RV
(% predicted) for stratum male

Table C 147: Respiratory Testing (‘A1D1 before’, ‘A1D1 15TT’, ‘A1D2’, ‘A2D1 before’, ‘A2D1 15TT’, ‘A2D2’, ‘A3D1 before’, ‘A3D1 15TT’, ‘A3D2’, ‘A4D1 before’, ‘A4D1 15TT’, ‘A4D2’): RV

(% predicted) for stratum female

Table C 148: Respiratory Testing (‘A1D1 before’, ‘A1D1 15TT’, ‘A1D2’, ‘A2D1 before’, ‘A2D1 15TT’, ‘A2D2’, ‘A3D1 before’, ‘A3D1 15TT’, ‘A3D2’, ‘A4D1 before’, ‘A4D1 15TT’, ‘A4D2’): RV

(% predicted) for whole study population by treatment A, B, C, D

Table C 149: Respiratory Testing (‘A1D1 before’, ‘A1D1 15TT’, ‘A1D2’, ‘A2D1 before’, ‘A2D1 15TT’, ‘A2D2’, ‘A3D1 before’, ‘A3D1 15TT’, ‘A3D2’, ‘A4D1 before’, ‘A4D1 15TT’, ‘A4D2’): RV

(% predicted) for stratum male by treatment A, B, C, D

Table C 150: Respiratory Testing (‘A1D1 before’, ‘A1D1 15TT’, ‘A1D2’, ‘A2D1 before’, ‘A2D1 15TT’, ‘A2D2’, ‘A3D1 before’, ‘A3D1 15TT’, ‘A3D2’, ‘A4D1 before’, ‘A4D1 15TT’, ‘A4D2’): RV

(% predicted) for stratum female by treatment A, B, C, D

Table C 151: Respiratory Testing (‘A1D1 before’, ‘A1D1 15TT’, ‘A1D2’, ‘A2D1 before’, ‘A2D1 15TT’, ‘A2D2’, ‘A3D1 before’, ‘A3D1 15TT’, ‘A3D2’, ‘A4D1 before’, ‘A4D1 15TT’, ‘A4D2’): sRaw (measured value) for whole study population

Table C 152: Respiratory Testing (‘A1D1 before’, ‘A1D1 15TT’, ‘A1D2’, ‘A2D1 before’, ‘A2D1 15TT’, ‘A2D2’, ‘A3D1 before’, ‘A3D1 15TT’, ‘A3D2’, ‘A4D1 before’, ‘A4D1 15TT’, ‘A4D2’): sRaw (measured value) for stratum male

Table C 153: Respiratory Testing (‘A1D1 before’, ‘A1D1 15TT’, ‘A1D2’, ‘A2D1 before’, ‘A2D1 15TT’, ‘A2D2’, ‘A3D1 before’, ‘A3D1 15TT’, ‘A3D2’, ‘A4D1 before’, ‘A4D1 15TT’, ‘A4D2’): sRaw (measured value) for stratum female

Table C 154: Respiratory Testing (‘A1D1 before’, ‘A1D1 15TT’, ‘A1D2’, ‘A2D1 before’, ‘A2D1 15TT’, ‘A2D2’, ‘A3D1 before’, ‘A3D1 15TT’, ‘A3D2’, ‘A4D1 before’, ‘A4D1 15TT’, ‘A4D2’): sRaw (measured value) for whole study population by treatment A, B, C, D

Table C 155: Respiratory Testing (‘A1D1 before’, ‘A1D1 15TT’, ‘A1D2’, ‘A2D1 before’, ‘A2D1 15TT’, ‘A2D2’, ‘A3D1 before’, ‘A3D1 15TT’, ‘A3D2’, ‘A4D1 before’, ‘A4D1 15TT’, ‘A4D2’): sRaw (measured value) for stratum male by treatment A, B, C, D

Table C 156: Respiratory Testing (‘A1D1 before’, ‘A1D1 15TT’, ‘A1D2’, ‘A2D1 before’, ‘A2D1 15TT’, ‘A2D2’, ‘A3D1 before’, ‘A3D1 15TT’, ‘A3D2’, ‘A4D1 before’, ‘A4D1 15TT’, ‘A4D2’): sRaw (measured value) for stratum female by treatment A, B, C, D

Analogue tables will be created for the following differences in the parameters FVC (measured value), FVC (% predicted), FEV1 (measured value), FEV1 (% predicted), MEF25 (measured value), MEF25 (% predicted) , TLC (measured value), TLC (% predicted), FRC (measured value), FRC (% predicted), RV (measured value), RV (% predicted), sRaw (measured value): ‘AxD1 15TT’ minus ‘AxD1 before’, ‘AxD2’ minus ‘AxD1 before’, ‘AxD2’ minus ‘AxD1 15TT’, x labels the study arm, x=1, 2, 3, 4.

**Cardiopulmonary function (ECG):**

Table C 157: ECG (‘A1D1 15TT’, ‘A1D2’, ‘A2D1 15TT’, ‘A2D2’, ‘A3D1 15TT’, ‘A3D2’, ‘A4D1 15TT’, ‘A4D2’): frequency of ‘Rhythm disorders present’ and specification in case of yes (only listing) for whole study population

Table C 158: ECG (‘A1D1 15TT’, ‘A1D2’, ‘A2D1 15TT’, ‘A2D2’, ‘A3D1 15TT’, ‘A3D2’, ‘A4D1 15TT’, ‘A4D2’): frequency of ‘Rhythm disorders present’ and specification in case of yes (only listing) for stratum male

Table C 159: ECG (‘A1D1 15TT’, ‘A1D2’, ‘A2D1 15TT’, ‘A2D2’, ‘A3D1 15TT’, ‘A3D2’, ‘A4D1 15TT’, ‘A4D2’): frequency of ‘Rhythm disorders present’ and specification in case of yes (only listing) for stratum female

Table C 160: ECG (‘A1D1 15TT’, ‘A1D2’, ‘A2D1 15TT’, ‘A2D2’, ‘A3D1 15TT’, ‘A3D2’, ‘A4D1 15TT’, ‘A4D2’): frequency of ‘Rhythm disorders present’ and specification in case of yes (only listing) for whole study population by treatment A, B, C, D

Table C 161: ECG (‘A1D1 15TT’, ‘A1D2’, ‘A2D1 15TT’, ‘A2D2’, ‘A3D1 15TT’, ‘A3D2’, ‘A4D1 15TT’, ‘A4D2’): frequency of ‘Rhythm disorders present’ and specification in case of yes (only listing) for stratum male by treatment A, B, C, D

Table C 162: ECG (‘A1D1 15TT’, ‘A1D2’, ‘A2D1 15TT’, ‘A2D2’, ‘A3D1 15TT’, ‘A3D2’, ‘A4D1 15TT’, ‘A4D2’): frequency of ‘Rhythm disorders present’ and specification in case of yes (only listing) for stratum female by treatment A, B, C, D

Table C 163: ECG (‘A1D1 15TT’, ‘A1D2’, ‘A2D1 15TT’, ‘A2D2’, ‘A3D1 15TT’, ‘A3D2’, ‘A4D1 15TT’, ‘A4D2’): frequency for ‘Study exclusion necessary?’ and ‘Subject qualified to continue?’ for whole study population

Table C 164: ECG (‘A1D1 15TT’, ‘A1D2’, ‘A2D1 15TT’, ‘A2D2’, ‘A3D1 15TT’, ‘A3D2’, ‘A4D1 15TT’, ‘A4D2’): frequency for ‘Study exclusion necessary?’ and ‘Subject qualified to continue?’ for stratum male

Table C 165: ECG (‘A1D1 15TT’, ‘A1D2’, ‘A2D1 15TT’, ‘A2D2’, ‘A3D1 15TT’, ‘A3D2’, ‘A4D1 15TT’, ‘A4D2’): frequency for ‘Study exclusion necessary?’ and ‘Subject qualified to continue?’ for stratum female

Table C 166: ECG (‘A1D1 15TT’, ‘A1D2’, ‘A2D1 15TT’, ‘A2D2’, ‘A3D1 15TT’, ‘A3D2’, ‘A4D1 15TT’, ‘A4D2’): frequency for ‘Study exclusion necessary?’ and ‘Subject qualified to continue?’ for whole study population by treatment A, B, C, D

Table C 167: ECG (‘A1D1 15TT’, ‘A1D2’, ‘A2D1 15TT’, ‘A2D2’, ‘A3D1 15TT’, ‘A3D2’, ‘A4D1 15TT’, ‘A4D2’): frequency for ‘Study exclusion necessary?’ and ‘Subject qualified to continue?’ for stratum male by treatment A, B, C, D

Table C 168: ECG (‘A1D1 15TT’, ‘A1D2’, ‘A2D1 15TT’, ‘A2D2’, ‘A3D1 15TT’, ‘A3D2’, ‘A4D1 15TT’, ‘A4D2’): frequency for ‘Study exclusion necessary?’ and ‘Subject qualified to continue?’ for stratum female by treatment A, B, C, D

Table C 169: ECG (‘A1D1 15TT’, ‘A1D2’, ‘A2D1 15TT’, ‘A2D2’, ‘A3D1 15TT’, ‘A3D2’, ‘A4D1 15TT’, ‘A4D2’): QTc for whole study population

Table C 170: ECG (‘A1D1 15TT’, ‘A1D2’, ‘A2D1 15TT’, ‘A2D2’, ‘A3D1 15TT’, ‘A3D2’, ‘A4D1 15TT’, ‘A4D2’): QTc for stratum male

Table C 171: ECG (‘A1D1 15TT’, ‘A1D2’, ‘A2D1 15TT’, ‘A2D2’, ‘A3D1 15TT’, ‘A3D2’, ‘A4D1 15TT’, ‘A4D2’): QTc for stratum female

Table C 172: ECG (‘A1D1 15TT’, ‘A1D2’, ‘A2D1 15TT’, ‘A2D2’, ‘A3D1 15TT’, ‘A3D2’, ‘A4D1 15TT’, ‘A4D2’): QTc for whole study population by treatment A, B, C, D

Table C 173: ECG (‘A1D1 15TT’, ‘A1D2’, ‘A2D1 15TT’, ‘A2D2’, ‘A3D1 15TT’, ‘A3D2’, ‘A4D1 15TT’, ‘A4D2’): QTc for stratum male by treatment A, B, C, D

Table C 174: ECG (‘A1D1 15TT’, ‘A1D2’, ‘A2D1 15TT’, ‘A2D2’, ‘A3D1 15TT’, ‘A3D2’, ‘A4D1 15TT’, ‘A4D2’): QTc for stratum female by treatment A, B, C, D

Analogue tables will be created for the difference ‘AxD2’ minus ‘AxD1 15TT’ in the parameter QTc, x labels the study arm, x=1, 2, 3, 4.

**Average power and peak power during time trial:**

Table C 175: Average Power and peak power (‘A1D1’, ‘A2D1’, ‘A3D1’, ‘A4D1’): average power output for whole study population

Table C 176: Average Power and peak power (‘A1D1’, ‘A2D1’, ‘A3D1’, ‘A4D1’): average power output for stratum male

Table C 177: Average Power and peak power (‘A1D1’, ‘A2D1’, ‘A3D1’, ‘A4D1’): average power output for stratum female

Table C 178: Average Power and peak power (‘A1D1’, ‘A2D1’, ‘A3D1’, ‘A4D1’): average power output for whole study population by treatment A, B, C, D

Table C 179: Average Power and peak power (‘A1D1’, ‘A2D1’, ‘A3D1’, ‘A4D1’): average power output for whole study population for stratum male by treatment A, B, C, D

Table C 180: Average Power and peak power (‘A1D1’, ‘A2D1’, ‘A3D1’, ‘A4D1’): average power output for whole study population for stratum female by treatment A, B, C, D

Table C 181: Average Power and peak power (‘A1D1’, ‘A2D1’, ‘A3D1’, ‘A4D1’): relative average power output for whole study population

Table C 182: Average Power and peak power (‘A1D1’, ‘A2D1’, ‘A3D1’, ‘A4D1’): relative average power output for stratum male

Table C 183: Average Power and peak power (‘A1D1’, ‘A2D1’, ‘A3D1’, ‘A4D1’): average power output for stratum female

Table C 184: Average Power and peak power (‘A1D1’, ‘A2D1’, ‘A3D1’, ‘A4D1’): relative average power output for whole study population by treatment A, B, C, D

Table C 185: Average Power and peak power (‘A1D1’, ‘A2D1’, ‘A3D1’, ‘A4D1’): relative average power output for stratum male by treatment A, B, C, D

Table C 186: Average Power and peak power (‘A1D1’, ‘A2D1’, ‘A3D1’, ‘A4D1’): relative average power output for stratum female by treatment A, B, C, D

Table C 187: Average Power and peak power (‘A1D1’, ‘A2D1’, ‘A3D1’, ‘A4D1’): peak power for whole study population

Table C 188: Average Power and peak power (‘A1D1’, ‘A2D1’, ‘A3D1’, ‘A4D1’): peak power for stratum male

Table C 189: Average Power and peak power (‘A1D1’, ‘A2D1’, ‘A3D1’, ‘A4D1’): peak power for stratum female

Table C 190: Average Power and peak power (‘A1D1’, ‘A2D1’, ‘A3D1’, ‘A4D1’): peak power for whole study population by treatment A, B, C, D

Table C 191: Average Power and peak power (‘A1D1’, ‘A2D1’, ‘A3D1’, ‘A4D1’): peak power for stratum male by treatment A, B, C, D

Table C 192: Average Power and peak power (‘A1D1’, ‘A2D1’, ‘A3D1’, ‘A4D1’): peak power for stratum female by treatment A, B, C, D

**Safety**

**Vital Signs:**

Table D 1: Vital signs (‘A1D1 before’, ‘A1D1 10after’, ‘A1D1 15TT’, ‘A1D1 1hTT', ‘A1D1 2hTT’,
‘A1D1 3hTT’, ‘A1D2’): blood pressure systolic

Table D 2: Vital signs (‘A1D1 before’, ‘A1D1 10after’, ‘A1D1 15TT’, ‘A1D1 1hTT', ‘A1D1 2hTT’,
‘A1D1 3hTT’, ‘A1D2’): blood pressure systolic for treatment A

Table D 3: Vital signs (‘A1D1 before’, ‘A1D1 10after’, ‘A1D1 15TT’, ‘A1D1 1hTT', ‘A1D1 2hTT’,
‘A1D1 3hTT’, ‘A1D2’): blood pressure systolic for treatment B

Table D 4: Vital signs (‘A1D1 before’, ‘A1D1 10after’, ‘A1D1 15TT’, ‘A1D1 1hTT', ‘A1D1 2hTT’,
‘A1D1 3hTT’, ‘A1D2’): blood pressure systolic for treatment C

Table D 5: Vital signs (‘A1D1 before’, ‘A1D1 10after’, ‘A1D1 15TT’, ‘A1D1 1hTT', ‘A1D1 2hTT’,
‘A1D1 3hTT’, ‘A1D2’): blood pressure systolic for treatment D

Table D 6: Vital signs (‘A2D1 before’, ‘A2D1 10after’, ‘A2D1 15TT’, ‘A2D1 1hTT', ‘A2D1 2hTT’,
‘A2D1 3hTT’, ‘A2D2’): blood pressure systolic

Table D 7: Vital signs (‘A2D1 before’, ‘A2D1 10after’, ‘A2D1 15TT’, ‘A2D1 1hTT', ‘A2D1 2hTT’,
‘A2D1 3hTT’, ‘A2D2’): blood pressure systolic for treatment A

Table D 8: Vital signs (‘A2D1 before’, ‘A2D1 10after’, ‘A2D1 15TT’, ‘A2D1 1hTT', ‘A2D1 2hTT’,
‘A2D1 3hTT’, ‘A2D2’): blood pressure systolic for treatment B

Table D 9: Vital signs (‘A2D1 before’, ‘A2D1 10after’, ‘A2D1 15TT’, ‘A2D1 1hTT', ‘A2D1 2hTT’,
‘A2D1 3hTT’, ‘A2D2’): blood pressure systolic for treatment C

Table D 10: Vital signs (‘A2D1 before’, ‘A2D1 10after’, ‘A2D1 15TT’, ‘A2D1 1hTT', ‘A2D1 2hTT’,
‘A2D1 3hTT’, ‘A2D2’): blood pressure systolic for treatment D

Table D 11: Vital signs (‘A3D1 before’, ‘A3D1 10after’, ‘A3D1 15TT’, ‘A3D1 1hTT', ‘A3D1 2hTT’,
‘A3D1 3hTT’, ‘A3D2’): blood pressure systolic

Table D 12: Vital signs (‘A3D1 before’, ‘A3D1 10after’, ‘A3D1 15TT’, ‘A3D1 1hTT', ‘A3D1 2hTT’,
‘A3D1 3hTT’, ‘A3D2’): blood pressure systolic for treatment A

Table D 13: Vital signs (‘A3D1 before’, ‘A3D1 10after’, ‘A3D1 15TT’, ‘A3D1 1hTT', ‘A3D1 2hTT’,
‘A3D1 3hTT’, ‘A3D2’): blood pressure systolic for treatment B

Table D 14: Vital signs (‘A3D1 before’, ‘A3D1 10after’, ‘A3D1 15TT’, ‘A3D1 1hTT', ‘A3D1 2hTT’,
‘A3D1 3hTT’, ‘A3D2’): blood pressure systolic for treatment C

Table D 15: Vital signs (‘A3D1 before’, ‘A3D1 10after’, ‘A3D1 15TT’, ‘A3D1 1hTT', ‘A3D1 2hTT’,
‘A3D1 3hTT’, ‘A3D2’): blood pressure systolic for treatment D

Table D 16: Vital signs (‘A4D1 before’, ‘A4D1 10after’, ‘A4D1 15TT’, ‘A4D1 1hTT', ‘A4D1 2hTT’,
‘A4D1 3hTT’, ‘A4D2’): blood pressure systolic

Table D 17: Vital signs (‘A4D1 before’, ‘A4D1 10after’, ‘A4D1 15TT’, ‘A4D1 1hTT', ‘A4D1 2hTT’,
‘A4D1 3hTT’, ‘A4D2’): blood pressure systolic for treatment A

Table D 18: Vital signs (‘A4D1 before’, ‘A4D1 10after’, ‘A4D1 15TT’, ‘A4D1 1hTT', ‘A4D1 2hTT’,
‘A4D1 3hTT’, ‘A4D2’): blood pressure systolic for treatment B

Table D 19: Vital signs (‘A4D1 before’, ‘A4D1 10after’, ‘A4D1 15TT’, ‘A4D1 1hTT', ‘A4D1 2hTT’,
‘A4D1 3hTT’, ‘A4D2’): blood pressure systolic for treatment C

Table D 20: Vital signs (‘A4D1 before’, ‘A4D1 10after’, ‘A4D1 15TT’, ‘A4D1 1hTT', ‘A4D1 2hTT’,
‘A4D1 3hTT’, ‘A4D2’): blood pressure systolic for treatment D

Table D 21: Vital signs (‘A1D1 before’, ‘A1D1 10after’, ‘A1D1 15TT’, ‘A1D1 1hTT', ‘A1D1 2hTT’,
‘A1D1 3hTT’, ‘A1D2’): blood pressure diastolic

Table D 22: Vital signs (‘A1D1 before’, ‘A1D1 10after’, ‘A1D1 15TT’, ‘A1D1 1hTT', ‘A1D1 2hTT’,
‘A1D1 3hTT’, ‘A1D2’): blood pressure diastolic for treatment A

Table D 23: Vital signs (‘A1D1 before’, ‘A1D1 10after’, ‘A1D1 15TT’, ‘A1D1 1hTT', ‘A1D1 2hTT’,
‘A1D1 3hTT’, ‘A1D2’): blood pressure diastolic for treatment B

Table D 24: Vital signs (‘A1D1 before’, ‘A1D1 10after’, ‘A1D1 15TT’, ‘A1D1 1hTT', ‘A1D1 2hTT’,
‘A1D1 3hTT’, ‘A1D2’): blood pressure diastolic for treatment C

Table D 25: Vital signs (‘A1D1 before’, ‘A1D1 10after’, ‘A1D1 15TT’, ‘A1D1 1hTT', ‘A1D1 2hTT’,
‘A1D1 3hTT’, ‘A1D2’): blood pressure diastolic for treatment D

Table D 26: Vital signs (‘A2D1 before’, ‘A2D1 10after’, ‘A2D1 15TT’, ‘A2D1 1hTT', ‘A2D1 2hTT’,
‘A2D1 3hTT’, ‘A2D2’): blood pressure diastolic

Table D 27: Vital signs (‘A2D1 before’, ‘A2D1 10after’, ‘A2D1 15TT’, ‘A2D1 1hTT', ‘A2D1 2hTT’,
‘A2D1 3hTT’, ‘A2D2’): blood pressure diastolic for treatment A

Table D 28: Vital signs (‘A2D1 before’, ‘A2D1 10after’, ‘A2D1 15TT’, ‘A2D1 1hTT', ‘A2D1 2hTT’,
‘A2D1 3hTT’, ‘A2D2’): blood pressure diastolic for treatment B

Table D 29: Vital signs (‘A2D1 before’, ‘A2D1 10after’, ‘A2D1 15TT’, ‘A2D1 1hTT', ‘A2D1 2hTT’,
‘A2D1 3hTT’, ‘A2D2’): blood pressure diastolic for treatment C

Table D 30: Vital signs (‘A2D1 before’, ‘A2D1 10after’, ‘A2D1 15TT’, ‘A2D1 1hTT', ‘A2D1 2hTT’,
‘A2D1 3hTT’, ‘A2D2’): blood pressure diastolic for treatment D

Table D 31: Vital signs (‘A3D1 before’, ‘A3D1 10after’, ‘A3D1 15TT’, ‘A3D1 1hTT', ‘A3D1 2hTT’,
‘A3D1 3hTT’, ‘A3D2’): blood pressure diastolic

Table D 32: Vital signs (‘A3D1 before’, ‘A3D1 10after’, ‘A3D1 15TT’, ‘A3D1 1hTT', ‘A3D1 2hTT’,
‘A3D1 3hTT’, ‘A3D2’): blood pressure diastolic for treatment A

Table D 33: Vital signs (‘A3D1 before’, ‘A3D1 10after’, ‘A3D1 15TT’, ‘A3D1 1hTT', ‘A3D1 2hTT’,
‘A3D1 3hTT’, ‘A3D2’): blood pressure diastolic for treatment B

Table D 34: Vital signs (‘A3D1 before’, ‘A3D1 10after’, ‘A3D1 15TT’, ‘A3D1 1hTT', ‘A3D1 2hTT’,
‘A3D1 3hTT’, ‘A3D2’): blood pressure diastolic for treatment C

Table D 35: Vital signs (‘A3D1 before’, ‘A3D1 10after’, ‘A3D1 15TT’, ‘A3D1 1hTT', ‘A3D1 2hTT’,
‘A3D1 3hTT’, ‘A3D2’): blood pressure diastolic for treatment D

Table D 36: Vital signs (‘A4D1 before’, ‘A4D1 10after’, ‘A4D1 15TT’, ‘A4D1 1hTT', ‘A4D1 2hTT’,
‘A4D1 3hTT’, ‘A4D2’): blood pressure diastolic

Table D 37: Vital signs (‘A4D1 before’, ‘A4D1 10after’, ‘A4D1 15TT’, ‘A4D1 1hTT', ‘A4D1 2hTT’,
‘A4D1 3hTT’, ‘A4D2’): blood pressure diastolic for treatment A

Table D 38: Vital signs (‘A4D1 before’, ‘A4D1 10after’, ‘A4D1 15TT’, ‘A4D1 1hTT', ‘A4D1 2hTT’,
‘A4D1 3hTT’, ‘A4D2’): blood pressure diastolic for treatment B

Table D 39: Vital signs (‘A4D1 before’, ‘A4D1 10after’, ‘A4D1 15TT’, ‘A4D1 1hTT', ‘A4D1 2hTT’,
‘A4D1 3hTT’, ‘A4D2’): blood pressure diastolic for treatment C

Table D 40: Vital signs (‘A4D1 before’, ‘A4D1 10after’, ‘A4D1 15TT’, ‘A4D1 1hTT', ‘A4D1 2hTT’,
‘A4D1 3hTT’, ‘A4D2’): blood pressure diastolic for treatment D

Table D 41: Vital signs (‘A1D1 before’, ‘A1D1 10after’, ‘A1D1 15TT’, ‘A1D1 1hTT', ‘A1D1 2hTT’,
‘A1D1 3hTT’, ‘A1D2’): heart rate

Table D 42: Vital signs (‘A1D1 before’, ‘A1D1 10after’, ‘A1D1 15TT’, ‘A1D1 1hTT', ‘A1D1 2hTT’,
‘A1D1 3hTT’, ‘A1D2’): heart rate for treatment A

Table D 43: Vital signs (‘A1D1 before’, ‘A1D1 10after’, ‘A1D1 15TT’, ‘A1D1 1hTT', ‘A1D1 2hTT’,
‘A1D1 3hTT’, ‘A1D2’): heart rate for treatment B

Table D 44: Vital signs (‘A1D1 before’, ‘A1D1 10after’, ‘A1D1 15TT’, ‘A1D1 1hTT', ‘A1D1 2hTT’,
‘A1D1 3hTT’, ‘A1D2’): heart rate for treatment C

Table D 45: Vital signs (‘A1D1 before’, ‘A1D1 10after’, ‘A1D1 15TT’, ‘A1D1 1hTT', ‘A1D1 2hTT’,
‘A1D1 3hTT’, ‘A1D2’): heart rate for treatment D

Table D 46: Vital signs (‘A2D1 before’, ‘A2D1 10after’, ‘A2D1 15TT’, ‘A2D1 1hTT', ‘A2D1 2hTT’,
‘A2D1 3hTT’, ‘A2D2’): heart rate

Table D 47: Vital signs (‘A2D1 before’, ‘A2D1 10after’, ‘A2D1 15TT’, ‘A2D1 1hTT', ‘A2D1 2hTT’,
‘A2D1 3hTT’, ‘A2D2’): heart rate for treatment A

Table D 48: Vital signs (‘A2D1 before’, ‘A2D1 10after’, ‘A2D1 15TT’, ‘A2D1 1hTT', ‘A2D1 2hTT’,
‘A2D1 3hTT’, ‘A2D2’): heart rate for treatment B

Table D 49: Vital signs (‘A2D1 before’, ‘A2D1 10after’, ‘A2D1 15TT’, ‘A2D1 1hTT', ‘A2D1 2hTT’,
‘A2D1 3hTT’, ‘A2D2’): heart rate for treatment C

Table D 50: Vital signs (‘A2D1 before’, ‘A2D1 10after’, ‘A2D1 15TT’, ‘A2D1 1hTT', ‘A2D1 2hTT’,
‘A2D1 3hTT’, ‘A2D2’): heart rate for treatment D

Table D 51: Vital signs (‘A3D1 before’, ‘A3D1 10after’, ‘A3D1 15TT’, ‘A3D1 1hTT', ‘A3D1 2hTT’,
‘A3D1 3hTT’, ‘A3D2’): heart rate

Table D 52: Vital signs (‘A3D1 before’, ‘A3D1 10after’, ‘A3D1 15TT’, ‘A3D1 1hTT', ‘A3D1 2hTT’,
‘A3D1 3hTT’, ‘A3D2’): heart rate for treatment A

Table D 53: Vital signs (‘A3D1 before’, ‘A3D1 10after’, ‘A3D1 15TT’, ‘A3D1 1hTT', ‘A3D1 2hTT’,
‘A3D1 3hTT’, ‘A3D2’): heart rate for treatment B

Table D 54: Vital signs (‘A3D1 before’, ‘A3D1 10after’, ‘A3D1 15TT’, ‘A3D1 1hTT', ‘A3D1 2hTT’,
‘A3D1 3hTT’, ‘A3D2’): heart rate for treatment C

Table D 55: Vital signs (‘A3D1 before’, ‘A3D1 10after’, ‘A3D1 15TT’, ‘A3D1 1hTT', ‘A3D1 2hTT’,
‘A3D1 3hTT’, ‘A3D2’): heart rate for treatment D

Table D 56: Vital signs (‘A4D1 before’, ‘A4D1 10after’, ‘A4D1 15TT’, ‘A4D1 1hTT', ‘A4D1 2hTT’,
‘A4D1 3hTT’, ‘A4D2’): heart rate

Table D 57: Vital signs (‘A4D1 before’, ‘A4D1 10after’, ‘A4D1 15TT’, ‘A4D1 1hTT', ‘A4D1 2hTT’,
‘A4D1 3hTT’, ‘A4D2’): heart rate for treatment A

Table D 58: Vital signs (‘A4D1 before’, ‘A4D1 10after’, ‘A4D1 15TT’, ‘A4D1 1hTT', ‘A4D1 2hTT’,
‘A4D1 3hTT’, ‘A4D2’): heart rate for treatment B

Table D 59: Vital signs (‘A4D1 before’, ‘A4D1 10after’, ‘A4D1 15TT’, ‘A4D1 1hTT', ‘A4D1 2hTT’,
‘A4D1 3hTT’, ‘A4D2’): heart rate for treatment C

Table D 60: Vital signs (‘A4D1 before’, ‘A4D1 10after’, ‘A4D1 15TT’, ‘A4D1 1hTT', ‘A4D1 2hTT’,
‘A4D1 3hTT’, ‘A4D2’): heart rate for treatment D

Table D 61: Vital signs (A1D1 before’, ‘A1D1 10after’, ‘A1D1 15TT’, ‘A1D1 1hTT', ‘A1D1 2hTT’,
‘A1D1 3hTT’, ‘A1D2’, ‘A2D1 before’, ‘A2D1 10after’, ‘A2D1 15TT’, ‘A2D1 1hTT', ‘A2D1 2hTT’, ‘A2D1 3hTT’, ‘A2D2’, ‘A3D1 before’, ‘A3D1 10after’, ‘A3D1 15TT’, ‘A3D1 1hTT', ‘A3D1 2hTT’, ‘A3D1 3hTT’, ‘A3D2’, ‘A4D1 before’, ‘A4D1 10after’, ‘A4D1 15TT’, ‘A4D1 1hTT', ‘A4D1 2hTT’, ‘A4D1 3hTT’, ‘A4D2’): frequency for ‘Investigator’s decision: subject is qualified to continue?’

Table D 62: Vital signs (A1D1 before’, ‘A1D1 10after’, ‘A1D1 15TT’, ‘A1D1 1hTT', ‘A1D1 2hTT’,
‘A1D1 3hTT’, ‘A1D2’, ‘A2D1 before’, ‘A2D1 10after’, ‘A2D1 15TT’, ‘A2D1 1hTT', ‘A2D1 2hTT’, ‘A2D1 3hTT’, ‘A2D2’, ‘A3D1 before’, ‘A3D1 10after’, ‘A3D1 15TT’, ‘A3D1 1hTT', ‘A3D1 2hTT’, ‘A3D1 3hTT’, ‘A3D2’, ‘A4D1 before’, ‘A4D1 10after’, ‘A4D1 15TT’, ‘A4D1 1hTT', ‘A4D1 2hTT’, ‘A4D1 3hTT’, ‘A4D2’): frequency for ‘Investigator’s decision: subject is qualified to continue?’ for treatment A

Table D 63: Vital signs (A1D1 before’, ‘A1D1 10after’, ‘A1D1 15TT’, ‘A1D1 1hTT', ‘A1D1 2hTT’,
‘A1D1 3hTT’, ‘A1D2’, ‘A2D1 before’, ‘A2D1 10after’, ‘A2D1 15TT’, ‘A2D1 1hTT', ‘A2D1 2hTT’, ‘A2D1 3hTT’, ‘A2D2’, ‘A3D1 before’, ‘A3D1 10after’, ‘A3D1 15TT’, ‘A3D1 1hTT', ‘A3D1 2hTT’, ‘A3D1 3hTT’, ‘A3D2’, ‘A4D1 before’, ‘A4D1 10after’, ‘A4D1 15TT’, ‘A4D1 1hTT', ‘A4D1 2hTT’, ‘A4D1 3hTT’, ‘A4D2’): frequency for ‘Investigator’s decision: subject is qualified to continue?’ for treatment B

Table D 64: Vital signs (A1D1 before’, ‘A1D1 10after’, ‘A1D1 15TT’, ‘A1D1 1hTT', ‘A1D1 2hTT’,
‘A1D1 3hTT’, ‘A1D2’, ‘A2D1 before’, ‘A2D1 10after’, ‘A2D1 15TT’, ‘A2D1 1hTT', ‘A2D1 2hTT’, ‘A2D1 3hTT’, ‘A2D2’, ‘A3D1 before’, ‘A3D1 10after’, ‘A3D1 15TT’, ‘A3D1 1hTT', ‘A3D1 2hTT’, ‘A3D1 3hTT’, ‘A3D2’, ‘A4D1 before’, ‘A4D1 10after’, ‘A4D1 15TT’, ‘A4D1 1hTT', ‘A4D1 2hTT’, ‘A4D1 3hTT’, ‘A4D2’): frequency for ‘Investigator’s decision: subject is qualified to continue?’ for treatment C

Table D 65: Vital signs (A1D1 before’, ‘A1D1 10after’, ‘A1D1 15TT’, ‘A1D1 1hTT', ‘A1D1 2hTT’,
‘A1D1 3hTT’, ‘A1D2’, ‘A2D1 before’, ‘A2D1 10after’, ‘A2D1 15TT’, ‘A2D1 1hTT', ‘A2D1 2hTT’, ‘A2D1 3hTT’, ‘A2D2’, ‘A3D1 before’, ‘A3D1 10after’, ‘A3D1 15TT’, ‘A3D1 1hTT', ‘A3D1 2hTT’, ‘A3D1 3hTT’, ‘A3D2’, ‘A4D1 before’, ‘A4D1 10after’, ‘A4D1 15TT’, ‘A4D1 1hTT', ‘A4D1 2hTT’, ‘A4D1 3hTT’, ‘A4D2’): frequency for ‘Investigator’s decision: subject is qualified to continue?’ for treatment D

**Respiratory testing:**

See Table C79, …, Table C156

**Adverse events:**

Table D 66: Listing of adverse events (screening phase)

Table D 67: Listing of serious adverse events (screening phase)

Table D 68: Listing of SUSARs (screening phase)

Table D 69: Listing of adverse events (treatment phase)

Table D 70: Listing of serious adverse events (treatment phase)

Table D 71: Listing of SUSARs (treatment phase)

Table D 72: Proportion of participants with at least one AE / SAE / SUSAR (incl. 95% confidence interval) for each study arm A, B, C, D

The tables Table D1, …, Table D 65 will be provided for whole study population and by stratum male/female.

## Figures

Figure 1: Overview and time course of the ELSA trial

Figure 2: Participants recruitment over time

Figure 3: Participants flow chart

Boxplots will be created (in case of enough data) for each endpoint and other important continuous parameter by e.g. sex, by treatment, by point in time, and by sex / treatment. In case of not enough data (group sizes <10), a scatter plot will be provided instead.
