# Supplementary material for: The ELSA trial: single versus combinatory effects of non-prohibited beta-2 agonists on skeletal muscle metabolism, cardio-pulmonary function and endurance performance—study protocol for a randomized 4-way balanced cross-over trial
Source: Trials. 2021 Dec 11;22:903. doi: 10.1186/s13063-021-05862-w (PMC8665595; doi:10.1186/s13063-021-05862-w)
Supplement: Supplementary file 3 — Additional file 3. SPIRIT checklist for Trials. [file 13063_2021_5862_MOESM3_ESM.pdf]

SPIRIT Checklist for *Trials*

Complete this checklist by entering the page and line numbers where each of the items listed below can be found in your manuscript.

Your manuscript may not currently address all the items on the checklist. Please modify your text to include the missing information. If you are certain that an item does not apply, please state "n/a" and provide a short explanation. **Leaving an item blank or stating "n/a" without an explanation will lead to your manuscript being returned before review.**

Upload your completed checklist as an additional file when you submit to *Trials*. You must reference this additional file in the main text of your protocol submission. The completed SPIRIT figure must be included within the main body of the protocol text and can be downloaded here: http://www.spirit-statement.org/schedule-of-enrolment-interventions-and-assessments/

In your methods section, please state that you used the SPIRIT reporting guidelines, and cite them as:

Chan A-W, Tetzlaff JM, Gøtzsche PC, Altman DG, Mann H, Berlin J, Dickersin K, Hróbjartsson A, Schulz KF, Parulekar WR, Krleža-Jerić K, Laupacis A, Moher D. SPIRIT 2013 Explanation and Elaboration: Guidance for protocols of clinical trials. BMJ. 2013;346:e7586

		Reporting Item	Page and Line Number	Reason if not applicable
**Administrative information**				
Title	#1	Descriptive title identifying the study design, population, interventions, and, if applicable, trial acronym	[p1, lines 1 – 4]	
Trial registration	#2a	Trial identifier and registry name. If not yet registered, name of intended registry	[p3, lines 58 – 61; p19 line 473]	
Trial registration: data set	#2b	All items from the World Health Organization Trial Registration Data Set	[Please refer to Item 2a and registration in the European Clinical Trials Database (Eudra 58 CT) with the number: 2015-	

			005598-19 as well as at the German register for clinical studies (DRKS number 00010574)]	
Protocol version	#3	Date and version identifier	[p18, line 450]	
Funding	#4	Sources and types of financial, material, and other support	[p19, line 483 – p20, line 491]	
Roles and responsibilities: contributorship	#5a	Names, affiliations, and roles of protocol contributors	[p1, lines 6 – 19; p21, lines 522 – 527]	
Roles and responsibilities: sponsor contact information	#5b	Name and contact information for the trial sponsor	[p19, lines 483 – 484]	
Roles and responsibilities: sponsor and funder	#5c	Role of study sponsor and funders, if any, in study design; collection, management, analysis, and interpretation of data; writing of the report; and the decision to submit the report for publication, including whether they will have ultimate authority over any of these activities	[p19, lines 483 – p20, lines 489; p21 lines 514– 515]	
Roles and responsibilities: committees	#5d	Composition, roles, and responsibilities of the coordinating centre, steering committee, endpoint adjudication committee, data management team, and other individuals or groups overseeing the trial, if applicable (see Item 21a for data monitoring committee)	[p7, lines 175 – 191; p8, lines 206 – 208; p12, lines 301 – 303; p19, lines 475 – 477; p20, lines 500 – p21, lines 519]	
**Introduction**				

Background and rationale	#6a	Description of research question and justification for undertaking the trial, including summary of relevant studies (published and unpublished) examining benefits and harms for each intervention	[p3, lines 67 – p5, lines 127]	
Background and rationale: choice of comparators	#6b	Explanation for choice of comparators	[p4, lines 94 – p5, lines 127]	
Objectives	#7	Specific objectives or hypotheses	[p5, lines 124 – 127; p18, lines 441 – 447]	
Trial design	#8	Description of trial design including type of trial (eg, parallel group, crossover, factorial, single group), allocation ratio, and framework (eg, superiority, equivalence, non-inferiority, exploratory)	[p5, lines 130 – p6, lines 147; Fig. 1; p6, lines 149 – p7, lines 168]	
**Methods: Participants, interventions, and outcomes**				
Study setting	#9	Description of study settings (eg, community clinic, academic hospital) and list of countries where data will be collected. Reference to where list of study sites can be obtained	[p5, line 137; p8, lines 207– 208]	
Eligibility criteria	#10	Inclusion and exclusion criteria for participants. If applicable, eligibility criteria for study centres and individuals who will perform the interventions (eg, surgeons, psychotherapists)	[p8, lines 197 – 206 + supplementary material]	

Interventions: description	#11a	Interventions for each group with sufficient detail to allow replication, including how and when they will be administered	[p5, lines 130 – p12, lines 293]	
Interventions: modifications	#11b	Criteria for discontinuing or modifying allocated interventions for a given trial participant (eg, drug dose change in response to harms, participant request, or improving / worsening disease)	[p17, lines 430 – 432; p18, lines 435 – 436; p20, lines 503 -p21, lines 515]	
Interventions: adherance	#11c	Strategies to improve adherence to intervention protocols, and any procedures for monitoring adherence (eg, drug tablet return; laboratory tests)	[p7, lines 182 – 185; p8, lines 212 – 214; p9, lines 233 - 237; p10, lines 251 – p11, lines 278; p12, lines 289 – 293; p20, lines 492 – 499; Supplementary material; p5, lines 134 – 136]	
Interventions: concomitant care	#11d	Relevant concomitant care and interventions that are permitted or prohibited during the trial	[p7, lines 186 – p8, lines 191; p10, lines 248-260 p11, lines 279 – 280; p12, lines 289 – 293; Supplementary material p4, lines 106 – p5, lines 131; p5, lines 134 – 136]	
Outcomes	#12	Primary, secondary, and other outcomes, including the specific measurement variable (eg, systolic blood pressure), analysis metric (eg, change from baseline, final value, time to event),	[p12, lines 295 – p16, lines 397]	

		method of aggregation (eg, median, proportion), and time point for each outcome. Explanation of the clinical relevance of chosen efficacy and harm outcomes is strongly recommended		
Participant timeline	#13	Time schedule of enrolment, interventions (including any run-ins and washouts), assessments, and visits for participants. A schematic diagram is highly recommended (see Figure)	[Fig. 1 on page 6; Table 1 on page 10; p8, lines 209 – 211; p9 lines 224 – p10 lines 254; p11, lines 285 – page 12 lines 288]	
Sample size	#14	Estimated number of participants needed to achieve study objectives and how it was determined, including clinical and statistical assumptions supporting any sample size calculations	[p17, lines 411 – p18, lines 438]	
Recruitment	#15	Strategies for achieving adequate participant enrolment to reach target sample size	[p5, lines 137 – p6 lines 147; p20, lines 492 – 499]	
**Methods: Assignment of interventions (for controlled trials)**				
Allocation: sequence generation	#16a	Method of generating the allocation sequence (eg, computer-generated random numbers), and list of any factors for stratification. To reduce predictability of a random sequence, details of any planned restriction (eg, blocking) should be provided in a separate document that is unavailable to those who enrol participants or assign interventions	[p7, lines 171 – 179]	

Allocation concealment mechanism	#16b	Mechanism of implementing the allocation sequence (eg, central telephone; sequentially numbered, opaque, sealed envelopes), describing any steps to conceal the sequence until interventions are assigned	[p7, lines 171 – 179; p7, lines 182 – 184; p 20, lines 469 - 471	
Allocation: implementation	#16c	Who will generate the allocation sequence, who will enrol participants, and who will assign participants to interventions	[p7, lines 175 – 177; p8, lines 203 – 208; p 17, lines 413 – 415]	
Blinding (masking)	#17a	Who will be blinded after assignment to interventions (eg, trial participants, care providers, outcome assessors, data analysts), and how	[p2, lines 39 - 42; p5, lines 123 – 124; p7, lines 162 – 164; p7, lines 167 – p8, 175; p8, lines 187 – 192; p12; lines 271 - 272; p 18; lines 412 – 414; p20, lines 469 – 471]	
Blinding (masking): emergency unblinding	#17b	If blinded, circumstances under which unblinding is permissible, and procedure for revealing a participant's allocated intervention during the trial	[p7, lines 185 – p8, lines 191]	
**Methods: Data collection, management, and analysis**				
Data collection plan	#18a	Plans for assessment and collection of outcome, baseline, and other trial data, including any related processes to promote data quality (eg, duplicate measurements, training of assessors) and a description of study instruments (eg, questionnaires, laboratory tests) along with their	[p12 line 296 – p16, line 397]	

		reliability and validity, if known. Reference to where data collection forms can be found, if not in the protocol		
Data collection plan: retention	#18b	Plans to promote participant retention and complete follow-up, including list of any outcome data to be collected for participants who discontinue or deviate from intervention protocols	[p8, lines 212 – 214; p 17, lines 430 – p18 lines 436; p20, lines 492 – 499]	
Data management	#19	Plans for data entry, coding, security, and storage, including any related processes to promote data quality (eg, double data entry; range checks for data values). Reference to where details of data management procedures can be found, if not in the protocol	[p10, lines 248 – 249; p12, lines 298– 303]	
Statistics: outcomes	#20a	Statistical methods for analysing primary and secondary outcomes. Reference to where other details of the statistical analysis plan can be found, if not in the protocol	[p17, lines 412 – p18, lines 438]	
Statistics: additional analyses	#20b	Methods for any additional analyses (eg, subgroup and adjusted analyses)	[p17, lines 412 – p18, lines 438]	
Statistics: analysis population and missing data	#20c	Definition of analysis population relating to protocol non-adherence (eg, as randomised analysis), and any statistical methods to handle missing data (eg, multiple imputation)	[p17, lines 412 – p18, lines 438]	
**Methods: Monitoring**				

Data monitoring: formal committee	#21a	Composition of data monitoring committee (DMC); summary of its role and reporting structure; statement of whether it is independent from the sponsor and competing interests; and reference to where further details about its charter can be found, if not in the protocol. Alternatively, an explanation of why a DMC is not needed	[p20, lines 500 – p21, lines 515]	
Data monitoring: interim analysis	#21b	Description of any interim analyses and stopping guidelines, including who will have access to these interim results and make the final decision to terminate the trial	[p20, lines 500 – p21, lines 515]	
Harms	#22	Plans for collecting, assessing, reporting, and managing solicited and spontaneously reported adverse events and other unintended effects of trial interventions or trial conduct	[p20, lines 507 – 510]	
Auditing	#23	Frequency and procedures for auditing trial conduct, if any, and whether the process will be independent from investigators and the sponsor	[p21, lines 516 – 521]	
**Ethics and dissemination**				
Research ethics approval	#24	Plans for seeking research ethics committee / institutional review board (REC / IRB) approval	[p19, lines 473 – 477]	
Protocol amendments	#25	Plans for communicating important protocol modifications (eg, changes to eligibility criteria, outcomes, analyses) to relevant parties (eg,	[p10, lines 249 – 250; p20, lines 503 – p21 lines 513]	

		investigators, REC / IRBs, trial participants, trial registries, journals, regulators)		
Consent or assent	#26a	Who will obtain informed consent or assent from potential trial participants or authorised surrogates, and how (see Item 32)	[p16, lines 404 – 407]	
Consent or assent: ancillary studies	#26b	Additional consent provisions for collection and use of participant data and biological specimens in ancillary studies, if applicable		n/a, participant data or biological specimens will not be used in ancillary studies]
Confidentiality	#27	How personal information about potential and enrolled participants will be collected, shared, and maintained in order to protect confidentiality before, during, and after the trial	[p12, lines 298 – 303; p17, lines 408 – 409]	
Declaration of interests	#28	Financial and other competing interests for principal investigators for the overall trial and each study site	[p19, line 482]	
Data access	#29	Statement of who will have access to the final trial dataset, and disclosure of contractual agreements that limit such access for investigators	[p3, lines 55 – 57]	
Ancillary and post trial care	#30	Provisions, if any, for ancillary and post-trial care, and for compensation to those who suffer harm from trial participation	[p8, lines 212 – 214; p20 lines 496-499]	
Dissemination policy: trial results	#31a	Plans for investigators and sponsor to communicate trial results to participants, healthcare professionals, the public, and other relevant groups (eg, via publication, reporting in	[p3, lines 55 – 57]; p19, lines 479-481]	

		results databases, or other data sharing arrangements), including any publication restrictions		
Dissemination policy: authorship	#31b	Authorship eligibility guidelines and any intended use of professional writers	[p21, lines 522-527]	
Dissemination policy: reproducible research	#31c	Plans, if any, for granting public access to the full protocol, participant-level dataset, and statistical code	[p19, lines 479-481]	
**Appendices**				
Informed consent materials	#32	Model consent form and other related documentation given to participants and authorised surrogates		n/a, the detailed study information including the consent form are only available in German language, since the study is only conducted in Germany. The consent form can be translated and made available on request.
Biological specimens	#33	Plans for collection, laboratory evaluation, and storage of biological specimens for genetic or molecular analysis in the current trial and for future use in ancillary studies, if applicable	[p12, lines 305 – p14, lines 351]	

It is strongly recommended that this checklist be read in conjunction with the SPIRIT 2013 Explanation & Elaboration for important clarification on the items. Amendments to the protocol should be tracked and dated. The SPIRIT checklist is copyrighted by the SPIRIT Group under the Creative Commons "Attribution-NonCommercial-NoDerivs 3.0 Unported" license. This checklist can be completed online using https://www.goodreports.org/, a tool made by the EQUATOR Network in collaboration with Penelope.ai
